# Co-expression networks reveal the tissue-specific regulation of transcription and splicing

**DOI:** 10.1101/gr.216721.116

**Published:** 2017-11

**Authors:** Ashis Saha, Yungil Kim, Ariel D.H. Gewirtz, Brian Jo, Chuan Gao, Ian C. McDowell, The GTEx Consortium, Barbara E. Engelhardt, Alexis Battle

**Affiliations:** 1 Department of Computer Science, Johns Hopkins University, Baltimore, Maryland 21218, USA;; 2 Program in Quantitative and Computational Biology, Princeton University, Princeton, New Jersey 08540, USA;; 3 Department of Statistical Science, Duke University, Durham, North Carolina 27708, USA;; 4 Program in Computational Biology and Bioinformatics, Duke University, Durham, North Carolina 27708, USA;; 5 Department of Computer Science and Center for Statistics and Machine Learning, Princeton University, Princeton, New Jersey 08540, USA; 8 The Broad Institute of Massachusetts Institute of Technology and Harvard University, Cambridge, MA 02142, USA; 9 Analytic and Translational Genetics Unit, Massachusetts General Hospital, Boston, MA 02114, USA; 10 Massachusetts General Hospital Cancer Center and Dept. of Pathology, Massachusetts General Hospital, Boston, MA 02114, USA; 11 Department of Genetics, Harvard Medical School, Boston, MA 02114, USA; 12 Department of Genetics, Stanford University, Stanford, CA 94305, USA; 13 Department of Pathology, Stanford University, Stanford, CA 94305, USA; 14 Department of Clinical Biochemistry and Pharmacology, Faculty of Health Sciences, Ben-Gurion University of the Negev, Beer-Sheva 84105, Israel; 15 Department of Computer Science, Johns Hopkins University, Baltimore, MD 21218, USA; 16 Centre for Genomic Regulation (CRG), The Barcelona Institute for Science and Technology, 08003 Barcelona, Spain; 17 Universitat Pompeu Fabra (UPF), 08003 Barcelona, Spain; 18 Department of Genetic Medicine and Development, University of Geneva Medical School, 1211 Geneva, Switzerland; 19 Institute for Genetics and Genomics in Geneva (iG3), University of Geneva, 1211 Geneva, Switzerland; 20 Swiss Institute of Bioinformatics, 1211 Geneva, Switzerland; 21 Department of Genetics, Perelman School of Medicine, University of Pennsylvania, Philadelphia, PA 19104, USA; 22 New York Genome Center, New York, NY 10013, USA; 23 Department of Systems Biology, Columbia University Medical Center, New York, NY 10032, USA; 24 Department of Public Health Sciences, The University of Chicago, Chicago, IL 60637, USA; 25 McDonnell Genome Institute, Washington University School of Medicine, St. Louis, MO 63108, USA; 26 Department of Genetics, Washington University School of Medicine, St. Louis, MO 63108, USA; 27 Department of Pathology & Immunology, Washington University School of Medicine, St. Louis, MO 63108, USA; 28 Division of Genetic Medicine, Department of Medicine, Vanderbilt University Medical Center, Nashville, TN 37232, USA; 29 Department of Computer Science, Center for Statistics and Machine Learning, Princeton University, Princeton, NJ 08540, USA; 30 Department of Computer Science, University of California, Los Angeles, CA 90095, USA; 31 Department of Human Genetics, University of California, Los Angeles, CA 90095, USA; 32 Instituto de Investigação e Inovação em Saúde (i3S), Universidade do Porto, 4200-135 Porto, Portugal; 33 Institute of Molecular Pathology and Immunology (IPATIMUP), University of Porto, 4200-625 Porto, Portugal; 34 Department of Clinical Epidemiology, Biostatistics and Bioinformatics, Academic Medical Center, University of Amsterdam, 1105 AZ Amsterdam, The Netherlands; 35 Department of Psychiatry, Academic Medical Center, University of Amsterdam, 1105 AZ Amsterdam, The Netherlands; 36 Lewis Sigler Institute, Princeton University, Princeton, NJ 08540, USA; 37 Department of Operations Research and Financial Engineering, Princeton University, Princeton, NJ 08540, USA; 38 Biomedical Informatics Program, Stanford University, Stanford, CA 94305, USA; 39 Institut Hospital del Mar d'Investigacions Mèdiques (IMIM), 08003 Barcelona, Spain; 40 Department of Medicine, Washington University School of Medicine, St. Louis, MO 63108, USA; 41 Department of Convergence Medicine, University of Ulsan College of Medicine, Asan Medical Center, Seoul 138-736, South Korea; 42 Department of Biomedical Engineering, Johns Hopkins University, Baltimore, MD 21218, USA; 43 Section of Genetic Medicine, Department of Medicine, The University of Chicago, Chicago, IL 60637, USA; 44 Department of Biostatistics, Mailman School of Public Health, Columbia University, New York, NY 10032, USA; 45 Department of Biology, Stanford University, Stanford, CA 94305, USA; 46 Wellcome Trust Centre for Human Genetics, Nuffield Department of Medicine, University of Oxford, Oxford, OX3 7BN, UK; 47 Oxford Centre for Diabetes, Endocrinology and Metabolism, University of Oxford, Churchill Hospital, Oxford, OX3 7LE, UK; 48 Oxford NIHR Biomedical Research Centre, Churchill Hospital, Oxford, OX3 7LJ, UK; 49 Computational Biology & Bioinformatics Graduate Program, Duke University, Durham, NC 27708, USA; 50 Human Genetics Department, McGill University, Montreal, Quebec H3A 0G1, Canada; 51 Departament d'Estadística i Investigació Operativa, Universitat Politècnica de Catalunya, 08034 Barcelona, Spain; 52 Department of Statistics, The University of Chicago, Chicago, IL 60637, USA; 53 Department of Human Genetics, The University of Chicago, Chicago, IL 60637, USA; 54 Department of Statistics and Operations Research, University of North Carolina, Chapel Hill, NC 27599, USA; 55 Department of Biostatistics, University of North Carolina, Chapel Hill, NC 27599, USA; 56 Institute for Genomics and Systems Biology, The University of Chicago, Chicago, IL 60637, USA; 57 Department of Biostatistics, The University of Texas MD Anderson Cancer Center, Houston, TX 77030, USA; 58 Computational Sciences, Pfizer Inc, Cambridge, MA 02139, USA; 59 Universitat de Barcelona, 08028 Barcelona, Catalonia, Spain; 60 Department of Biomedical Data Science, Stanford University, Stanford, CA 94305, USA; 61 Department of Statistics, Stanford University, Stanford, CA 94305, USA; 62 Institute of Biophysics Carlos Chagas Filho (IBCCF), Federal University of Rio de Janeiro (UFRJ), 21941902 Rio de Janeiro, Brazil; 63 Department of Psychiatry, University of Utah, Salt Lake City, UT 84108, USA; 64 Center for Data Intensive Science, The University of Chicago, Chicago, IL 60637, USA; 65 Department of Psychiatry and Biobehavioral Sciences, University of California, Los Angeles, CA 90095, USA; 66 Department of Biostatistics, University of Michigan, Ann Arbor, MI 48109, USA; 67 Bioinformatics Research Center and Departments of Statistics and Biological Sciences, North Carolina State University, Raleigh, NC 27695, USA; 68 National Institute for Biotechnology in the Negev, Beer-Sheva, 84105 Israel; 69 European Molecular Biology Laboratory, 69117 Heidelberg, Germany; 70 Department of Ecology and Evolutionary Biology, Princeton University, Princeton, NJ 08540, USA; 71 Altius Institute for Biomedical Sciences, Seattle, WA 98121, USA; 72 Beth Israel Deaconess Medical Center, Harvard Medical School, Boston, MA 02215, USA; 73 University of Hohenheim, 70599 Stuttgart, Germany; 74 Huntsman Cancer Institute, Department of Population Health Sciences, University of Utah, Salt Lake City, UT 84112, USA; 75 Center for Epigenetics, Johns Hopkins University School of Medicine, Baltimore, MD 21205, USA; 76 Department of Medicine, Johns Hopkins University School of Medicine, Baltimore, MD 21205, USA; 77 Department of Mental Health, Johns Hopkins University School of Public Health, Baltimore, MD 21205, USA; 78 McKusick-Nathans Institute of Genetic Medicine, Johns Hopkins School of Medicine, Baltimore, MD 21205, USA; 79 Department of Biostatistics, Johns Hopkins University, Baltimore, MD 21205, USA; 80 Computer Science and Artificial Intelligence Laboratory, Massachusetts Institute of Technology, Cambridge, MA 02139, USA; 81 Department of Medicine, University of Washington, Seattle, WA 98195, USA; 82 Division of Cardiology, University of Washington, Seattle, WA 98195, USA; 83 Institute for Systems Genetics, New York University Langone Medical Center, New York, NY 10016, USA; 84 Department of Genome Sciences, University of Washington, Seattle, WA 98195, USA; 85 Office of Strategic Coordination, Division of Program Coordination, Planning and Strategic Initiatives, Office of the Director, NIH, Rockville, MD 20852, USA; 86 Biorepositories and Biospecimen Research Branch, Division of Cancer Treatment and Diagnosis, National Cancer Institute, Bethesda, MD 20892, USA; 87 National Institute of Dental and Craniofacial Research, Bethesda, MD 20892, USA; 88 Division of Genomic Medicine, National Human Genome Research Institute, Rockville, MD 20852, USA; 89 Division of Neuroscience and Basic Behavioral Science, National Institute of Mental Health, NIH, Bethesda, MD 20892, USA; 90 Division of Neuroscience and Behavior, National Institute on Drug Abuse, NIH, Bethesda, MD 20892, USA; 91 Washington Regional Transplant Community, Falls Church, VA 22003, USA; 92 Gift of Life Donor Program, Philadelphia, PA 19103, USA; 93 LifeGift, Houston, TX 77055, USA; 94 Center for Organ Recovery and Education, Pittsburgh, PA 15238, USA; 95 LifeNet Health, Virginia Beach, VA 23453, USA; 96 National Disease Research Interchange, Philadelphia, PA 19103, USA; 97 Unyts, Buffalo, NY 14203, USA; 98 Pharmacology and Therapeutics, Roswell Park Cancer Institute, Buffalo, NY 14263, USA; 99 Van Andel Research Institute, Grand Rapids, MI 49503, USA; 100 Brain Endowment Bank, Miller School of Medicine, University of Miami, Miami, FL 33136, USA; 101 National Institute of Allergy and Infectious Diseases, NIH, Rockville, MD 20852, USA; 102 Biospecimen Research Group, Clinical Research Directorate, Leidos Biomedical Research, Inc., Rockville, MD 20852, USA; 103 Leidos Biomedical Research, Inc., Frederick, MD 21701, USA; 104 Temple University, Philadelphia, PA 19122, USA; 105 Department of Health Behavior and Policy, School of Medicine, Virginia Commonwealth University, Richmond, VA 23298, USA; 106 European Molecular Biology Laboratory, European Bioinformatics Institute, Hinxton CB10 1SD, UK; 107 UCSC Genomics Institute, University of California Santa Cruz, Santa Cruz, CA 95064, USA

## Abstract

Gene co-expression networks capture biologically important patterns in gene expression data, enabling functional analyses of genes, discovery of biomarkers, and interpretation of genetic variants. Most network analyses to date have been limited to assessing correlation between total gene expression levels in a single tissue or small sets of tissues. Here, we built networks that additionally capture the regulation of relative isoform abundance and splicing, along with tissue-specific connections unique to each of a diverse set of tissues. We used the Genotype-Tissue Expression (GTEx) project v6 RNA sequencing data across 50 tissues and 449 individuals. First, we developed a framework called Transcriptome-Wide Networks (TWNs) for combining total expression and relative isoform levels into a single sparse network, capturing the interplay between the regulation of splicing and transcription. We built TWNs for 16 tissues and found that hubs in these networks were strongly enriched for splicing and RNA binding genes, demonstrating their utility in unraveling regulation of splicing in the human transcriptome. Next, we used a Bayesian biclustering model that identifies network edges unique to a single tissue to reconstruct Tissue-Specific Networks (TSNs) for 26 distinct tissues and 10 groups of related tissues. Finally, we found genetic variants associated with pairs of adjacent nodes in our networks, supporting the estimated network structures and identifying 20 genetic variants with distant regulatory impact on transcription and splicing. Our networks provide an improved understanding of the complex relationships of the human transcriptome across tissues.

Gene co-expression networks are an essential framework for elucidating gene function and interactions, identifying sets of genes that respond in a coordinated way to environmental and disease conditions, and highlighting regulatory relationships (Penrod et al. 2011; Xiao et al. 2014; Yang et al. 2014). Each edge in a co-expression network reflects a correlation between two transcriptional products, represented as nodes (Stuart et al. 2003). Most gene co-expression networks focus on correlation between total gene expression levels, with edges representing transcriptional coregulation. However, posttranscriptional modifications, including alternative splicing, are important in creating a transcriptome with diverse biological functions (Matlin et al. 2005). Mutations that lead to disruption of splicing play an important role in tissue- and disease-specific pathways (López-Bigas et al. 2005; Wang et al. 2008; Ward and Cooper 2010; Lee et al. 2012; DeBoever et al. 2015; Li et al. 2016c).

While a number of splicing factors are known, regulation of splicing and specific regulatory genes involved remain poorly understood relative to the regulation of transcription (Melé et al. 2015; Scotti and Swanson 2015). Although abundance of different isoforms can be influenced by processes including usage of alternative transcription start or end sites and RNA degradation, variation in isoform levels is often the direct result of alternative splicing. RNA sequencing (RNA-seq) now allows quantification of isoform-level expression, providing an opportunity to study regulation of splicing using a network analysis. However, current research estimating RNA isoform-level networks (Li et al. 2014, 2015, 2016a) has focused on total expression of each isoform, and the resulting network structures do not distinguish between regulation of transcription and regulation of splicing in an interpretable way. Initial work on clustering relative isoform abundances has also been explored (Dai et al. 2012; Iancu et al. 2015) but does not support discovery of fine-grained network structure or identification of regulatory genes. Neither approach has been applied to large RNA-seq studies for network reconstruction in diverse tissues.

Another important gap in our interpretation of regulatory effects in complex traits is a global characterization of co-expression relationships that are only present in a specific tissue type. Per-tissue networks have been estimated for multiple tissues (Piro et al. 2011; Pierson et al. 2015), but, critically, these analyses do not directly separate effects unique to each tissue from effects shared across all or many tissues. Recent studies have recognized the essential role that tissue-specific pathways play in disease etiology (Greene et al. 2015) but have developed these per-tissue networks by aggregating single tissue samples across multiple studies. However, differences in study design, technical effects, and tissue-specific expression make cross-study results difficult to interpret mechanistically, with large groups of genes expressed in similar tissues and studies tending to be highly connected rather than including sparse edges that detail tissue-specific network structure (Lee et al. 2004).

In this work, we reconstruct co-expression networks from the Genotype Tissue Expression (GTEx) v6 RNA-seq data (The GTEx Consortium 2015, 2017), including 449 human donors with genotype information and 7310 RNA-seq samples across 50 tissues. We apply computational methods designed to reveal novel relationships between genes and across tissues as compared to previous analyses, specifically addressing two important goals in regulatory biology: identification of edges reflecting regulation of splicing, and discovery of edges arising from gene relationships unique to specific tissues. We introduce a new framework, Transcriptome-Wide Networks (TWNs), which captures gene relationships that reflect regulation of alternative splicing in an interpretable model. We built TWNs to identify candidate regulators of both splicing and transcription across 16 tissues. Next, we identified Tissue-Specific Networks (TSNs) for 26 tissues, where each network edge corresponds to a correlation between genes that is uniquely found in a single tissue. We study the biological interpretation of both network types by quantifying enrichment of known biological functions among well-connected nodes. Finally, we use genetic variation to validate network edges from each network by testing associations between a regulatory variant local to one gene with that gene's network neighbors. Interpretation of regulatory and disease studies will benefit greatly from these networks, providing a much more comprehensive description of regulatory processes, including alternative splicing across diverse tissues.

## Results

### Reconstructing Transcriptome-Wide Networks across human tissues

**Figure 1.**
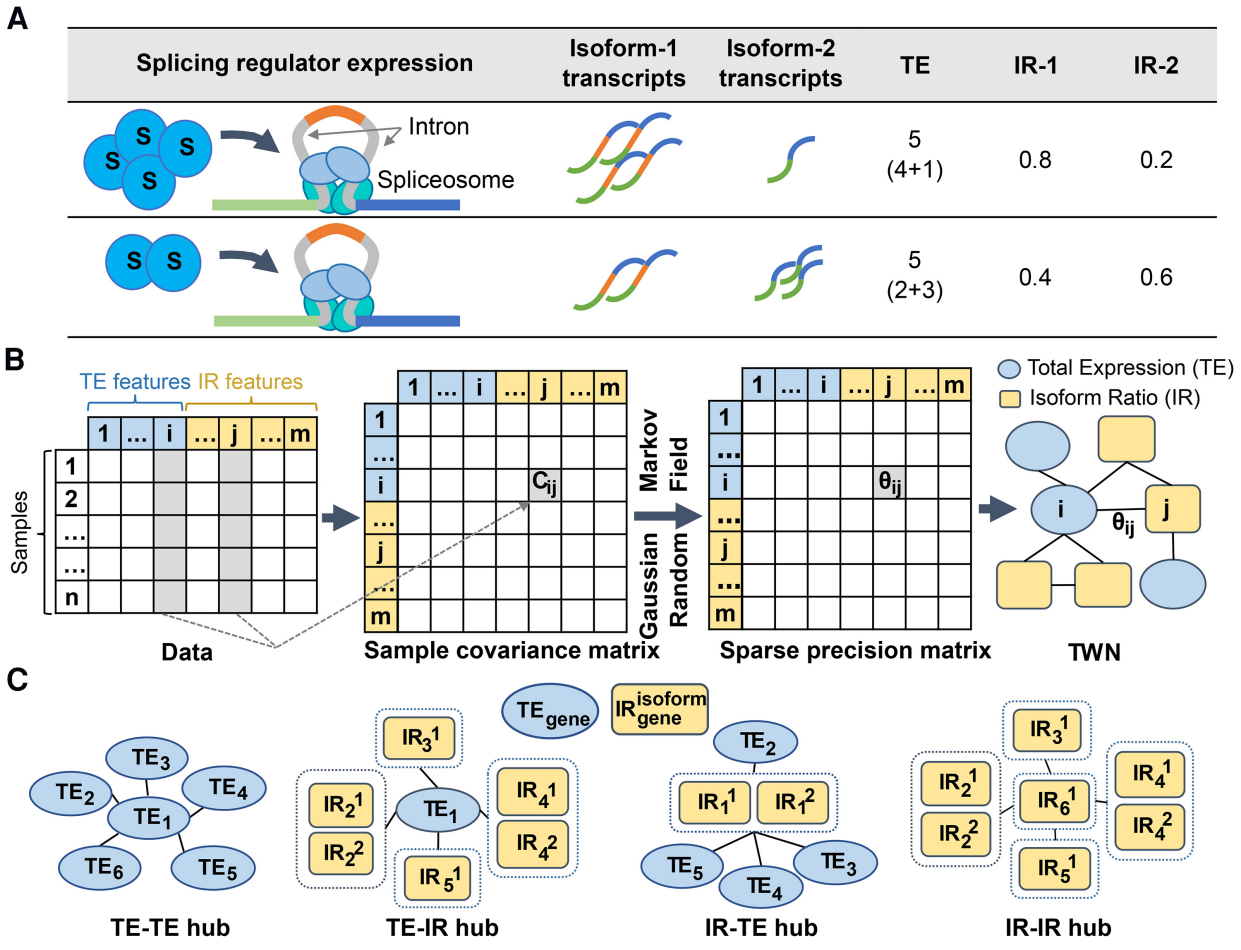
Transcriptome-Wide Network conceptual framework. (*A*) Schematic of the effect of a splicing regulator on inclusion of a cassette exon and resulting total expression and isoform ratios of the target gene. Splicing factor expression levels can affect splicing of target genes (Sveen et al. 2015). Higher expression of a splicing regulator S (first row) results in relatively more transcripts of isoform-1 and fewer of isoform-2. Total expression level is constant (5), but isoform ratios are different (0.4 and 0.6) as splicing factor S levels change (second row). (*B*) The (*i*,*j*)th cell of the sample covariance matrix contains covariance (*C*_*ij*_) between the *i*th and *j*th feature in data. We created a sparse precision matrix Θ (inverse covariance) from the sample covariance matrix using a graphical lasso to estimate the parameters of a Gaussian Markov random field. A nonzero value (Θ_*ij*_) in the precision matrix denotes an edge between the *i*th feature and *j*th feature in the network. (*C*) Edges in a TWN represent diverse relationships between total expression (TE) and isoform ratio (IR) nodes. Dotted rectangles group together isoform ratios for different isoforms of the same gene. Of particular focus are network “hub” nodes; in a TWN, there are four possible hub configurations depending on the node type of the central and neighboring nodes.

First, we aimed to identify networks that capture a global view of regulation across the transcriptome of diverse human tissues using the GTEx project v6 data (The GTEx Consortium 2017). We developed an approach for estimating Transcriptome-Wide Networks from RNA-seq data, which captures diverse regulatory relationships beyond co-expression, including coregulation of alternative splicing across multiple genes. To build a TWN, we first quantified both total expression levels and isoform expression levels of each gene in each RNA-seq sample and then computed isoform ratios (Fig. 1A), representing the relative, rather than total, abundance of each isoform with respect to the total expression of the gene (Methods). We included both isoform ratios (IRs) and total expression levels (TEs) as network nodes, as opposed to estimating a standard correlation network across expression levels of each isoform. This difference is critical to distinguishing correlation due to regulation of splicing (or other posttranscriptional effects) from correlation due to regulation of transcription. While transcriptional regulation affects total expression of a gene and regulation of splicing primarily affects isoform ratios rather than total expression, both mechanisms affect the expression level of each isoform. Therefore, a standard isoform level network confounds these regulatory mechanisms, and network edges cannot be directly interpreted to inform regulation of splicing.

For example, to represent the relationship between a transcription factor (TF) and expression of a target gene, where all isoforms are equally affected, a standard network would require edges from each isoform level of the TF to each isoform level of the target. The same structure would be required to capture the relationship between a splicing factor (SF) and its target gene, where transcription may not be grossly affected but relative production of isoforms is altered (Sveen et al. 2015). In contrast, in a TWN, a TF would only be connected to the total expression of its target, and a SF would be connected only to target isoform ratios (Fig. 1C; Supplemental Fig. S1). TWNs can be more easily interpreted, automatically predicting specific biological relationships, including regulation of relative isoform abundance.

Before estimating TWNs, all total expression and isoform ratio values were separately projected onto quantiles of a standard normal distribution. We then applied a graphical lasso (Friedman et al. 2008) to estimate edge weights of a sparse Gaussian Markov random field (GMRF) (Rue and Held 2005) over all nodes jointly, including both the total expression of each gene and the isoform ratio for each isoform (Fig. 1B; Methods). A GMRF captures direct relationships between nodes—a nonzero entry in the precision matrix (interpreted as an edge between two nodes) indicates that the nodes are correlated after controlling for effects of all other nodes in the network (i.e., a partial correlation) (Schäfer and Strimmer 2005a). We modified the graphical lasso to penalize edges between different node types with different weights (Methods; Supplemental Table S1; Supplemental Figs. S2, S3).

We reconstructed TWNs independently for each of 16 tissues from the GTEx data, restricting to tissues with samples from at least 200 donors (Supplemental Data S1). We focused on a subset of 6000 TE and 9000 IR nodes for each tissue, based on expression levels, gene mappability, and isoform variability (Methods). We excluded Chromosome Y, noncoding genes, and mitochondrial genes. Both technical and biological confounding factors may introduce correlations among genes (Leek et al. 2010), resulting in false positives in co-expression network analysis (Buettner et al. 2015). Therefore, before applying the graphical lasso, we corrected expression data from each tissue for known and unobserved confounding factors using HCP (Mostafavi et al. 2013; Methods). Additionally, after applying the graphical lasso, we excluded edges that were unlikely to represent meaningful biological relationships, such as edges connecting gene pairs with overlapping positions in the genome, edges connecting gene pairs with cross-mapping potential, and edges between distinct features of the same gene (Methods).

**Figure 2.**
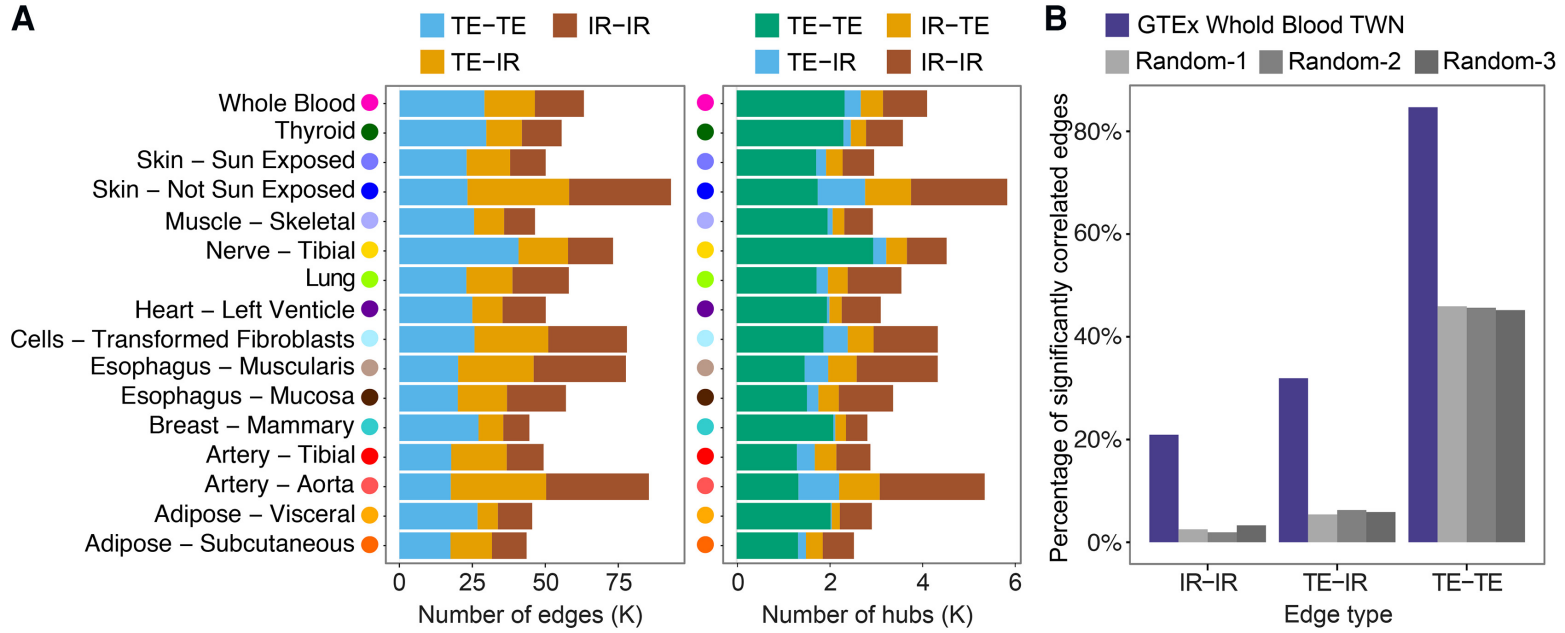
GTEx Transcriptome-Wide Networks summary and replication. (*A*) For each tissue, number of edges and number of hub nodes (≥10 neighbors), segmented by the type of nodes connected by each edge. A “TE-IR” hub is a TE node with multiple IR neighbors, and an “IR-TE” hub is an IR node with multiple TE neighbors. (*B*) Fraction of whole blood TWN edges replicating in an independent RNA-seq data set (DGN) (Battle et al. 2014; Mostafavi et al. 2014).

On average, each TWN contained 60,697 edges, with 24,527 edges between TE nodes, 18,539 edges between IR nodes, and 17,631 edges connecting TE and IR nodes (Fig. 2A). We found many nodes with large numbers of neighbors (*hub nodes*), as expected in biological and other scale-free networks (Barabasi and Oltvai 2004). Based on a threshold of 10 or more neighbors, TWNs had a mean of 1853 “TE-TE” hub genes (total expression nodes connected to many total expression neighbors) and 325 “TE-IR” hub genes (total expression nodes connected to many isoform ratio neighbors) across tissues (Fig. 2A). Hubs with numerous total expression neighbors were more common, but hubs with isoform ratio neighbors were also found in every tissue (Fig. 2A).

Reconstructing co-expression networks requires estimation of a large number of parameters (in our case, over 2 × 10^8^) despite a small number of samples (≤430); robustness and replicability of network edges are thus important considerations. While there are not other large-scale RNA-seq data sets for most GTEx tissue types, we replicated relationships identified by our GTEx whole blood TWN using an independent whole blood RNA-seq data set on 922 individuals of European ancestry from the Depression Genes and Networks study (DGN) (Battle et al. 2014; Mostafavi et al. 2014). First, we tested whether TE and IR nodes connected by an edge in the GTEx whole blood TWN were also correlated in DGN. For all edge types, we found that a higher fraction of node pairs connected by an edge in the GTEx TWN were correlated in DGN compared to nodes from random networks (84.7% versus 45.6%, 31.9% versus 5.9%, and 20.9% versus 2.6% for TE-TE, TE-IR, and IR-IR edges, respectively; false discovery rate (FDR) ≤ 0.05) (Fig. 2B). Next, we reconstructed a TWN from DGN data over genes and isoforms common to both data sets. All pairs of nodes connected directly or indirectly in the GTEx whole blood TWN had significantly shorter network path distance in the DGN network compared to the distance in the same network with the node labels shuffled (Wilcoxon rank-sum test, *P* ≤ 2.2 × 10^−16^) (Supplemental Fig. S4). This provides replication in an independent data set for the same tissue, despite different alignment and isoform quantification pipelines between the two data sets.

TWN relationships were also replicated by substituting a second gene regulatory network reconstruction method, ARACNE (Margolin et al. 2006), in place of the graphical lasso, using the same overall framework and quantification of TE and IR levels in the GTEx data. ARACNE captured 37.73% of the graphical lasso edges on average, compared to the expected proportion (0.15%) of edges captured at random (Supplemental Fig. S5), showing that the TWN signal is robust to choice of network estimation method.

### TWN hubs are enriched for regulators of splicing

We used the sixteen TWNs to characterize the regulation of relative isoform abundance in each GTEx tissue. Here, we focused on evaluation of network hubs. Hub genes tend to be essential in biological mechanisms and, in a co-expression network, are likely to have regulatory functions (Jeong et al. 2001; Barabasi and Oltvai 2004; Albert 2005). Unlike traditional networks, TWNs have four categories of hub genes that likely reflect different regulatory functions (Fig. 1C). For instance, a hub arising from a total expression node connected to a large number of isoform ratio neighbors (TE-IR hub) may reflect a gene important in regulation of alternative splicing. We identified the top hub nodes by *degree centrality*—the number of edges per node—for all node categories in each of the 16 tissues (Supplemental Table S2; Supplemental Data S2). To avoid bias due to different numbers of isoforms per gene, we measured degree centrality of a node by the number of unique genes among neighboring nodes in each category (Methods).

**Figure 3.**
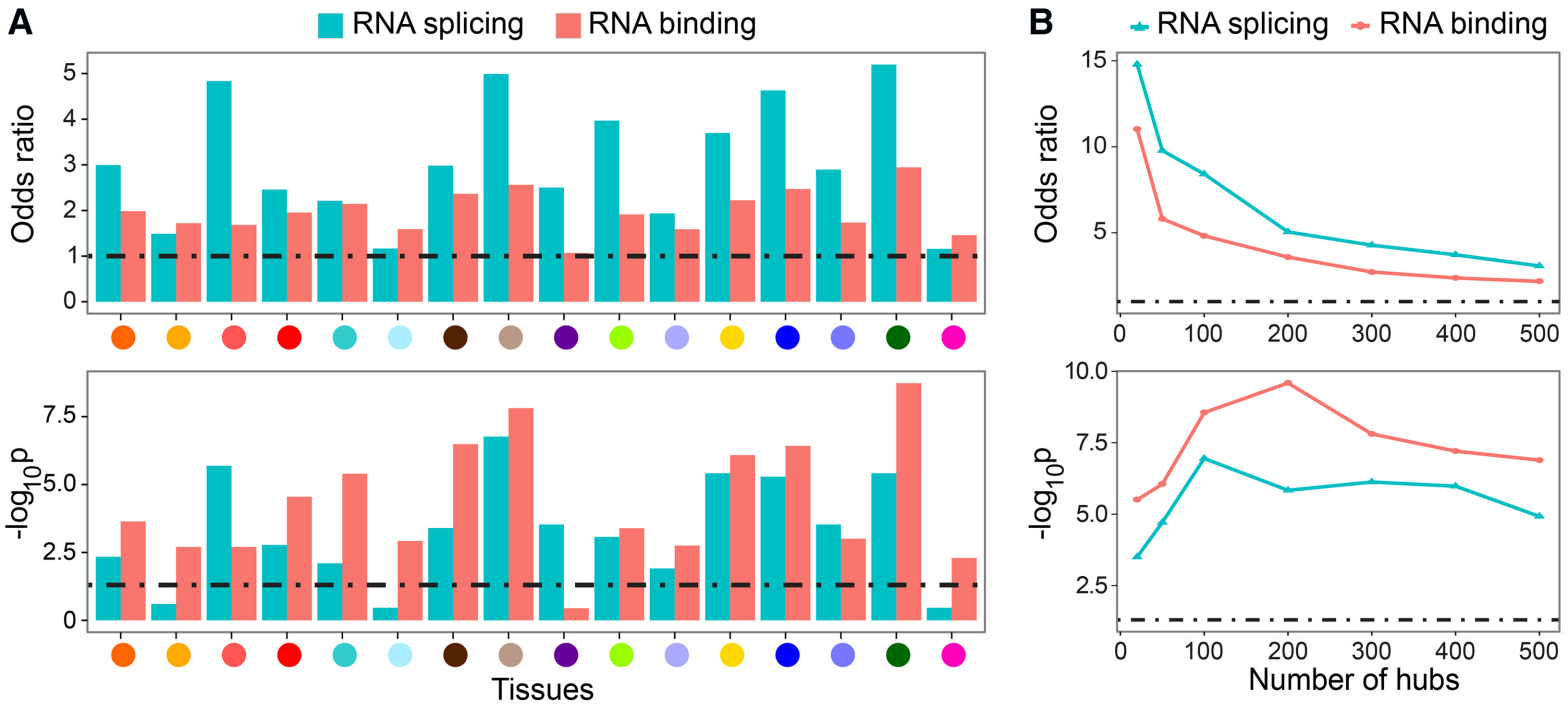
Enrichment of candidate splicing regulators among TWN hubs. (*A*) In each TWN, the odds ratio and *P*-value of enrichment among the top 500 TE-IR hub genes for GO annotations reflect RNA binding and RNA splicing. (*B*) Among consensus TE-IR hubs across all tissues, enrichment for GO annotations reflects RNA binding and RNA splicing functions.

We investigated whether hub nodes with many IR neighbors were likely to be regulators of alternative splicing. For each tissue, we evaluated the top TE-IR hubs for enrichment of Gene Ontology (GO) terms related to RNA splicing and observed a significant abundance of known RNA splicing genes (annotated with GO:0008380) among the top TE-IR hubs. Indeed, 13 of 16 tissues (81.25%) showed significant enrichment of RNA splicing genes in the top 500 TE-IR hubs (significance assessed at Benjamini-Hochberg [BH]-corrected *P* ≤ 0.05; median across all tissues *P* ≤ 6.22 × 10^−4^, Fisher's exact test) (Supplemental Methods), and every tissue had a larger than unit odds ratio of RNA splicing genes among the top hubs (Fig. 3A). Enrichment was robust to choice of hub degree threshold (Supplemental Fig. S6). Next, we tested for enrichment of RNA binding proteins, many of which are known to be important regulators of RNA splicing and processing (Wang and Burge 2008; Chen and Manley 2009; Witten and Ule 2011). We found that RNA binding genes (annotated with GO:0003723) were also significantly enriched, at BH-corrected *P* ≤ 0.05, among the top TE-IR hubs of every tissue except heart– left ventricle (median *P* ≤ 3.17 × 10^−4^) (Fig. 3A). Across all GO terms, *splicing*, *RNA binding*, and *RNA processing* were consistently among the most enriched for TE-IR hubs across tissues (Supplemental Tables S3, S4). The replication network estimated from the DGN data also indicated relevant enrichment among TE-IR hubs (*RNA splicing*: *P* ≤ 1.07 × 10^−5^, odds ratio 2.72; *RNA binding*: *P* ≤ 2.5 × 10^−11^, odds ratio 2.37).

Many regulatory relationships are shared between tissues, and assessing hubs across all tissues jointly may improve robustness (Ballouz et al. 2015). Therefore, we identified TE-IR hubs shared across tissues (Table 1; Supplemental Data S3) using rank-product (Zhong et al. 2014). We first ranked hub genes according to the number of neighbors in each network. We then aggregated the ranks of those genes across all networks by computing the product of these ranks and sorted genes to find the top TE-IR hubs (those with the largest number of neighbors in the most tissues) (Methods). We observed much stronger enrichment for RNA splicing and RNA binding in the joint analysis than in individual tissues (Fig. 3B).

**Table 1.**
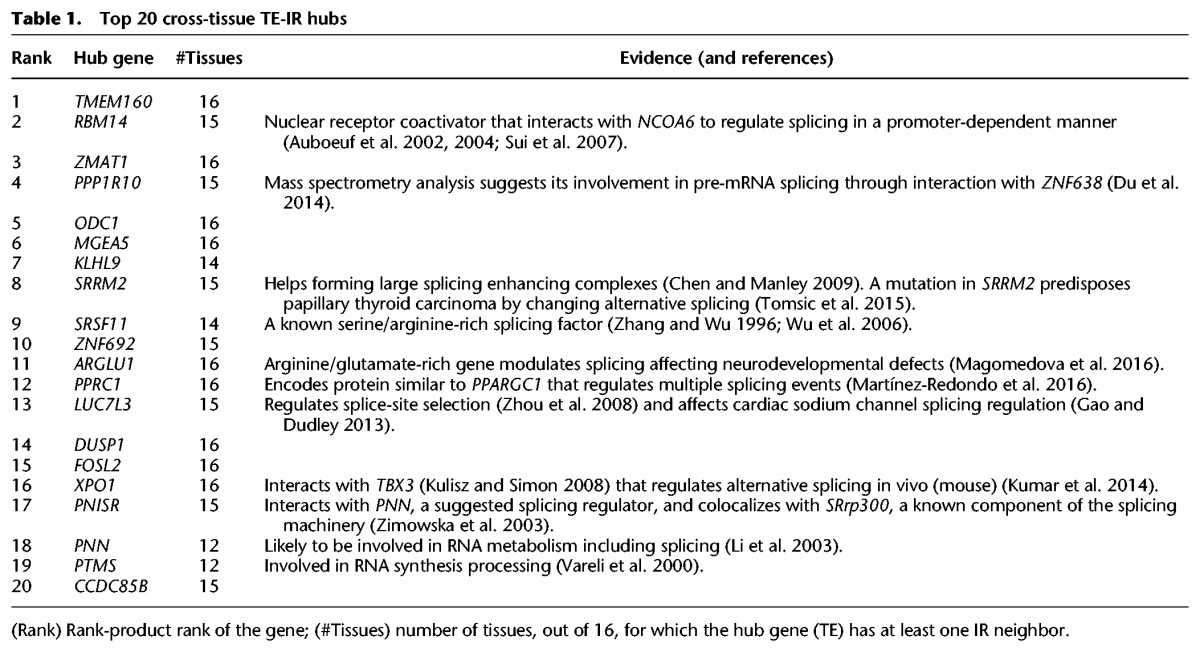
Top 20 cross-tissue TE-IR hubs

Many of the top ranked TE-IR hubs shared across tissues are known to regulate splicing. *RBM14* (rank 2), an RNA binding gene also known as *COAA*, interacts with a transcription regulator *TARBP2* to regulate splicing in a promoter-dependent manner (Auboeuf et al. 2002, 2004). Another RNA binding gene *PPP1R10* (rank 4) has been implicated in pre-mRNA splicing using mass spectrometry analysis (Du et al. 2014). *SRRM2* (rank 8) and *SRSF11* (rank 9) are also known splicing regulators (Zhang and Wu 1996; Blencowe et al. 2000; Wu et al. 2006; Chen and Manley 2009). For 11 of the top 20 cross-tissue TE-IR hubs, we found previous work supporting a role in the regulation of splicing (Table 1). These results suggest that TWN hubs are informative of splicing regulation, and uncharacterized TE-IR hub genes in a TWN are good candidates for regulatory effects on isoform abundance.

### Coregulation of expression and isoform ratios reflects biological pathways

**Figure 4.**
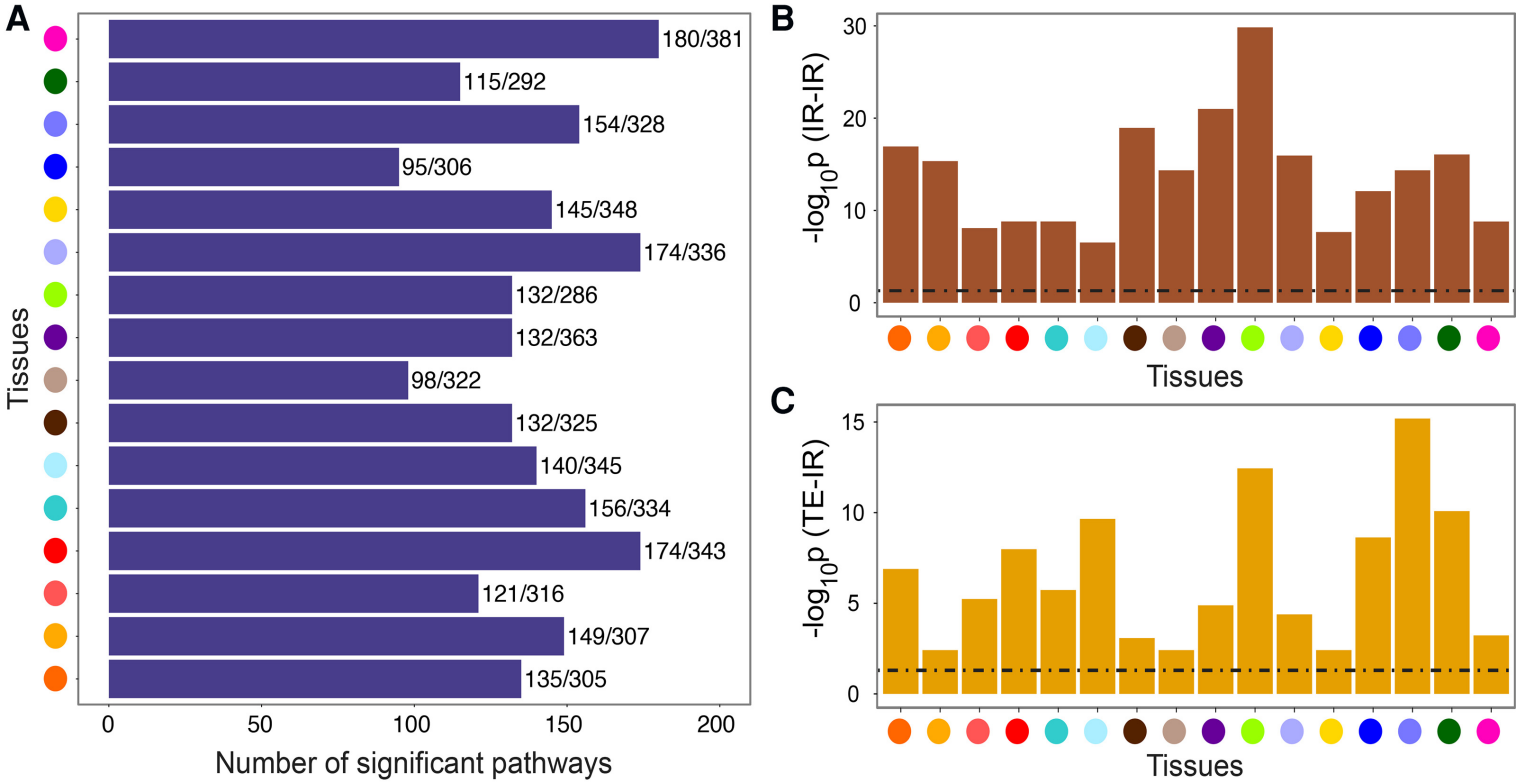
Pathway enrichment in TWNs. Tissue colors are matched with tissue names in Figure 2. (*A*) Per-tissue, the number of Reactome pathways enriched among connected components/total number of tested pathways for that tissue, considering only TE nodes. (*B*) Enrichment for shared Reactome pathway annotation among gene pairs connected by an edge between two TE nodes. (*C*) Enrichment for shared Reactome pathway annotation among gene pairs connected by an edge between a TE and an IR node.

Genes with similar function or that participate in the same pathway often have correlated patterns of gene expression (Prieto et al. 2008; Roider et al. 2009; Khatri et al. 2012; Hormozdiari et al. 2015). In the GTEx TWNs, we observed enrichment of edges between transcription factors and known target genes (Supplemental Methods; Supplemental Fig. S7). We also observed greater enrichment of closely connected genes for Reactome (Fabregat et al. 2016) and KEGG (Kanehisa et al. 2016) pathways as compared with permuted networks (95−180 Reactome and 39–82 KEGG pathways enriched per tissue at Bonferroni corrected *P* ≤ 0.05; Wilcoxon rank-sum test) (Fig. 4A; Supplemental Fig. S8; Supplemental Methods).

Patterns of correlation among relative isoform abundances are not well studied, and it has not been established whether the regulation of splicing is coordinated across functionally related genes. Initial studies have identified such correlation in particular tissues (Iancu et al. 2015) and specific processes (Dai et al. 2012). To extend this, we evaluated each TWN for enrichment of edges between functionally related genes. For all 16 tissues, the TWNs demonstrated significant abundance of edges between isoform ratios of two distinct genes that participate in the same Reactome pathway (Fisher's exact test; all tissues significant at BH-corrected *P* ≤ 0.05; median *P* ≤ 10^−14^) (Fig. 4B). Similarly, TE-IR edges were enriched for pairs of genes that participate in the same pathway (median *P* ≤ 10^−5^) (Fig. 4C). As expected, we also observed shared-pathway enrichment for nodes connected by TE-TE edges (Supplemental Fig. S9). The patterns of functional enrichment were stronger among pairs of TE nodes, which may be due to more accurate quantification of total expression versus isoform ratios from RNA-seq data, functional annotations derived from gene expression studies, or tighter coregulation of transcription than splicing among functionally related genes. Leveraging the coregulation of splicing among functionally related genes, TWNs can be used to predict gene function (Warde-Farley et al. 2010) based on a more comprehensive understanding of coregulation, including regulation of splicing.

### Comparison between TWNs reveals per-tissue hub genes

We evaluated the overall similarity of the TWNs between tissues. We tested concordance of hubs between each pair of tissues using Kendall's rank correlation computed over genes ordered by degree centrality (Supplemental Fig. S10). We observed greater than random levels of similarity between most tissues for all hub types (Kendall's rank correlation test; median *P* ≤ 1.0 × 10^−5^ for each hub type), and functionally related tissues showed greater levels of similarity. For example, the two skin tissues were grouped together for each hub type and were found to be similar to esophagus–mucosa, which contains primarily epithelial tissue (Squier and Kremer 2001). Skeletal muscle and heart–left ventricle grouped together, and breast–mammary was similar to the two adipose tissues, reflecting shared adipose cell type composition. While these results may be influenced by overlapping donors, they provide evidence that splicing is more similar in tissues with shared cell type compositions (Qian et al. 2005; Ong and Corces 2011; The GTEx Consortium 2017).

To identify candidate tissue-specific regulatory genes, we evaluated TE-IR hubs that had a high rank in related tissues but a low rank among unrelated tissues (Methods; Supplemental Table S5; Supplemental Data S4). Several of the top ranked tissue-specific hubs were genes with evidence of known tissue-specific function or relevance. In the tissue group including breast–mammary and the two adipose tissues, the top tissue-specific TE-IR hub was *TTC36*, a gene highly expressed in breast cancer (Liu et al. 2008). The second ranked hub gene for the tissue group including skeletal muscle and heart–left ventricle was *LMOD2*, which was observed to be abundantly expressed in both tissues and has been reported to regulate the thin filament length in muscles affecting cardiomyopathy in mice (Pappas et al. 2015; Li et al. 2016b).

We evaluated the tissue-specificity of our identified hub genes. To do this, we computed the fraction of top 100 TWN hubs of each tissue that did not appear in the list of top 500 TWN-hubs of any other tissue (Supplemental Methods). We found that 8%–43%, 11%–39%, 0%–24%, and 0%–20% of our top 100 TE-TE, TE-IR, IR-TE, and IR-IR hubs, respectively, were uniquely identified in a single tissue (Supplemental Fig. S11). TE hubs (TE-TE and TE-IR hubs) were more likely to be tissue-specific than matched IR hubs (IR-TE and IR-IR hubs; one-sided Wilcoxon signed rank test, *P* ≤ 4.13 × 10^−7^). Tissue-specific hub proportions were not significantly different between TE-TE and TE-IR hubs (two-sided Wilcoxon signed rank test, *P* ≤ 0.52). Many of the hub genes were differentially expressed across tissues (Supplemental Methods; Supplemental Table S6).

An average of 69.87% of tissue-specific TWN edges connected nodes where at least one node was differentially expressed between the tissue of interest and all other tissues (Supplemental Table S7). For 6.9% of tissue-specific edges, at least one node was not included in a TWN for any other tissue because of low expression or other filters. However, for the remaining 23.22% of tissue-specific edges, both nodes were expressed in other tissues and included in other networks, so the tissue-specificity of edges is not exclusively due to expression levels.

### Tissue-Specific Networks identify gene co-expression patterns unique to tissues

A per-tissue TWN contains both shared and tissue-specific co-expression relationships between genes, without making any distinction between them, reflecting the full gene network in each tissue. To directly assess the tissue-specificity of co-expression relationships, we built Tissue-Specific Networks (TSNs) by considering all GTEx samples across 50 tissues simultaneously, decomposing the contributions to gene expression level variation into signals shared across tissues and those specific to single tissues. To do this, we applied a Bayesian biclustering framework, BicMix (Gao et al. 2016), and reconstructed tissue-specific networks (Methods; Supplemental Figs. S12, S13). BicMix incorporates a prior distribution that encourages sparsity in the solution in order to differentiate between gene co-expression relationships specific to a single tissue and those shared across tissues, simultaneously controlling for batch effects, population structure, and shared individual effects across tissues (Gao et al. 2016). Applied to over 7000 RNA-seq samples with more comprehensive sampling of heterogeneous tissues types, this approach is able to isolate co-expression signals unique to single tissues and to reconstruct precise and interpretable TSNs.

We identified TSNs for 26 GTEx tissues. Here, we limited network nodes to total gene expression for simplicity. Across the 26 TSNs, the mean number of nodes (considering only genes with tissue-specific edges) was 24, and the average number of edges was 107 (Supplemental Fig. S14; Supplemental Table S8). As expected, TSNs contained a small subset of edges from full per-tissue TWNs, representing the co-expression components that are tissue-specific rather than shared. However, the signal in the TSNs is still reflected within their matched TWNs for the eight tissues where we reconstructed both types of networks based on multiple metrics of concordance (Supplemental Figs. S15–S17).

Additionally, we built 10 TSNs for groups of similar tissues (see Supplemental Methods), including a group combining all brain tissues, to capture gene relationships common within each group but unique compared with all other tissues. Most tissues showed expression patterns close to at least one other assayed tissue (Supplemental Fig. S18), leading to a depletion of tissue-specific effects and motivating evaluation of similar tissues together. On average, tissue group networks contained 2018 edges and 93 nodes. However, this was skewed by the brain network, which contained 18,854 edges connecting 648 nodes. Excluding the brain network, we found 147 edges and 31 nodes, on average, across the other nine tissue group networks.

### Functional analysis of TSNs

**Figure 5.**
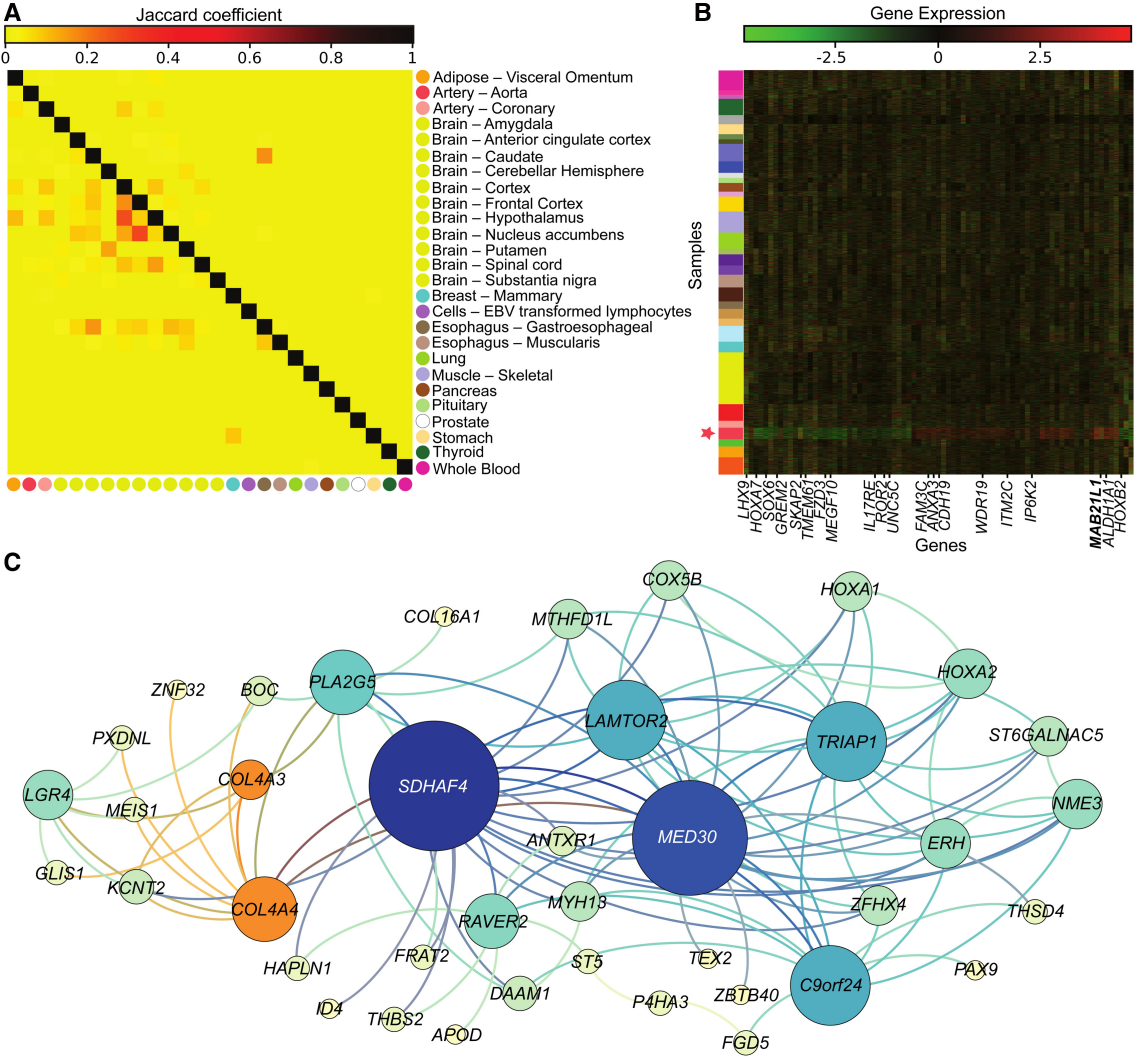
Cross-tissue comparison of TSN results. (*A*) Jaccard coefficient quantified on shared edges (*upper* triangular) and shared nodes (*lower* triangular) across pairs of TSNs. (*B*) Gene expression levels, removing factors from BicMix not included in the network, for the genes identified in the TSN for artery–aorta. The *y*-axis is ordered by similarity to artery–aorta, with a star by the samples from artery–aorta. The colors on the *y*-axis correspond to the GTEx tissue legend above. The *x*-axis is ordered by expression similarity (i.e., hierarchical clustering), and hub genes are labeled, with the large hub denoted in bold. (*C*) TSN for artery–coronary. Node size reflects betweenness centrality of the nodes. Orange nodes reflect replication in the BioCarta *acute myocardial infarction* (AMI) pathway; orange edges show the neighbors of the AMI pathway nodes.

We investigated the functional properties of each TSN. First, we measured sharing of network components between the 26 distinct TSNs. We found minimal sharing of network nodes and even less sharing of network edges among all pairs of tissues (Jaccard coefficient) (Fig. 5A). This was expected as a result of BicMix's strong control over confounding effects and co-expression shared across tissues. Tissue pairs that appeared to share network genes predominantly included brain tissues.

We studied the genes within each TSN for biological relevance, evaluating each network for enrichment using all GO biological process terms. We found that, for 21 out of 26 TSNs, significantly enriched pathways included tissue-relevant GO biological process terms (Fisher's exact test, BH-corrected *P* ≤ 0.05) (Supplemental Table S9). We also confirmed enrichment of known tissue-specific genes using a previously defined list of GO terms (Ashburner et al. 2000) indicative of tissue-specific transcription factor functions available for 11 tissues (Fisher's exact test) (Supplemental Fig. S19; Pierson et al. 2015). We found four of the 11 TSNs nominally enriched for genes with specificity in the matched tissue, namely artery–coronary (BH-corrected *P* ≤ 0.23), EBV transformed lymphocytes (with blood, BH-corrected *P* ≤ 0.09), skeletal muscle (BH-corrected *P* ≤ 0.13), and stomach (BH-corrected *P* ≤ 0.15). Perhaps due to cell type heterogeneity and shared cell types, significant cross-tissue enrichments were observed in a small number of tissues. For example, in the artery–aorta TSN, pituitary genes were significantly enriched (BH-corrected *P* ≤ 0.0049).

Next, we evaluated the hub genes in each TSN, considering three thresholds of centrality: ≥5 edges (“small hubs”), ≥10 edges (“hubs”), and ≥50 edges (“large hubs”). Hubs were not enriched overall for cross-tissue transcription factors (hypergeometric test across all TSNs, *P* ≤ 0.84; small and large hubs showed similar results), or for cross-tissue and tissue-specific TFs (hypergeometric test across all TSNs, *P* ≤ 0.90; small and large hubs showed similar results). This may be because TFs that are not tissue-specific and that affect many genes downstream will be captured by BicMix in dense, multi-tissue factors; because these factors will not be used to construct the networks, such broad TF signals will be systematically removed. Similar results have been observed in expression quantitative trait loci (eQTL) analysis, where *cis*-eQTL target genes are depleted for TFs (Battle et al. 2014) and *trans*-eQTL variants are not enriched as targeting TFs in *cis* (The GTEx Consortium 2017). This could arise due to the tightly controlled regulation of the expression of TFs themselves (Battle et al. 2014) but could also be the result of removing latent factors correlated with TF expression (Weiser et al. 2014; The GTEx Consortium 2017), including broad biological effects and confounders. However, hubs in several networks included genes known to play a role in tissue-specific function and disease. Specifically, we found that the single large hub in brain–caudate, *MAGOH*, which is a part of the exon junction complex that binds RNA, has been found to regulate brain size in mice through its role in neural stem cell division (Silver et al. 2010). The single large hub for artery–aorta, *MAB21L1*, has been shown to be an essential gene for embryonic heart and liver development in mice by regulating cell proliferation of proepicardial cells (Saito et al. 2012).

Additionally, we measured enrichment of known pathways in the TSNs. While we did not observe enrichment across all tissues, we found that the EBV transformed lymphocyte TSN was significantly enriched for the *hematopoietic cell lineage* KEGG pathway (Fisher's exact test, BH-adjusted *P* ≤ 0.05); a hematopoietic stem cell is the developmental precursor of leukocytes. The EBV transformed lymphocyte TSN also had significant enrichment in the BioCarta *IL-17 signaling* and *T cytotoxic cell surface molecules* pathways (Fisher's exact test, BH-adjusted *P* ≤ 1.50 × 10^−4^). *IL-17* is a cytokine produced in T-cells that is involved in inflammation. Although not significant after multiple testing correction, artery–coronary showed nominal enrichment in four tissue-relevant pathways (uncorrected *P* ≤ 0.016 for all): the *ACE2* pathway, which regulates heart function; the *acute myocardial infarction* (AMI) pathway; the *intrinsic prothrombin activation* pathway, which is involved in one phase of blood coagulation; and the *platelet amyloid precursor protein* (APP) pathway, which includes genes involved in anti-coagulation functions. In the brain group TSN, we observed significantly shorter distances between the genes in each of the KEGG *Parkinson's*, *Alzheimer's*, and *Huntington's* pathways compared to a randomly permuted network, reflecting three canonically brain-specific diseases (Wilcoxon rank-sum test, BH-corrected *P* ≤ 0.075).

### Integration of networks with regulatory genetic variants

Both TWNs and TSNs were estimated using gene expression data alone. However, the GTEx v6 data also include genotype information for each donor. We intersected the edges detected by our networks with expression quantitative trait locus (eQTL) association statistics to replicate specific network edges through evidence of conditional associations with genetic variants across those edges and to increase power to detect long range (*trans*) effects of genetic variation on gene expression.

**Figure 6.**
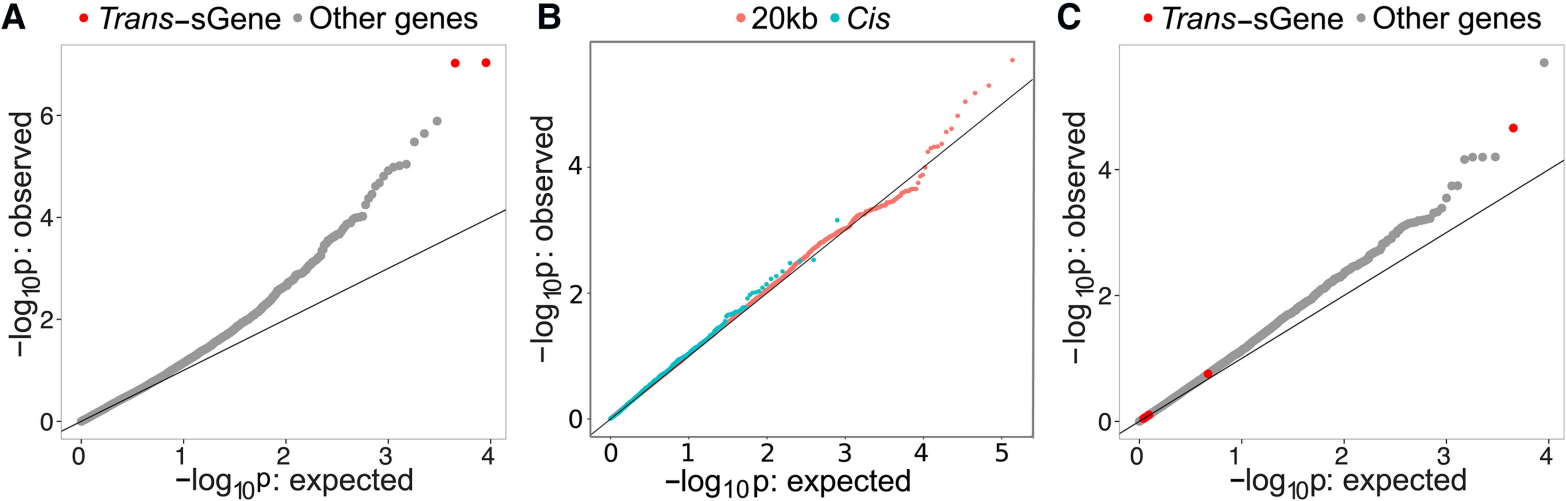
Association of local genetic variants with distant network neighbors. (*A*) Enrichment of association between rs113305055, a genetic variant near a cross-tissue TWN hub *TMEM160*, with all isoform ratios genome-wide in artery–tibial. (*B*) Enrichment of associations between local genetic variants (either the top *cis*-eVariant or any variant within 20 kb) of each gene, and network neighbors in the TSNs. (*C*) Enrichment of association between rs115419420, a genetic variant local to *CRELD1*, with all isoform ratios in skeletal muscle.

First, we demonstrated that, for both TWNs and TSNs, there was enrichment for associations between the top *cis*-eVariant (the variant with lowest *P*-value per gene with a significant *cis*-eQTL) for each gene and the expression level or isoform ratio of its network neighbors based on QTL mapping in the corresponding tissue (Fig. 6). This provides evidence of a causal relationship between connected genes. For TWNs, evaluating TE nodes with an IR neighbor, we found evidence for 61 *trans* (i.e., inter-chromosomal) associations and 86 intra-chromosomal associations tested between a *cis*-eVariant for the TE gene and the IR of the neighboring node (FDR ≤ 0.05). Our top two associations were between two variants, rs113305055 in artery–tibial and rs59153288 in breast–mammary (both near *TMEM160*), with isoform ratios of *CST3* (*P* ≤ 9.3 × 10^−8^, and *P* ≤ 4.0 × 10^−7^, respectively). *TMEM160* is the top cross-tissue hub in our TWNs with many IR neighbors (Table 1). Thus, we tested for association of these variants with all isoform ratios genome-wide and observed a substantial enrichment of low *P*-values in numerous tissues (Fig. 6A; Supplemental Fig. S20). In the TSNs, we identified five *cis*-eVariants across five tissues associated with six different *trans*-eGenes through six unique *cis*-eGene targets, one of which was intra-chromosomal (FDR ≤ 0.2) (Supplemental Table S10). We also observed enrichment for low *P*-values over the tests corresponding to each network edge (Fig. 6B).

We also performed a restricted test to identify novel *trans*-QTLs, without relying on the *cis*-eQTL signal from the same data, to avoid discoveries driven by potentially spurious correlations among expression levels. From the TWNs, we sought to identify *trans*-splicing QTLs (sQTLs) based on TE-IR hub genes, using the top 500 hubs by degree centrality. We tested every single nucleotide polymorphism (SNP) within 20 kb of the TE hub-gene's transcription start site (TSS) for association with isoform ratios of each neighbor in the TWN. Using this approach, we identified 58 *trans*-sQTLs corresponding to six unique genes (sGenes) at FDR ≤ 0.2 (Table 2; Supplemental Data S5). For example, we identified a *trans*-sQTL association in skeletal muscle between rs115419420 and *CARNS1* (*P* ≤ 2.18 × 10^−5^) that is supported by a *cis* association with the TE-IR hub *CRELD1*. This variant also showed enrichment for low *P*-values with numerous isoform ratios genome-wide (Fig. 6C). In the TSNs, we identified 14 *trans*-eQTLs using variants within 20 kb of each gene and testing for association with the neighbors of those genes in the gene expression data of the same tissue (FDR ≤ 0.2) (Supplemental Table S11). All of these associations were inter-chromosomal. Overall, we saw an enrichment of *P*-values for association between genetic variants local to a gene and the gene's neighbors in each network (Fig. 6B).

**Table 2.**
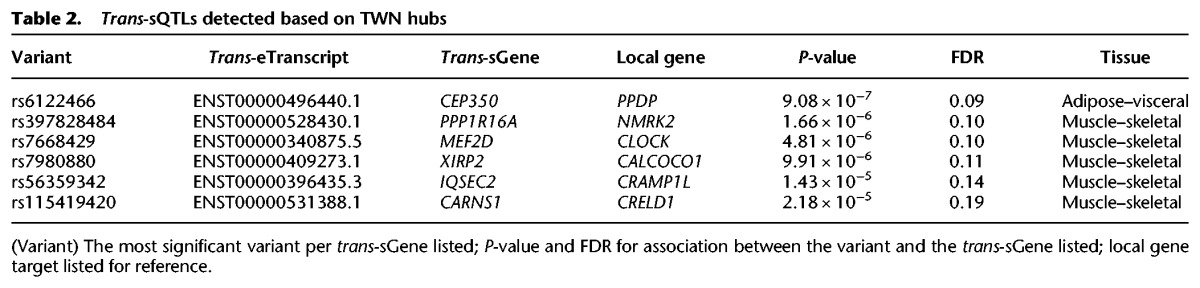
*Trans*-sQTLs detected based on TWN hubs

## Discussion

We reconstructed co-expression networks that capture novel regulatory relationships in diverse human tissues using large-scale RNA-seq data from the GTEx project. First, we specified an approach for integrating both total expression and relative isoform ratios in a single sparse Transcriptome-Wide Network. Splicing is a critical process in a number of tissue- and disease-specific processes and pathways (Hutton et al. 1998; D'Souza et al. 1999; Glatz et al. 2006; Ghigna et al. 2008), but, critically, isoform ratios have not been included in co-expression network analysis to allow the study of splicing regulation. We estimated TWNs from 16 tissues and demonstrated that hubs in TWNs are strongly enriched for genes involved in RNA binding and RNA splicing. We found that, across tissues, the top hub genes with isoform ratio neighbors included many genes with known impact on splicing such as *RBM14*, a hub in all 16 tissues with TWNs. We identified a number of novel shared and tissue-specific candidate regulators of alternative splicing. While TWNs demonstrated clear enrichment for capturing desired regulatory relationships, care should be taken in interpreting individual edges and network relationships, as false positives may still arise due to confounding technical and biological factors and from estimating large networks based on limited sample sizes. However, as more large-scale RNA-seq studies and better transcript quantification tools become available, TWNs will continue to be a useful and extensible framework for analyzing diverse types of regulatory relationships in disease, longitudinal, and context-specific studies.

Next, we estimated Tissue-Specific Networks for 26 single tissues and across 10 tissue groups; these networks represent co-expression relationships unique to individual tissues and sets of closely related tissues. Distinguishing between shared and tissue-specific structure across single tissue co-expression networks is challenging but essential for understanding tissue-specific regulatory processes in disease. From these TSNs, we identified hub genes involved in the tissue-specific regulation of transcription, such as *MAGOH* in the brain–caudate-specific network and *MAB21L1* in the artery–aorta-specific network, both of which are essential for the development of their specific organs. A majority of networks were enriched with genes annotated to tissue-relevant GO terms. We used these networks to quantify shared relationships across tissues and found minimal sharing of relationships across these 26 tissues. Finally, we replicated edges in our networks by integrating genetic variation, and we identified 20 novel *trans*-QTLs affecting both expression and splicing. Together, our results provide the most comprehensive map of gene regulation, splicing, and co-expression in the largest set of tissues available to date. These networks will provide a basis for interpreting the transcriptome-wide effects of genetic variation, differential expression, and splicing in complex disease, and the impact of diverse regulatory genes across human tissues.

## Methods

### Data from the GTEx project

We collected RNA-seq and genotyping data from the Genotype-Tissue Expression (GTEx) consortium v6 data (The GTEx Consortium 2017). GTEx obtained tissue samples (averaging about 28 per individual) from postmortem donors between ages 21 and 70, BMI 18.5 to 35, and not under exclusionary medical criteria such as whole blood transfusion within 24 h or infection with HIV. Seventy-six-base pair (bp) pair-ended RNA-seq was performed with Illumina HiSeq 2000 following the TrueSeq RNA protocol. After quality control, we aligned the RNA-seq reads using the STAR aligner in 2-pass mode (Dobin et al. 2013). We then performed transcript and gene quantification using RSEM v1.2.20 (Li and Dewey 2011). See The GTEx Consortium (2017) and Supplemental Methods for details. We used RNA-seq data across 50 tissues in 449 individuals.

Approximately 1.9 million SNPs were genotyped using whole blood samples with Illumina HumanOmni 2.5 M and 5 M BeadChips (see Supplemental Methods). Additional variants were imputed using IMPUTE2 (Howie et al. 2009). The genotypes were filtered for MAF ≥ 0.05, leaving approximately 6 million variants.

### Preprocessing for per-tissue TWNs

We considered only protein-coding genes on the autosomes and Chromosome X to construct TWNs in all tissues. We used genes and isoforms with at least 10 samples with ≥1 TPM and ≥6 reads. We filtered out genes where the Ensembl gene ID did not uniquely map to a single HGNC gene symbol. Isoform ratio was computed by using annotated isoforms in GENCODE V19 annotation, and undefined ratios (0/0, when none of the isoforms were expressed) were imputed from the mean ratio per isoform across individuals. Each gene's least abundant isoform was excluded to avoid linear dependency between isoform ratio values. We log-transformed the total expression data and standardized both total expression levels and isoform ratios. To correct hidden confounding factors, we applied the hidden covariates with prior (HCP) method (Mostafavi et al. 2013), whose parameters were selected based on an external signal relevant to regulatory relationships. Namely, we selected parameters that produced maximal replication of an independent set of *trans*-eQTLs from meta-analysis of a large collection of independent whole blood studies (Westra et al. 2013). For both total expression levels and isoform ratios of genes in all tissues, the best HCP parameters (*k* = 10, λ = 1, σ_1_ = 5, σ_2_ = 1), which consistently reproduced a largest subset of the gold-standard *trans*-eQTLs in GTEx whole blood samples even when subsetting the number of samples, were used for correcting data. Finally, quantile-normalization to a standard normal distribution was applied per gene.

To avoid spurious associations due to mismapped reads, we filtered out genes with mappability < 0.97 and their isoforms (see Supplemental Methods; The GTEx Consortium 2017). We also filtered out isoforms of a gene if the mean IR of the most dominant isoform was ≥0.95. In each tissue, we further reduced the number of features to 6000 genes and 9000 isoforms for computational tractability based on expression level and isoform variability (see Supplemental Methods). On average, the final selected isoforms for each tissue belong to 4357 unique genes (Supplemental Table S12).

### Per-tissue Transcriptome-Wide Networks

We built per-tissue Transcriptome-Wide Networks using a scalable graphical lasso (Hsieh et al. 2011). We estimated a sparse precision matrix (Θ) by optimizing the following objective with Λ specifying different penalties for different types of edges:

(1) Θ^=argminΘ−log⁡detΘ+tr(SΘ)+∥Λ∘Θ∥1,

where the entry in the *r*th row and *c*th column of Λ was

(2) $$\begin{array}{c} \Lambda_{rc}=\left\{ \begin{array}{cc} \lambda_{d}\; & \;\text{if}\;r=c\;\\ \lambda_{s}\; & \;\text{if}\;r\neq c\;\text{and}\;\mathrm{gene}(r)=\mathrm{gene}(c)\;\\ \lambda_{tt}\; & \;\text{if}\;\mathrm{gene}(r)\neq \mathrm{gene}(c)\;\text{and}\;\mathrm{type}(r)=\mathrm{type}(c)=\text{'TE'}\;\\ \lambda_{ti}\; & \;\text{if}\;\mathrm{gene}(r)\neq \mathrm{gene}(c)\;\text{and}\;\{\mathrm{type}(r),\mathrm{type}(c)\}=\{\text{'TE'},\text{'IR'}\}\;\\ \lambda_{ii}\; & \;\text{if}\;\mathrm{gene}(r)\neq \mathrm{gene}(c)\;\text{and}\;\mathrm{type}(r)=\mathrm{type}(c)=\text{'IR'}.\; \end{array}\right. \end{array}$$

Here, *gene*(*k*) denotes the gene that the *k*th feature belongs to; *type*(*k*) denotes whether or not the *k*th feature represents total expression (“TE”) or isoform ratio (“IR”).

We did not penalize diagonal entries (λ_*d*_ = 0), and we put in a small nonzero penalty for edges between distinct features belonging to the same gene (λ_*s*_ = 0.05), such as distinct isoforms of the same gene. We selected the other penalties (λ_*tt*_, λ_*ti*_, λ_*ii*_) such that the network had a scale-free topology with a reasonable number of edges. The empirical pairwise correlation distributions for different types of edges were different: Correlations between two total expression nodes were generally much higher than correlations between two isoform ratio nodes or between a total expression node and an isoform ratio node (Supplemental Fig. S2), while the latter two distributions were apparently similar. We tried all (λ_*tt*_, λ_*ti*_, λ_*ii*_) combinations where λ_*tt*_∈{0.3,0.35,0.4,0.45,0.5}, λ_*ti*_∈{0.25,0.3,0.35,0.4}, and λ_*ti*_ = λ_*ii*_. We measured the scale-free property by the square of correlation (*R*^2^) between log(*p*(*d*)) and log(*d*), where *d* is an integer and *p*(*d*) represents the fraction of nodes in the network with *d* neighbors (Zhang and Horvath 2005). We selected penalty parameters so that *R*^2^ ≈ 0.85 and there were at least 5000 edges of each type. Selected parameters for each tissue are shown in Supplemental Table S1. Each nonzero element in Θ_*rc*_ in the precision matrix with selected penalty parameters represents an edge between the *r*th and *c*th features in our network.

We excluded some edges from our networks for quality purposes and interpretability. Specifically, we excluded edges between nodes belonging to the same gene for downstream analysis. Then, we aligned every 75-mer in exonic regions and 36-mers in UTRs of every gene with mappability < 1.0 to the reference human genome (hg19) using Bowtie (v 1.1.2) (Langmead et al. 2009). If any of the alignments started within an exon or an UTR of another gene, then these two genes were considered “cross-mappable,” and we excluded edges between cross-mappable genes. We also excluded edges between genes with overlapping positions in the reference genome to avoid mapping artifacts.

### Replication of whole blood TWN

We replicated our network edges with GTEx whole blood tissue in an independent RNA-seq data set: Depression Genes and Networks (Battle et al. 2014; Mostafavi et al. 2014). DGN includes quantifications of 15,231 genes and 12,080 isoforms from whole blood in 922 samples, out of which 5609 genes and 1464 isoforms were uniquely mapped to the set of genes and isoforms used in GTEx whole blood TWN reconstruction. First, to check if the genes and isoforms directly connected in the GTEx whole blood network were supported by correlation in the DGN data set, we computed the fraction of significantly correlated (Spearman correlation, FDR ≤ 0.05) TE-TE/TE-IR/IR-IR pairs in DGN. We then compared these fractions with those in random pairs generated by permuting genes/isoforms labels in the TWN. Next, to verify if our method could reproduce relationships in the GTEx whole blood network for DGN data, we tested if node pairs connected directly or indirectly in the GTEx whole blood network had a shorter distance (path length) between them in the DGN network compared to the same network with the node labels shuffled. We performed a one-sided Wilcoxon rank-sum test between two groups: (1) pairwise distances between GTEx-connected TE-TE/TE-IR/IR-IR pairs in the DGN network; and (2) those in random DGN networks generated by permuting genes/isoforms among themselves. Here, we generated random networks 10 times to estimate the null distribution.

### TWN replication using ARACNE

Using the same quantification of TE and IR levels in the GTEx data, we reconstructed ARACNE networks (Margolin et al. 2006) over TE and IR jointly from a Spearman correlation-based mutual information matrix using the minet R package (Meyer et al. 2008) for 16 tissues. Following similar procedures as for TWNs, we excluded edges between features of same gene, cross-mappable genes, and position-overlapped genes from downstream analysis. For each tissue, we computed the fraction of TWN edges that were also present in the ARACNE network for the matched tissue. We compared these results with the comparison of the ARACNE network with a random TWN generated by permuting gene/isoform labels.

### TWN hub ranking

We ordered the network hubs by degree centrality for each tissue according to the number of unique gene-level connections to avoid the effect of different numbers of isoforms per gene. To do this, we created a gene-level network from the original TWNs by keeping TE nodes as they were and grouping all isoforms of the same gene together to form a compound IR node. We put an edge between a compound IR node and a TE node (or another compound IR node) if any isoform of the compound had an edge with the TE node (or any isoform of the other compound) in the original TWN, and the weight was equal to the sum of absolute weights of all such edges in the original TWN. TE-TE and IR-TE hubs were ordered by the number of TE nodes they were connected with. TE-IR and IR-IR hubs were ordered by the number of compound IR nodes they were connected with. If multiple hubs had the same number of connections, ties were broken by the sum of corresponding edge weights.

### TWN hubs shared across tissues

We used rank-product (Zhong et al. 2014) to find hubs generally ranked highly in a set of tissues. We first ranked genes by the number of neighbors in the gene-level network. If a gene had no edge in the network, its rank was considered to be the number of genes with neighbors plus one. A gene's rank-product is the product of its ranks from each network. The top shared hub gene had the lowest rank-product.

### TWN hubs specific to a group of related tissues

To find hubs specific to a group of tissues, we used rank-product to rank hubs in both the target group of tissues and in all other tissues, separately. Then, we normalized ranks so that the top- and bottom-ranked hubs have a score of 1 and 0, respectively. Let the normalized rank of a gene in the target group of tissues and other tissues be *r*_*t*_ and *r*_*o*_, respectively. Then, the *F*-score for the gene (*r*),

(3) $$\begin{array}{c} r=\frac{2}{\frac{1}{r_{t}}+\frac{1}{1-r_{o}}}, \end{array}$$

will be high if it ranks highly in the target group but low in other tissues.

We computed related tissue-specific hubs for five groups of related tissues: (1) skin–sun exposed and skin–not sun exposed; (2) adipose–subcutaneous, adipose–visceral, and breast–mammary; (3) heart–left ventricle and skeletal muscle; (4) esophagus–mucosa and esophagus–muscularis; and (5) artery–aorta and artery–tibial.

### Tissue-Specific Networks

We built Tissue-Specific gene co-expression Networks using an unsupervised Bayesian biclustering model, BicMix on the gene level TPM measurements (from RSEM v1.2.20 [Li and Dewey 2011] as described above) from all of the GTEx v6 samples jointly (Gao et al. 2016). The expression data were normalized for GC content, length, and depth. For each tissue, we removed genes that had zero read counts in more than 90% of samples. We took the intersection of all remaining genes across the 50 tissues and only used those 15,589 genes for the analysis. All 50 tissue expression matrices were appended together and subsequently quantile-normalized within each gene across all tissues. We performed 40 runs of BicMix on these data and used the output from iteration 300 of the variational Expectation-Maximization algorithm. We set the hyperparameters for BicMix based on extensive simulation studies in prior work (Gao et al. 2013). We selected factors to build the tissue-specific covariance matrix estimate by including those for which nonzero factor values were exclusive to samples from the tissue of interest. We inverted these matrices and used GeneNet (Schäfer and Strimmer 2005b) with a confidence threshold of 0.8, as in previous work, to build TSNs for each run (Gao et al. 2016). For each tissue, we looked across the TSNs produced by each run (some runs did not produce a TSN) and included every edge that appeared in at least 25% of those networks in the final TSN. With this approach, we tried to build networks for all of the tissues but discarded TSNs for which there were fewer than five edges, resulting in 26 TSNs.

### *Cis*-eQTLs from TSNs

For each tissue in which we recovered a TSN, we used the same set of genes and expression values as described for TSN creation, prior to taking the intersection of genes across all tissues. PEER factors were used to quantify effects of unobserved confounding variables (Stegle et al. 2012). We optimized the number of PEER factors by tissue to a test chromosome (Chromosome 11) to maximize the number of identified *cis*-eQTLs. The linear model of Matrix-eQTL (Shabalin 2012) was used to test all SNPs within the 100 kb window of a gene's transcription start site or transcription end site (TES) using an additive linear model. We included in association mapping a tissue-specific number of PEER factors, sex, genotyping batch, and three genotype principal components. The correlation between SNP and gene expression levels was evaluated using the estimated t-statistic from this model. False discover y rate was calculated using BH. We used these *cis*-eQTLs for the *trans*-eQTL analysis for the TSN edge replication described below.

### *Trans*-eQTLs from TSNs

We computed *trans*-QTLs in two ways. First, we found the best *cis*-associated variant per gene (smallest *P*-value, from the *cis*-eQTLs described in the previous paragraph) in that tissue, if one existed, and measured association between that variant and every neighbor of that gene in the TSN using the linear model of Matrix-eQTL (Shabalin 2012). Second, we measured association between all variants within 20 kb of a gene's TSS and TES with each neighbor in the network using the linear model of Matrix-eQTL (Shabalin 2012). In both approaches, we controlled for the first three genotype principal components (PCs), sex, and platform, and used BH FDR ≤ 0.2 for multiple testing correction.

### *Trans*-splicing QTLs from TWNs

We computed *trans*-splicing QTLs using two approaches. In the first approach, we used the best *cis*-associated variant per gene (smallest *P*-value) located within 1 Mb from the transcription start site of the gene (The GTEx Consortium 2017). Then, for every TE node connected with an IR node in the network, we measured association between the gene's best *cis*-associated variant and all the isoform ratio neighbors using the linear model of Matrix-eQTL (Shabalin 2012), controlling for the first three genotype PCs and genotype platform. We corrected for false discovery (BH FDR ≤ 0.05). In the second approach, for each of the top 500 TE-IR hubs, we took all variants within 20 kb of the TSS and tested their association with isoforms located on a different chromosome and connected with the TE hub using Matrix-eQTL. Here, we used FDR ≤ 0.2 for the false discovery threshold.

### Software availability

Source code is available as Supplemental Code S1. It is also freely available on GitHub: https://github.com/battle-lab/twn_tsn.

## Data access

GTEx v6 data from this study have been submitted to dbGaP, under accession number phs000424.v6. TWNs for 16 tissues and TSNs for 26 tissues and 10 tissue groups are available at the GTEx portal (http://gtexportal.org). DGN cohort data are available by application through the National Institute of Mental Health (NIMH) Center for Collaborative Genomic Studies on Mental Disorders (www.nimhgenetics.org).

## GTEx Consortium

### Laboratory, Data Analysis & Coordinating Center (LDACC)—Analysis Working Group

François Aguet,^8^ Kristin G. Ardlie,^8^ Beryl B. Cummings,^8^,^9^ Ellen T. Gelfand,^8^ Gad Getz,^8^,^10^ Kane Hadley,^8^ Robert E. Handsaker,^8^,^11^ Katherine H. Huang,^8^ Seva Kashin,^8^,^11^ Konrad J. Karczewski,^8^,^9^ Monkol Lek,^8^,^9^ Xiao Li,^8^ Daniel G. MacArthur,^8^,^9^ Jared L. Nedzel,^8^ Duyen T. Nguyen,^8^ Michael S. Noble,^8^ Ayellet V. Segrè,^8^ Casandra A. Trowbridge,^8^ Taru Tukiainen,^8^,^9^

### Statistical Methods groups—Analysis Working Group

Nathan S. Abell,^12^,^13^ Brunilda Balliu,^13^ Ruth Barshir,^14^ Omer Basha,^14^ Alexis Battle,^15^ Gireesh K. Bogu,^16^,^17^ Andrew Brown,^18^,^19^,^20^ Christopher D. Brown,^21^ Stephane E. Castel,^22^,^23^ Lin S. Chen,^24^ Colby Chiang,^25^ Donald F. Conrad,^26^,^27^ Nancy J. Cox,^28^ Farhan N. Damani,^15^ Joe R. Davis,^12^,^13^ Olivier Delaneau,^18^,^19^,^20^ Emmanouil T. Dermitzakis,^18^,^19^,^20^ Barbara E. Engelhardt,^29^ Eleazar Eskin,^30^,^31^ Pedro G. Ferreira,^32^,^33^ Laure Frésard,^12^,^13^ Eric R. Gamazon,^28^,^34^,^35^ Diego Garrido-Martín,^16^,^17^ Ariel D.H. Gewirtz,^36^ Genna Gliner,^37^ Michael J. Gloudemans,^12^,^13^,^38^ Roderic Guigo,^16^,^17^,^39^ Ira M. Hall,^25^,^26^,^40^ Buhm Han,^41^ Yuan He,^42^ Farhad Hormozdiari,^30^ Cedric Howald,^18^,^19^,^20^ Hae Kyung Im,^43^ Brian Jo,^36^ Eun Yong Kang,^30^ Yungil Kim,^15^ Sarah Kim-Hellmuth,^22^,^23^ Tuuli Lappalainen,^22^,^23^ Gen Li,^44^ Xin Li,^13^ Boxiang Liu,^12^,^13^,^45^ Serghei Mangul,^30^ Mark I. McCarthy,^46^,^47^,^48^ Ian C. McDowell,^49^ Pejman Mohammadi,^22^,^23^ Jean Monlong,^16^,^17^,^50^ Stephen B. Montgomery,^12^,^13^ Manuel Muñoz-Aguirre,^16^,^17^,^51^ Anne W. Ndungu,^46^ Dan L. Nicolae,^43^,^52^,^53^ Andrew B. Nobel,^54^,^55^ Meritxell Oliva,^43^,^56^ Halit Ongen,^18^,^19^,^20^ John J. Palowitch,^54^ Nikolaos Panousis,^18^,^19^,^20^ Panagiotis Papasaikas,^16^,^17^ YoSon Park,^21^ Princy Parsana,^15^ Anthony J. Payne,^46^ Christine B. Peterson,^57^ Jie Quan,^58^ Ferran Reverter,^16^,^17^,^59^ Chiara Sabatti,^60^,^61^ Ashis Saha,^15^ Michael Sammeth,^62^ Alexandra J. Scott,^25^ Andrey A. Shabalin,^63^ Reza Sodaei,^16^,^17^ Matthew Stephens,^52^,^53^ Barbara E. Stranger,^43^,^56^,^64^ Benjamin J. Strober,^42^ Jae Hoon Sul,^65^ Emily K. Tsang,^13^,^38^ Sarah Urbut,^53^ Martijn van de Bunt,^46^,^47^ Gao Wang,^53^ Xiaoquan Wen,^66^ Fred A. Wright,^67^ Hualin S. Xi,^58^ Esti Yeger-Lotem,^14^,^68^ Zachary Zappala,^12^,^13^ Judith B. Zaugg,^69^ Yi-Hui Zhou,^67^

### Enhancing GTEx (eGTEx) groups

Joshua M. Akey,^36^,^70^ Daniel Bates,^71^ Joanne Chan,^12^ Lin S. Chen,^24^ Melina Claussnitzer,^8^,^72^,^73^ Kathryn Demanelis,^24^ Morgan Diegel,^71^ Jennifer A. Doherty,^74^ Andrew P. Feinberg,^42^,^75^,^76^,^77^ Marian S. Fernando,^43^,^56^ Jessica Halow,^71^ Kasper D. Hansen,^75^,^78^,^79^ Eric Haugen,^71^ Peter F. Hickey,^79^ Lei Hou,^8^,^80^ Farzana Jasmine,^24^ Ruiqi Jian,^12^ Lihua Jiang,^12^ Audra Johnson,^71^ Rajinder Kaul,^71^ Manolis Kellis,^8^,^80^ Muhammad G. Kibriya,^24^ Kristen Lee,^71^ Jin Billy Li,^12^ Qin Li,^12^ Xiao Li,^12^ Jessica Lin,^12^,^81^ Shin Lin,^12^,^82^ Sandra Linder,^12^,^13^ Caroline Linke,^43^,^56^ Yaping Liu,^8^,^80^ Matthew T. Maurano,^83^ Benoit Molinie,^8^ Stephen B. Montgomery,^12^,^13^ Jemma Nelson,^71^ Fidencio J. Neri,^71^ Meritxell Oliva,^43^,^56^ Yongjin Park,^8^,^80^ Brandon L. Pierce,^24^ Nicola J. Rinaldi,^8^,^80^ Lindsay F. Rizzardi,^75^ Richard Sandstrom,^71^ Andrew Skol,^43^,^56^,^64^ Kevin S. Smith,^12^,^13^ Michael P. Snyder,^12^ John Stamatoyannopoulos,^71^,^81^,^84^ Barbara E. Stranger,^43^,^56^,^64^ Hua Tang,^12^ Emily K. Tsang,^13^,^38^ Li Wang,^8^ Meng Wang,^12^ Nicholas Van Wittenberghe,^8^ Fan Wu,^43^,^56^ Rui Zhang,^12^

### NIH Common Fund

Concepcion R. Nierras,^85^

### NIH/NCI

Philip A. Branton,^86^ Latarsha J. Carithers,^86^,^87^ Ping Guan,^86^ Helen M. Moore,^86^ Abhi Rao,^86^ Jimmie B. Vaught,^86^

### NIH/NHGRI

Sarah E. Gould,^88^ Nicole C. Lockart,^88^ Casey Martin,^88^ Jeffery P. Struewing,^88^ Simona Volpi,^88^

### NIH/NIMH

Anjene M. Addington,^89^ Susan E. Koester,^89^

### NIH/NIDA

A. Roger Little,^90^

### Biospecimen Collection Source Site—NDRI

Lori E. Brigham,^91^ Richard Hasz,^92^ Marcus Hunter,^93^ Christopher Johns,^94^ Mark Johnson,^95^ Gene Kopen,^96^ William F. Leinweber,^96^ John T. Lonsdale,^96^ Alisa McDonald,^96^ Bernadette Mestichelli,^96^ Kevin Myer,^93^ Brian Roe,^93^ Michael Salvatore,^96^ Saboor Shad,^96^ Jeffrey A. Thomas,^96^ Gary Walters,^95^ Michael Washington,^95^ Joseph Wheeler,^94^

### Biospecimen Collection Source Site—RPCI

Jason Bridge,^97^ Barbara A. Foster,^98^ Bryan M. Gillard,^98^ Ellen Karasik,^98^ Rachna Kumar,^98^ Mark Miklos,^97^ Michael T. Moser,^98^

### Biospecimen Core Resource—VARI

Scott D. Jewell,^99^ Robert G. Montroy,^99^ Daniel C. Rohrer,^99^ Dana R. Valley,^99^

### Brain Bank Repository—University of Miami Brain Endowment Bank

David A. Davis,^100^ Deborah C. Mash,^100^

### Leidos Biomedical—Project Management

Anita H. Undale,^101^ Anna M. Smith,^102^ David E. Tabor,^102^ Nancy V. Roche,^102^ Jeffrey A. McLean,^102^ Negin Vatanian,^102^ Karna L. Robinson,^102^ Leslie Sobin,^102^ Mary E. Barcus,^103^ Kimberly M. Valentino,^102^ Liqun Qi,^102^ Steven Hunter,^102^ Pushpa Hariharan,^102^ Shilpi Singh,^102^ Ki Sung Um,^102^ Takunda Matose,^102^ Maria M. Tomaszewski,^102^

### ELSI Study

Laura K. Barker,^104^ Maghboeba Mosavel,^105^ Laura A. Siminoff,^104^ Heather M. Traino,^104^

### Genome Browser Data Integration & Visualization—EBI

Paul Flicek,^106^ Thomas Juettemann,^106^ Magali Ruffier,^106^ Dan Sheppard,^106^ Kieron Taylor,^106^ Stephen J. Trevanion,^106^ Daniel R. Zerbino,^106^

### Genome Browser Data Integration & Visualization—UCSC Genomics Institute, University of California Santa Cruz

Brian Craft,^107^ Mary Goldman,^107^ Maximilian Haeussler,^107^ W. James Kent,^107^ Christopher M. Lee,^107^ Benedict Paten,^107^ Kate R. Rosenbloom,^107^ John Vivian,^107^ Jingchun Zhu,^107^

## Supplementary Material

Supplemental Material

## Footnotes

8 The Broad Institute of Massachusetts Institute of Technology and Harvard University, Cambridge, MA 02142, USA

9 Analytic and Translational Genetics Unit, Massachusetts General Hospital, Boston, MA 02114, USA

10 Massachusetts General Hospital Cancer Center and Dept. of Pathology, Massachusetts General Hospital, Boston, MA 02114, USA

11 Department of Genetics, Harvard Medical School, Boston, MA 02114, USA

12 Department of Genetics, Stanford University, Stanford, CA 94305, USA

13 Department of Pathology, Stanford University, Stanford, CA 94305, USA

14 Department of Clinical Biochemistry and Pharmacology, Faculty of Health Sciences, Ben-Gurion University of the Negev, Beer-Sheva 84105, Israel

15 Department of Computer Science, Johns Hopkins University, Baltimore, MD 21218, USA

16 Centre for Genomic Regulation (CRG), The Barcelona Institute for Science and Technology, 08003 Barcelona, Spain

17 Universitat Pompeu Fabra (UPF), 08003 Barcelona, Spain

18 Department of Genetic Medicine and Development, University of Geneva Medical School, 1211 Geneva, Switzerland

19 Institute for Genetics and Genomics in Geneva (iG3), University of Geneva, 1211 Geneva, Switzerland

20 Swiss Institute of Bioinformatics, 1211 Geneva, Switzerland

21 Department of Genetics, Perelman School of Medicine, University of Pennsylvania, Philadelphia, PA 19104, USA

22 New York Genome Center, New York, NY 10013, USA

23 Department of Systems Biology, Columbia University Medical Center, New York, NY 10032, USA

24 Department of Public Health Sciences, The University of Chicago, Chicago, IL 60637, USA

25 McDonnell Genome Institute, Washington University School of Medicine, St. Louis, MO 63108, USA

26 Department of Genetics, Washington University School of Medicine, St. Louis, MO 63108, USA

27 Department of Pathology & Immunology, Washington University School of Medicine, St. Louis, MO 63108, USA

28 Division of Genetic Medicine, Department of Medicine, Vanderbilt University Medical Center, Nashville, TN 37232, USA

29 Department of Computer Science, Center for Statistics and Machine Learning, Princeton University, Princeton, NJ 08540, USA

30 Department of Computer Science, University of California, Los Angeles, CA 90095, USA

31 Department of Human Genetics, University of California, Los Angeles, CA 90095, USA

32 Instituto de Investigação e Inovação em Saúde (i3S), Universidade do Porto, 4200-135 Porto, Portugal

33 Institute of Molecular Pathology and Immunology (IPATIMUP), University of Porto, 4200-625 Porto, Portugal

34 Department of Clinical Epidemiology, Biostatistics and Bioinformatics, Academic Medical Center, University of Amsterdam, 1105 AZ Amsterdam, The Netherlands

35 Department of Psychiatry, Academic Medical Center, University of Amsterdam, 1105 AZ Amsterdam, The Netherlands

36 Lewis Sigler Institute, Princeton University, Princeton, NJ 08540, USA

37 Department of Operations Research and Financial Engineering, Princeton University, Princeton, NJ 08540, USA

38 Biomedical Informatics Program, Stanford University, Stanford, CA 94305, USA

39 Institut Hospital del Mar d'Investigacions Mèdiques (IMIM), 08003 Barcelona, Spain

40 Department of Medicine, Washington University School of Medicine, St. Louis, MO 63108, USA.

41 Department of Convergence Medicine, University of Ulsan College of Medicine, Asan Medical Center, Seoul 138-736, South Korea

42 Department of Biomedical Engineering, Johns Hopkins University, Baltimore, MD 21218, USA

43 Section of Genetic Medicine, Department of Medicine, The University of Chicago, Chicago, IL 60637, USA

44 Department of Biostatistics, Mailman School of Public Health, Columbia University, New York, NY 10032, USA

45 Department of Biology, Stanford University, Stanford, CA 94305, USA

46 Wellcome Trust Centre for Human Genetics, Nuffield Department of Medicine, University of Oxford, Oxford, OX3 7BN, UK

47 Oxford Centre for Diabetes, Endocrinology and Metabolism, University of Oxford, Churchill Hospital, Oxford, OX3 7LE, UK

48 Oxford NIHR Biomedical Research Centre, Churchill Hospital, Oxford, OX3 7LJ, UK

49 Computational Biology & Bioinformatics Graduate Program, Duke University, Durham, NC 27708, USA

50 Human Genetics Department, McGill University, Montreal, Quebec H3A 0G1, Canada

51 Departament d'Estadística i Investigació Operativa, Universitat Politècnica de Catalunya, 08034 Barcelona, Spain

52 Department of Statistics, The University of Chicago, Chicago, IL 60637, USA

53 Department of Human Genetics, The University of Chicago, Chicago, IL 60637, USA

54 Department of Statistics and Operations Research, University of North Carolina, Chapel Hill, NC 27599, USA

55 Department of Biostatistics, University of North Carolina, Chapel Hill, NC 27599, USA

56 Institute for Genomics and Systems Biology, The University of Chicago, Chicago, IL 60637, USA

57 Department of Biostatistics, The University of Texas MD Anderson Cancer Center, Houston, TX 77030, USA

58 Computational Sciences, Pfizer Inc, Cambridge, MA 02139, USA

59 Universitat de Barcelona, 08028 Barcelona, Catalonia, Spain

60 Department of Biomedical Data Science, Stanford University, Stanford, CA 94305, USA

61 Department of Statistics, Stanford University, Stanford, CA 94305, USA

62 Institute of Biophysics Carlos Chagas Filho (IBCCF), Federal University of Rio de Janeiro (UFRJ), 21941902 Rio de Janeiro, Brazil

63 Department of Psychiatry, University of Utah, Salt Lake City, UT 84108, USA

64 Center for Data Intensive Science, The University of Chicago, Chicago, IL 60637, USA

65 Department of Psychiatry and Biobehavioral Sciences, University of California, Los Angeles, CA 90095, USA

66 Department of Biostatistics, University of Michigan, Ann Arbor, MI 48109, USA

67 Bioinformatics Research Center and Departments of Statistics and Biological Sciences, North Carolina State University, Raleigh, NC 27695, USA

68 National Institute for Biotechnology in the Negev, Beer-Sheva, 84105 Israel

69 European Molecular Biology Laboratory, 69117 Heidelberg, Germany

70 Department of Ecology and Evolutionary Biology, Princeton University, Princeton, NJ 08540, USA

71 Altius Institute for Biomedical Sciences, Seattle, WA 98121, USA

72 Beth Israel Deaconess Medical Center, Harvard Medical School, Boston, MA 02215, USA

73 University of Hohenheim, 70599 Stuttgart, Germany

74 Huntsman Cancer Institute, Department of Population Health Sciences, University of Utah, Salt Lake City, UT 84112, USA

75 Center for Epigenetics, Johns Hopkins University School of Medicine, Baltimore, MD 21205, USA

76 Department of Medicine, Johns Hopkins University School of Medicine, Baltimore, MD 21205, USA

77 Department of Mental Health, Johns Hopkins University School of Public Health, Baltimore, MD 21205, USA

78 McKusick-Nathans Institute of Genetic Medicine, Johns Hopkins School of Medicine, Baltimore, MD 21205, USA

79 Department of Biostatistics, Johns Hopkins University, Baltimore, MD 21205, USA

80 Computer Science and Artificial Intelligence Laboratory, Massachusetts Institute of Technology, Cambridge, MA 02139, USA

81 Department of Medicine, University of Washington, Seattle, WA 98195, USA

82 Division of Cardiology, University of Washington, Seattle, WA 98195, USA

83 Institute for Systems Genetics, New York University Langone Medical Center, New York, NY 10016, USA

84 Department of Genome Sciences, University of Washington, Seattle, WA 98195, USA

85 Office of Strategic Coordination, Division of Program Coordination, Planning and Strategic Initiatives, Office of the Director, NIH, Rockville, MD 20852, USA

86 Biorepositories and Biospecimen Research Branch, Division of Cancer Treatment and Diagnosis, National Cancer Institute, Bethesda, MD 20892, USA

87 National Institute of Dental and Craniofacial Research, Bethesda, MD 20892, USA

88 Division of Genomic Medicine, National Human Genome Research Institute, Rockville, MD 20852, USA

89 Division of Neuroscience and Basic Behavioral Science, National Institute of Mental Health, NIH, Bethesda, MD 20892, USA

90 Division of Neuroscience and Behavior, National Institute on Drug Abuse, NIH, Bethesda, MD 20892, USA

91 Washington Regional Transplant Community, Falls Church, VA 22003, USA

92 Gift of Life Donor Program, Philadelphia, PA 19103, USA

93 LifeGift, Houston, TX 77055, USA

94 Center for Organ Recovery and Education, Pittsburgh, PA 15238, USA

95 LifeNet Health, Virginia Beach, VA 23453, USA

96 National Disease Research Interchange, Philadelphia, PA 19103, USA

97 Unyts, Buffalo, NY 14203, USA

98 Pharmacology and Therapeutics, Roswell Park Cancer Institute, Buffalo, NY 14263, USA

99 Van Andel Research Institute, Grand Rapids, MI 49503, USA

100 Brain Endowment Bank, Miller School of Medicine, University of Miami, Miami, FL 33136, USA

101 National Institute of Allergy and Infectious Diseases, NIH, Rockville, MD 20852, USA

102 Biospecimen Research Group, Clinical Research Directorate, Leidos Biomedical Research, Inc., Rockville, MD 20852, USA

103 Leidos Biomedical Research, Inc., Frederick, MD 21701, USA

104 Temple University, Philadelphia, PA 19122, USA

105 Department of Health Behavior and Policy, School of Medicine, Virginia Commonwealth University, Richmond, VA 23298, USA

106 European Molecular Biology Laboratory, European Bioinformatics Institute, Hinxton CB10 1SD, UK

107 UCSC Genomics Institute, University of California Santa Cruz, Santa Cruz, CA 95064, USA
