# Supplementary material for: Co-expression networks reveal the tissue-specific regulation of transcription and splicing
Source: Genome Res. 2017 Nov;27(11):1843–58. doi: 10.1101/gr.216721.116 (PMC5668942; doi:10.1101/gr.216721.116)
Supplement: Supplemental Material [file supp_27_11_1843__index.html]

Co-expression networks reveal the tissue-specific regulation of transcription and splicing — Co-expression networks reveal the tissue-specific regulation of transcription and splicing — Supplemental Material 

# Co-expression networks reveal the tissue-specific regulation of transcription and splicing

## Supplemental Material

- Supplemental\_Code\_S1.zip
- Supplemental\_Data\_S5.xlsx
- Supplemental\_Data\_S4.xlsx
- Supplemental\_Data\_S3.xlsx
- Supplemental\_Data\_S2.xlsx
- Supplemental\_Data\_S1.xlsx
- Supplemental\_Materials.pdf
