# Supplementary material for: Co-expression networks reveal the tissue-specific regulation of transcription and splicing
Source: Genome Res. 2017 Nov;27(11):1843–58. doi: 10.1101/gr.216721.116 (PMC5668942; doi:10.1101/gr.216721.116)
Supplement: Supplemental Material [file supp_gr.216721.116_Supplemental_Code_S1.zip › twn_tsn/README.pdf]

This repository Search Pull requests Issues Marketplace Gist

battle-lab / twn_tsn

Unwatch 4 Star 0 Fork 0

Code Issues 0 Pull requests 0 Projects 0 Wiki Settings Insights

Branch: master twn_tsn / README.md Find file Copy path

alorchhota fix readme formatting ce2c50c 2 minutes ago

2 contributors

153 lines (107 sloc) 10.6 KB Raw Blame History

This repository contains code to reconstruct **transcriptome-wide networks (TWNs)** and **tissue-specific networks (TSNs)**, as described in the paper titled Co-expression networks reveal the tissue-specific regulation of transcription and splicing. Instructions for each type of network are given below.

Transcriptome-Wide Network (TWN)

A transcriptome-wide network (TWN) is an undirected network consturcted over total expression and isoform ratio jointly. Codes to reconstruct a TWN reside in the *twn* folder, here referred as *twn directory*.

Installation

All the scripts here were written and run in Linux environment with matlab 2013a, and R 3.1.1.

1. QUIC() function must be running in matlab. Please download the MEX package archive v1.2 from http://www.cs.utexas.edu/~sustik/QUIC/, unzip it, and compile it following the instructions in README file inside QUIC package. By default, QUIC package is expected to be inside the *twn directory* as a folder named *QUIC*, but you may install it in any other directory and configure it in the *settings.sh* file in the *twn directory*.
2. 2 packages must be installed in R: argparser, and data.table.

Files and Formats

- Total Expression File: Tab delimited file containing corrected total expression data. Each row represent a sample and each column represents a gene. First row and first column contain gene ids and sample ids, respectively.
- Isoform Ratio File: Tab delimited file containing corrected isoform ratio data. Each row represent a sample and each column represents an isoform. First row and first column contain isoform ids and sample ids, respectively. Samples in both Total Expression and Isoform Ratio files have to be in the same order.
- Gene Annotation File: Tab delimited file with two columns: *gene_id*, and *ensembl_gene_id*.
- Isoform Annotation File: Tab delimited file with three columns: *transcript_id* (isoform id), *gene_id*, and *ensembl_gene_id*.
- Positional Overlap File: Tab delimited file with two columns (*gene1*, *gene2*) containing pair of genes (ensembl gene ids) with positional overlap in the genome.
- Cross Mappability File: Tab delimited file with two columns (*gene1*, *gene2*) containing pair of genes (ensembl gene ids) with cross mappability (see the paper for details).

Example Data

You may download example data from here. Please unzip the file and put inside the *twn directory* (in the directory where twn.sh file is). If you do not keep data in this directory, please update the settings file accordingly.

Settings

settings.sh file (inside *twn directory*) contains necessary information to reconstruct transcriptome-wide network (i.e., to run *twn.sh*). You may need to edit this file to customize your settings.

Are the installations and the settings are OK?

To check if all pre-requisites have been successfully installed, and the setting file contains valid configuration, run the following command.

```
sh ./check_prerequisites.sh
```

How to reconstruct a TWN?

You have to run the script twn.sh with the following arguments.

- Total expression data file path
- Isoform ratio data file path
- Output file prefix
- 5 penalty parameters (lambda_tt, lambda_ti, lambda_ii, lambda_d, lambda_s). see the paper for details.

Sample shell script code

```
# parameters
twn_dir='/home/asaha6/github/twn_tsn/twn'    # twn directory *** change it ***
te_fn="$twn_dir/data/demo/TE_demo.txt"       # total expression file
ir_fn="$twn_dir/data/demo/IR_demo.txt"       # isoform ratio file
out_fn_prefix="$twn_dir/demo/output_demo"    # output file prefix
l_tt=0.5      # penalty parameter
l_ti=0.4      # penalty parameter
l_ii=0.4      # penalty parameter
l_d=0         # penalty parameter
l_s=0.05      # penalty parameter

# move to the twn source directory
cd $twn_dir

# run twn
sh ./twn.sh $te_fn $ir_fn $out_fn_prefix $l_tt $l_ti $l_ii $l_d $l_s
```

For convenience, a sample script (*sample_script_to_run_twn.sh*) has been provided in the demo folder (*twn/demo/*) to construct a TWN. Remember to change the value of *twn_dir* in the sample script to reflect your own *twn directory*.

After a successful run, you will find the following 4 files with starting with the given output file prefix.

- *[output prefix].twn.txt*: It is the most important output file. It is a tab delimited file with four columns representing the constructed transcriptome-wide network. Here, the first two columns, containing either a total expression id or an isoform id, together represent an edge. The third column represents the type of the edge: 1 for an edge strictly between two total expressions, 2 for an edge between a total expression and an isoform, 3 for an edge strictly between two isoforms. The fourth column represents the edge weight.
- *[output prefix].quic.txt*: The format of this file is similar to *[output prefix].twn.txt*, but it also contains the filtered edges from the inverse covariance matrix estimated by QUIC.
- *[output prefix].quic.info*: It contains a few outptus from QUIC - i) the optimum objective value obtained from QUIC, ii) the number of iteration needed for the optimization, and iii) the time needed to finish the optimization.
- *[output prefix]_data_status.txt*: An intermediate file used to contain whether given data are correctly formatted or not.

Tissue-Specific Network (TSN)

A Tissue Specific Network (TSN) is an undirected network constructed over total gene expressions using Bicmix. It contains an edges between two nodes if the nodes are co-expressed only in the tissue of interest. Codes to reconstruct a TSN reside in the *tsn* folder, here referred as *tsn directory*.

Installation

All the scripts were written and run in Linux environment with R 3.3.1.

1. This assumes that you have already run Bicmix. Bicmix code is available here: https://www.cs.princeton.edu/~bee/software.html A sample batch perl script for creating scripts for multiple bicmix jobs is below:

```
##### START SAMPLE PERL SCRIPT

$heredoc2 = <<END;

bicluster_mixture_simul_up  --y \$data_i --nf \$fac --sep tab --out \${dir}/\$result --interval \${interval
END

my $fac=1000;
my $interval=150;
my $dir="name of output directory"; #TODO: EDIT
`mkdir $dir`;
my $data_i =("name of expression data file"); #TODO: EDIT

  for(my $iseed=1;$iseed<40;$iseed++){ #TODO: Change iseed limit based on how many bicmix runs are desired
        my $result="${data_i}_fac${fac}_interval${interval}_seed${iseed}"; #Recommended dir names to be com
        `mkdir $dir/$result/`;
        my $file="${result}.sh";
        print "$file\n";
        open FILE,">$dir/$file" or die;
        print FILE "#!/bin/bash\n\n";
        print FILE "data_i=$data_i\n";
        print FILE "fac=$fac\n";
        print FILE "interval=$interval\n";
        print FILE "dir=$dir\n";
        print FILE "result=$result\n";
        print FILE $heredoc2;
        close FILE;
        #`./$dir/$file`; TODO: These shell scripts all need to be run
  }

####### END SAMPLE PERL SCRIPT -remember to run all the shell scripts you created in $dir
```

2. argparser package must be installed in R.

Files and Formats

- Output Directory (-out): Path to directory in which results will be written
- File with Gene Names (Row labels) for the expression matrix (-gn): Path to file with one ensembl gene label per line, in the same order as in the expression matrix
- File with Sample Size for each tissue used (-ss): Path to file with each line containing the number of samples from that tissue. The order of tissues is the order in which they appear in the expression matrix
- File with Covariate Matrix (-cov): Path to tab delimited file with n rows and x columns, where n is the number of samples in the expression matrix and x is the number of covariates. The matrix is filled in with the corresponding value of the covariate for each sample. There are no column or row names in this file. NOTE: recover_TSN.R will attempt to recover networks for each of these covariates. If you have more covariates than you are interested in getting specific networks for, you should delete those columns of the matrix.
- File with covariate names (-cn): Path to file with x rows, where each row lists the label of the corresponding column in the covariate matrix file. In other words, this should have the column names of the -cov file, with one label per row. NOTE: If you have edited the covariate matrix, remember to also edit the covariate names file.
- Directory with Bicmix outputs (-rd): Path to directory of bicmix results. This directory should contain one directory for each run of bicmix. The path to this directory should also include the name of the subdirectories (results for each run of bicmix) up to the unique number at the end. For example, in the sample perl file above we named these subdirectories ${data_i}_fac${fac}_interval${interval}_seed${iseed}, so this path would be "/path to larger results directory/${data_i}_fac${fac}_interval${interval}_seed". (obviously those are perl variables so the values would be there instead)
- Iteration (-it): Iteration number (ie 300) at which to use the results from Bicmix, since each run will run at its own pace and most likely finish at a different iteration.
- Number of runs of bicmix (-nr): This assumes that the directories partially named by the -rd path are numbered in order from 1 to whatever number you input here.
- Duplication Threshold (-thresh): Default is 4 -- meaning that edges in the TSNs are required to have shown up in at least (100/4)=25% of the bicmix runs which identified significant tissue-specific edges.
- GeneNet probability that an edge is nonzero (-gn): Default is 0.8

How to reconstruct a TSN?

You have to run the script recover_TSN.R with the arguments listed above

After running that script, you will find several files in the given output directory. For each covariate where a specific network was recovered, there will be two files: both will start with the name of the covariate and one will end with _nodes.csv, the other with _edges.csv. Those two files will contain the information for the covariate specific networks.

How to cite (it will be updated once the paper is published)

Saha A, Kim Y, Gewirtz ADH, Jo B, Gao C, McDowell IC, GTEx Consortium, Engelhardt BE, Battle A. 2016. Co-expression networks reveal the tissue-specific regulation of transcription and splicing. bioRxiv. http://biorxiv.org/content/early/2016/10/02/078741.abstract.

Contact

Ashis Saha (ashis@jhu.edu)
Ariel Gewirtz (gewirtz@princeton.edu)

© 2017 GitHub, Inc. Terms Privacy Security Status Help Contact GitHub API Training Shop Blog About
