# Supplementary material for: Co-expression networks reveal the tissue-specific regulation of transcription and splicing
Source: Genome Res. 2017 Nov;27(11):1843–58. doi: 10.1101/gr.216721.116 (PMC5668942; doi:10.1101/gr.216721.116)
Supplement: Supplemental Material [file supp_gr.216721.116_Supplemental_Materials.pdf]

Supplementary materials for: Co-expression networks reveal the tissue-specific regulation of transcription and splicing

Ashis Saha[1], Yungil Kim†[1], Ariel D. Gewirtz†[2], Brian Jo[2], Chuan Gao[3], Ian C. McDowell[3], The GTEx Consortium, Barbara E. Engelhardt[4,*], and Alexis Battle[5,*]

[1]Department of Computer Science, Johns Hopkins University, Baltimore, MD, USA
[2]Department of Quantitative and Computational Biology, Princeton University, Princeton, NJ, USA
[3]Program in Computational Biology and Bioinformatics, Duke University, Durham, NC, USA
[4]Department of Computer Science and Center for Statistics and Machine Learning, Princeton University, Princeton, NJ, USA[†]Denotes equal authorship.
[*]Corresponding authors: ajbattle@cs.jhu.edu, bee@princeton.edu

Supplementary Methods

RNA-seq data from GTEx project data

The NIH Common Fund's Genotype-Tissue Expression (GTEx) consortium (The GTEx Consortium, 2015)provides RNA-seq and microarray experiments. The original GTEx RNA-seq samples have been obtained from recently deceased donors (samples collected within 24 hours), between ages 21 and 70, BMI 18.5 to 35, and not under exclusionary medical criteria such as whole-blood transfusion within 24 hours or infection with HIV. The blood samples have been extracted for both genotyping with Illumina HumanOmni 2.5M and 5M BeadChips, as well as EBV-transformation of lymphoblastoids into cell lines. Then, biopsies from a set of tissues from different body sites (averaging about 28 per individual) have been obtained, stabilized with PAXgene Tissue kits, and then shipped to designated facilities for paraffin embedding, sectioning, and analyses of histology. After quality control protocols that checked for evidence of autolysis, inflammation and other pathology that could affect RNA-seq results, the stabilized tissue samples were sent to sequencing facilities for DNA/RNA extraction from the samples and performing

both microarray and RNA-seq experiments. In particular, the RNA-seq experiments were performed with Illumina HiSeq 2000 following the TrueSeq RNA protocol, yielding 76-bp paired-end reads averaging approximately 50 million reads per sample. As a result, we have 8,551 total experiments from 449 individuals for phase 1.

RNA sequencing alignment and transcript quantification

The RNA-seq processing pipeline follows previously described steps (McDowell et al., 2016). Adapter sequences and overrepresented contaminant sequences, identified by FastQC (v.0.10.1) (Andrews, 2010), were trimmed using Trimmomatic (v.0.30) (Bolger et al., 2014) with 2 seed mismatches and a simple clip threshold of 20. Leading and trailing nucleotides (low quality or Ns) were trimmed from all reads until a canonical base was encountered with quality greater than 3. For adaptive quality trimming, reads were scanned with a 4-base sliding window, trimming when the average quality per base dropped below 20. Any remaining sequences shorter than 30 nucleotides were discarded.

We aligned the RNA-seq reads using the STAR aligner in 2-pass mode (Dobin et al., 2013). After preparing the genome with STAR aligner `genomeGenerate` mode using a splice junction database (sjdbGTFfile) set to GENCODE v.19 annotation, the splice junction database overhang (sjdbOverhang) set to 75 bp, and defaults for all remaining settings. STAR aligner alignReads mode was run using default settings except `outFilterMultimapNmax` was set to 1 so that only uniquely mapping reads were retained.

We performed transcript and gene quantification using RSEM v1.2.20 (Li and Dewey, 2011). We used default settings using paired-end aware quantification.

Genotypes from GTEx data

The 2.5M and 5M BeadChip genotypes were merged to yield approximately 1.9 million genotyped SNPs. A greater set of genotypes were imputed using IMPUTE2 (Howie et al., 2009), yielding a satisfactory distribution of imputation scores for MAF ≥ 0.01 (mean INFO of 0.888 and median of 0.951 for variants with MAF between 0.01 and 0.05). The genotypes were filtered for MAF ≥ 0.05, leaving approximately 6 million variants. In order to take full advantage of SNP imputation, we used continuous (MLE of the dosage, ranging from 0 to 2) genotypes in association mapping. The genotype-level principle components were computed with the imputed genotypes.

Selection of smaller subset of genes and isoforms

For computational tractability, we selected 6,000 genes and 9,000 isoforms in each tissue from available genes and isoforms that passed other filtering steps. To do so, we first considered genes or isoforms if > 10 samples have TPM > 2 or reads > 6. To obtain the final set of genes, we first considered the top 9,000 genes based on their average expression levels and then selected the top $6,000$ highly variable genes across individuals. Similarly, to obtain the final set of isoforms, we first considered the $13,500$ genes with the highest expressed isoform levels on average. We reduced this to 11,25 genes based on the entropy of isoform ratios across individuals, normalized by the maximum entropy possible with the same number of isoforms, and finally took the top 9,000 most highly variable isoforms in terms of TPM values.

Gene mappability computation

We first downloaded mappability scores of all 75-mers and 36-mers in the human reference genome (hg19) from the UCSC Genome Browser for exonic regions and untranslated regions (UTRs), respectively (accession: wgEncodeEH000318, wgEncodeEH00032) (Derrien et al., 2012). For each gene, we then measured mappability scores for either exonic regions or UTRs with corresponding k-mers matched to the regions and aggregated the mappability scores for two regions by computing their weighted average. The weights were proportional to the total length of exonic regions and UTRs.

Splicing and RNA binding enrichment in top TE-IR hubs

We downloaded a list of human genes annotated with *RNA Splicing* (GO:0008380) and *RNA Binding* (GO:0003723) using topGO (Alexa and Rahnenfuhrer, 2016). We computed the enrichment of these RNA splicing and RNA binding genes in top 500 TE-IR hubs using Fisher's exact test. The set of all genes represented in the corresponding network was used as background.

TF-Target Enrichment in TE-TE edges

We downloaded transcription factors (TFs) and their known targets from ChEA (Lachmann et al., 2010). We measured the number of known TF-target relationships captured by a network, i.e., a TF and its target's total expression nodes were directly connected with each other. We generated the null distribution of the number of known TF-target relationships by computing same test statistics for random networks,

generated by permuting gene names among network nodes 1000 times. Then, we computed the empirical p-value as the proportion of those iterations for which the random network had at least as many known TF-Target edges as the test network. We fitted a Weibull distribution on the log(1+fraction of known TF-Target edges) to quantify the p-values.

Per pathway enrichment in TE-TE edges

We downloaded Reactome and KEGG pathway genes from the Molecular Signature Database (c2.cp.reactome.v5.1 and c2.cp.kegg.v5.1) (Subramanian et al., 2005). For each Reactome (or KEGG) pathway with at least ten genes, we tested whether the genes in the pathway had significantly smaller pairwise distances in our network than those in a random network, generated by permuting gene names among nodes in a network, using a Wilcoxon rank-sum test (Bonferroni corrected $p \leq 0.05$).

Shared pathway enrichment in TWN edges

We used Fisher's exact test to check whether or not our TWNs were enriched with edges between genes that participate in the same pathway. The null hypothesis was that two genes sharing an edge did not come from the same pathway.

Proportion of unique TWN hubs

For each type of hub in each tissue, we computed the proportion of top 100 hubs of the tissue of interest that did not appear in the list of top 500 hubs of any other tissue. We evaluated if the proportions in TE hubs (TE-TE and TE-IR hubs) were significantly larger than those in matched IR hubs (IR-TE and IR-IR hubs, respectively) using one sided Wilcoxon signed rank test in R. We also evaluated if the proportions in TE-TE hubs were significantly different than those in TE-IR hubs using two sided Wilcoxon signed rank test in R.

Differential expression of unique TWN hubs

A hub was considered differentially expressed in a tissue compared to all other tissues if the mean fold change was at least 1.5 for the hub gene (TE-TE or TE-IR hub) or any of its isoforms (IR-TE or IR-IR

hub), i.e., the mean TPM within a tissue was greater than 1.5 times or less than 1/1.5 times the mean TPM in the rest of the tissues.

Tissue groups for TSNs

We considered 10 groups: 1) all 13 brain tissues; 2) two adipose tissues and *breast – mammary*; 3) two heart tissues and three artery tissues; 4) four digestive tissues; 5) two adipose tissues; 6) two skin tissues; 7) three artery tissues; 8) five gland tissues; 9) two colon tissues; and 10) three esophagus tissues

Supplemental Figures

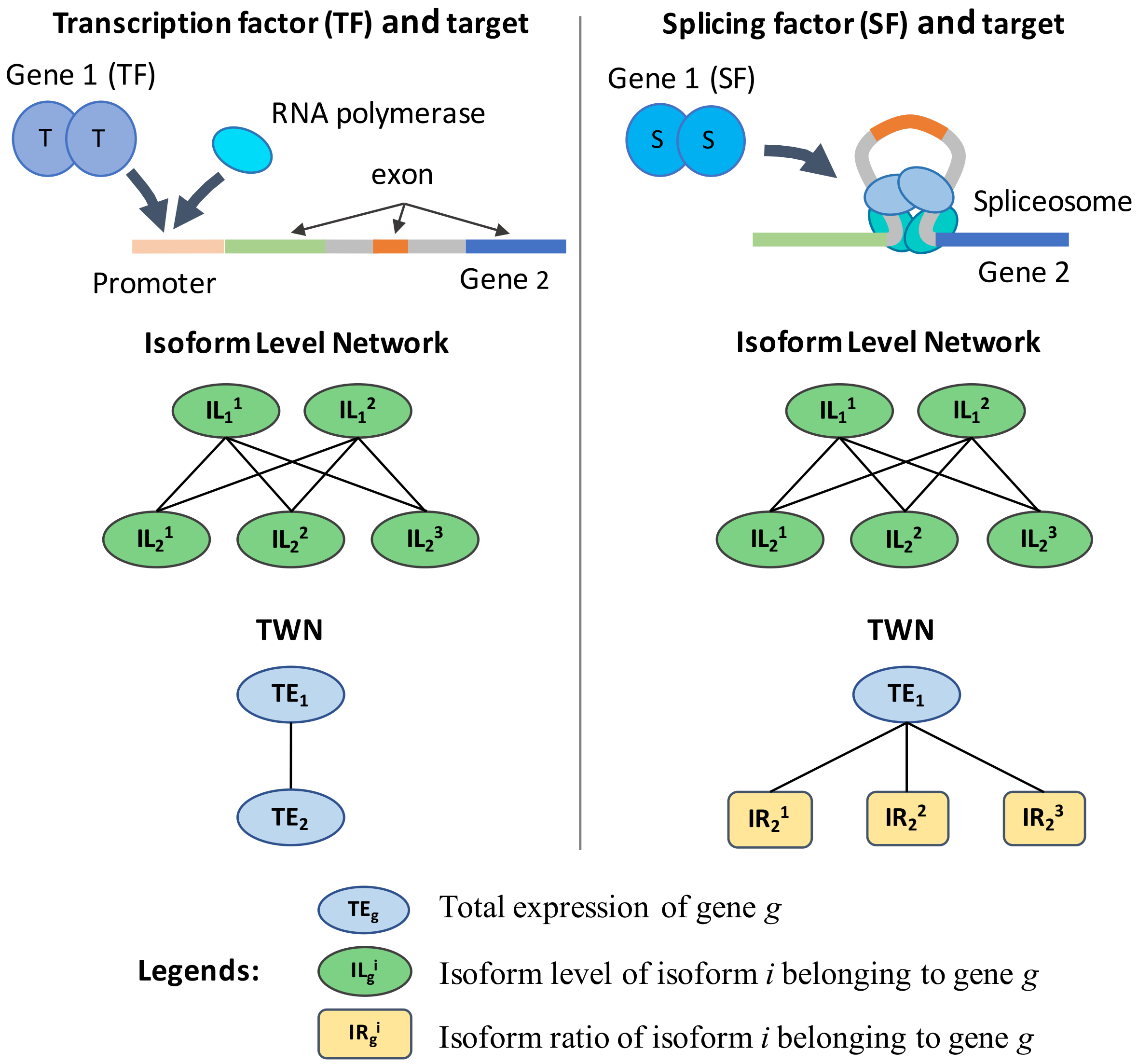

Figure 1. Difference between a standard isoform level network and a transcriptome-wide network (TWN). Left: Standard network and TWN representation when a transcription factor (Gene 1 with 2 isoforms) regulates transcription of its target (Gene 2 with 3 isoforms). Right: Standard and TWN representation when a splicing factor (Gene 1 with 2 isoforms) regulates splicing of its target (Gene 2 with 3 isoforms). The standard representation does not distinguish between transcriptional and splicing regulation, whereas the TWN does.

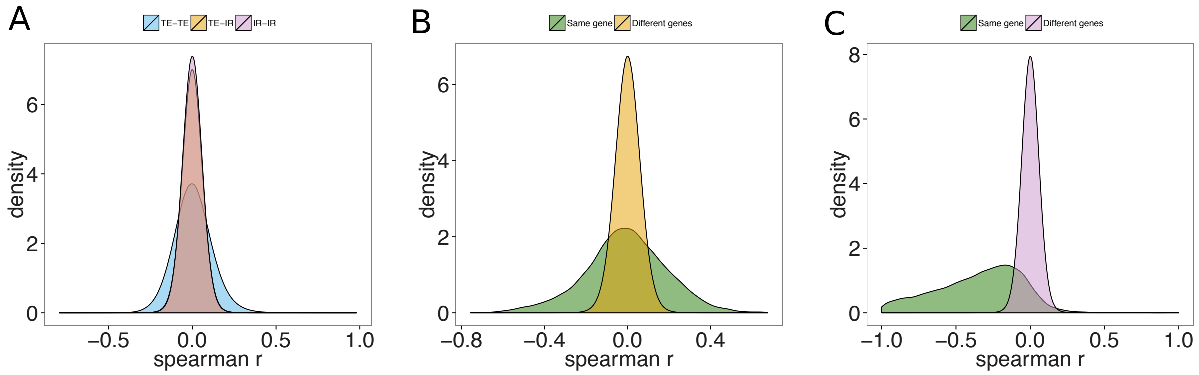

Figure 2. Empirical correlation between nodes of each type A) We computed Spearman correlation between each pair of nodes, considering only nodes belonging to distinct genes. This plot shows the distribution of the observed correlations in *whole blood*, divided into 3 categories: between two total expression nodes (TE-TE), between a total expression and an isoform ratio (TE-IR), and between two isoform ratios (IR-IR). Empirical correlation distributions were different for different categories. B) The distribution of Spearman correlation between a total expression node and an isoform ratio node, categorized by whether both they belong to the same gene or not. Total expression and isoform of the same gene were more correlated than those of different genes. C) The distribution of Spearman correlation between a pair of isoform ratio nodes, categorized by whether both isoforms belong to the same gene or not. Isoforms of the same gene were more correlated than those of different genes.

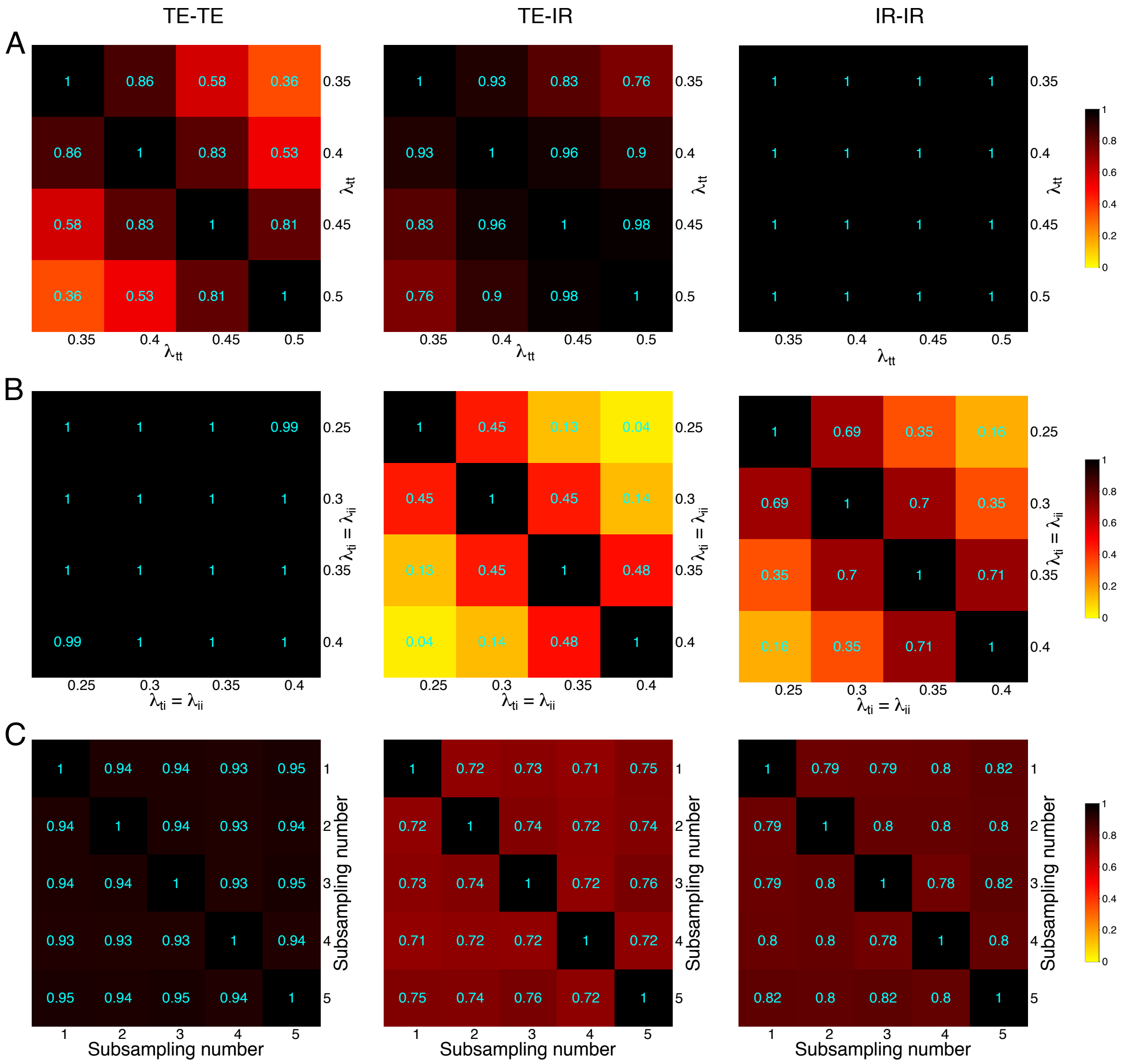

Figure 3. Robustness of TWN estimation for varying regularization parameters and sample size. We varied one of three variables at a time (regularization parameter $\lambda_{tt}, regularizationparameter\lambda ti$, or sample size) keeping other variables the same as actually used, and re-estimated TWNs in *whole blood*. We then computed Tanimoto coefficients between edge weights of every pair of re-estimated TWNs categorized by type of edge: edge between two total expression nodes (TE-TE), between a total expression and an isoform node (TE-IR), and between two isoform nodes(IR-IR). A) Tanimoto coefficients for varying λ_{tt}. Tanimoto coefficients between the selected $\lambda_{tt}(0.4)$ and nearby choices $(0.35, 0.4)$ are very high in each category (0.86 and 0.83 for TE-TE, 0.93 and 0.96 for TE-IR, 1 and 1 for IR-IR, respectively). B) Tanimoto coefficients for varying $\lambda_{ti} = \lambda_{ii}$. Here, the selected $\lambda_{ti} = \lambda_{ii}$ was 0.25. C) Tanimoto coefficients for varying sample size. In each run, we randomly selected 90 % samples and re-estimated TWNs using the regularization parameters fixed to the same as actually used.

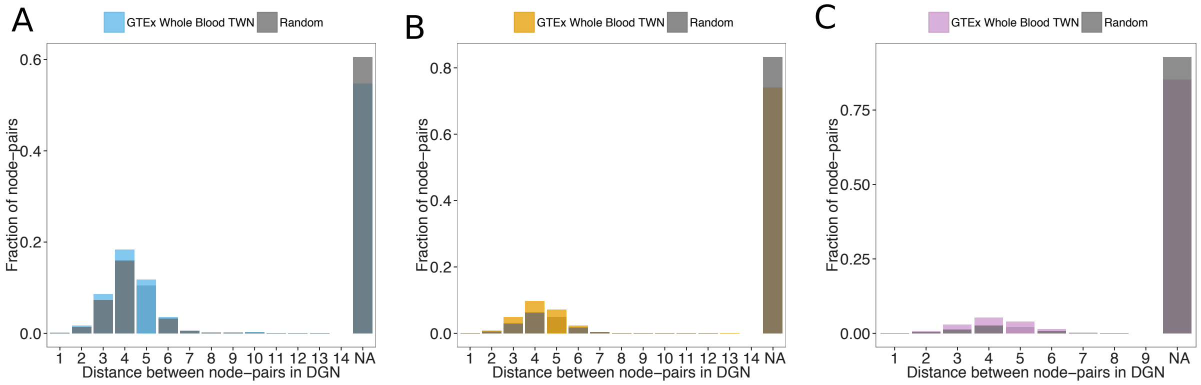

Figure 4. Replication of networks in DGN. We estimated a TWN for whole blood using the DGN data, taking genes and isoforms common in both DGN and GTEx. We computed the fraction of connected node pairs from the GTEx TWN with a given distance between them in DGN TWN, categorized by node types: two total expression nodes (A); a total expression node and an isoform ratio node (B); two isoform ratio nodes (C). For every category, the whole blood TWN constructed using GTEx data has a higher fraction of connected node pairs in DGN, compared to a random network generated by permuting total expression and isoform ratio labels.

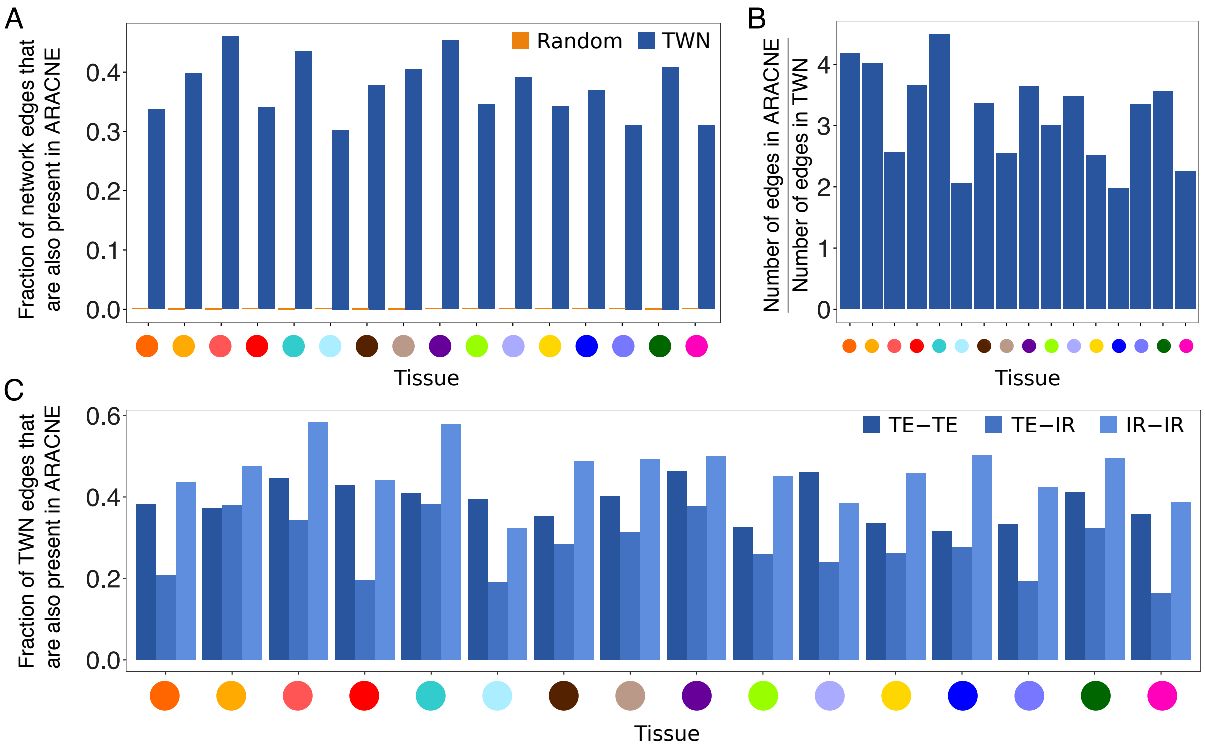

Figure 5. Replication of TWN using ARACNE. With the same data as used for TWNs, we reconstructed ARACNE networks from Spearman correlation based mutual information matrix using minet R package for 16 tissues. Following similar procedures as TWNs, we excluded edges between features of same gene, cross-mappable genes, and position-overlapped genes from downstream analysis. A) For TWNs and random networks, fraction of edges (y-axis) that were also present in ARACNE network in matched tissue (x-axis). A high fraction of TWN edges (30.42-46.34%, mean 37.72%), compared to random edges, were captured by ARACNE, demonstrating replication of TWN relationships using an independent method. B) Ratio of the number of edges in ARACNE network to the number of edges in TWN (y-axis) of the matched tissue (x-axis). On average, each ARACNE network had 3.17 times as many edges as the matched TWN indicating that TWN potentially captures direct relationships. C) Fraction of TWN edges (y-axis) that were also present in ARACNE network in matched tissue (x-axis), categorized by edge types. On average, 38.70%, 27.48%, and 46.43% of TE–TE, TE–IR, and IR–IR edges, respectively, were captured by ARACNE.

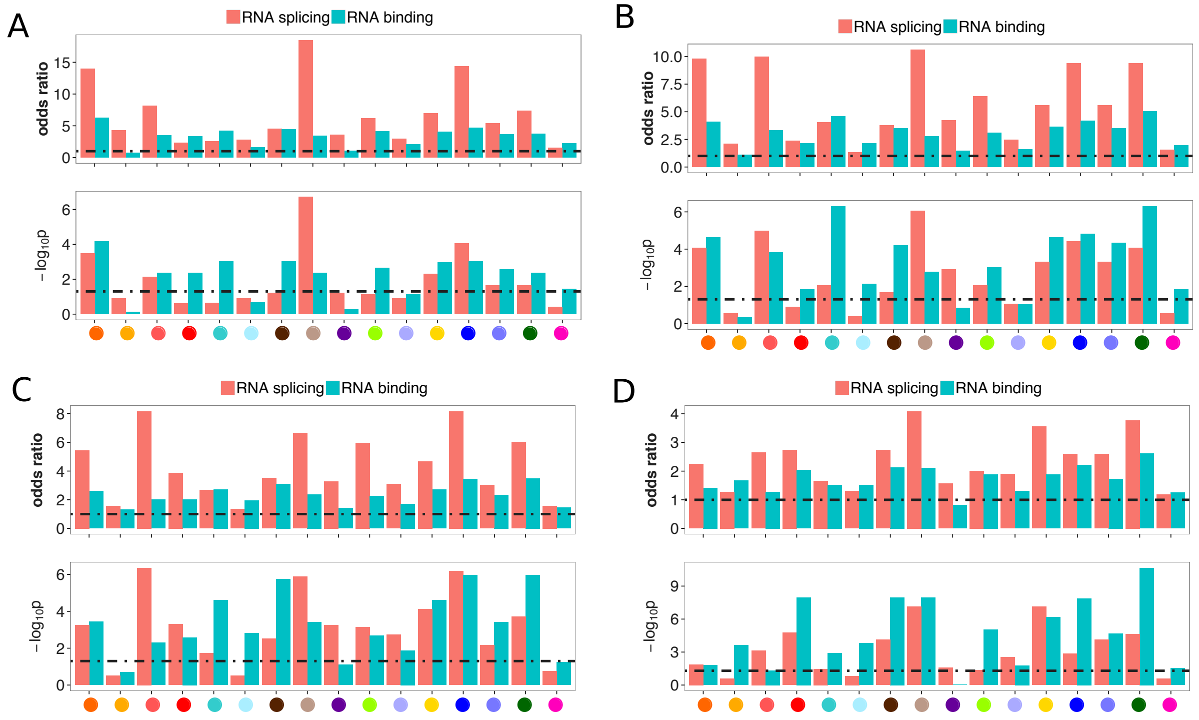

Figure 6. TWN GO enrichment for varying threshold of hub degree. We tested enrichment of RNA splicing and RNA biding genes in top N TE-IR hubs using Fisher's exact test. Here we report enrichment results for different values of N: 50 (A), 100 (B), 200 (C), and 1000 (D). P-values are BH corrected

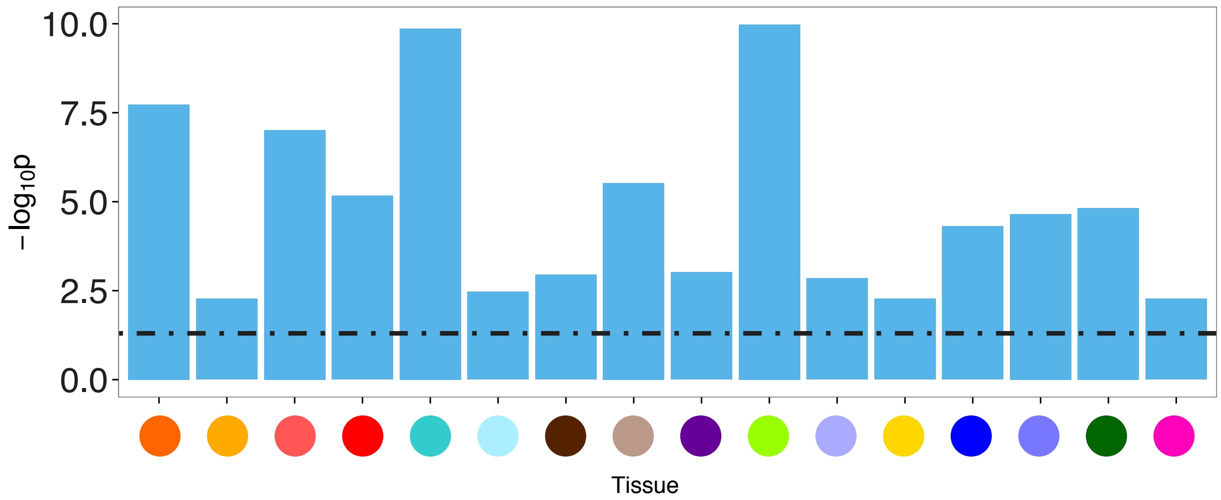

Figure 7. TWN TF-target enrichment. We measured enrichment for edges between total expression nodes representing TFs, and total expression nodes of their known targets. P-values shown are BH corrected.

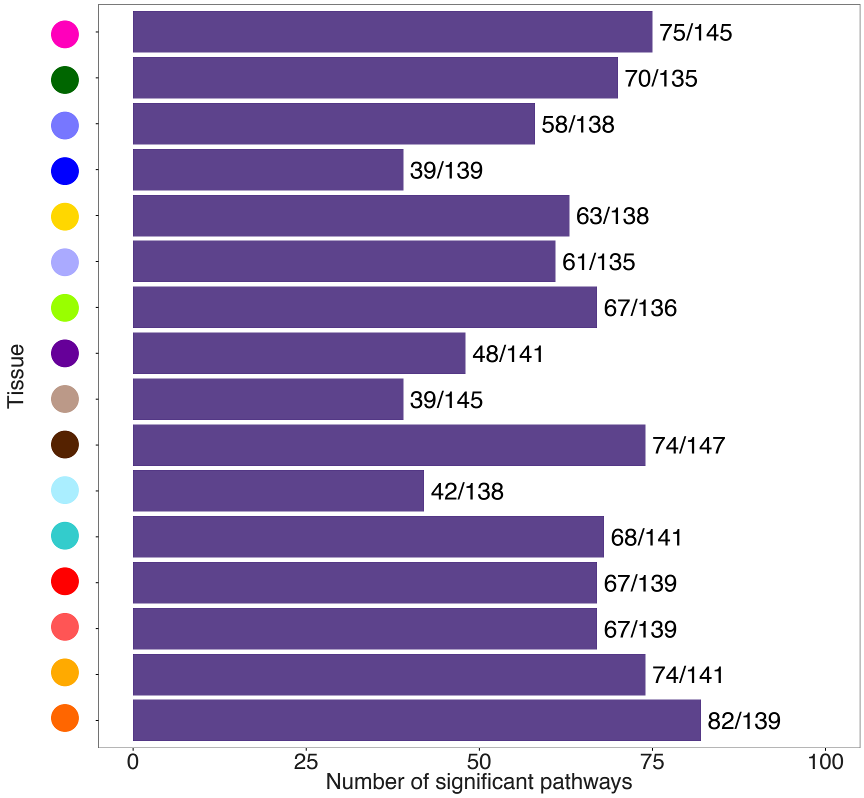

Figure 8. KEGG pathway enrichment. Per-tissue, the number of KEGG pathways enriched among connected components / total number of tested pathways for that tissue, considering only total expression nodes.

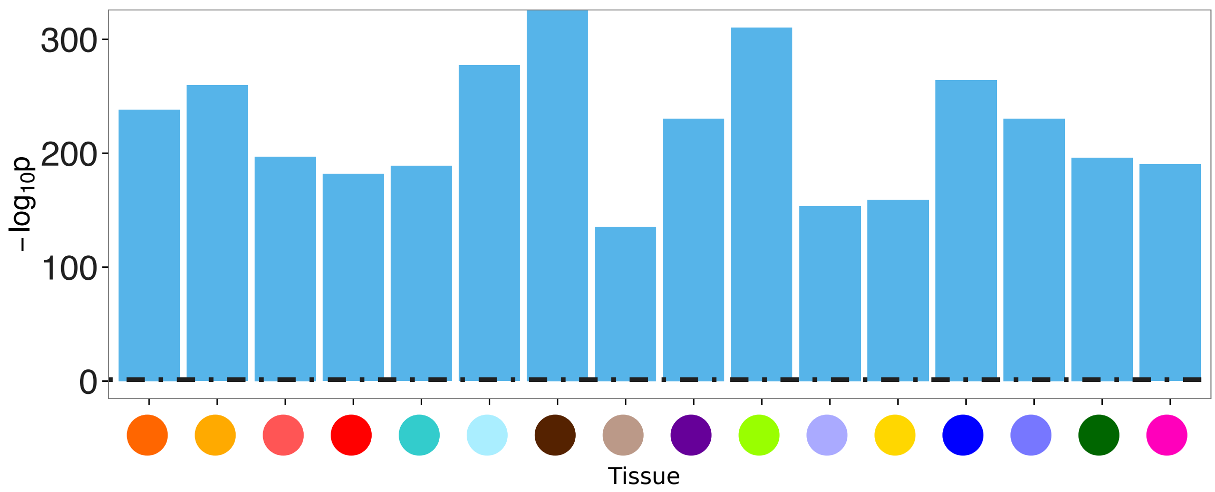

Figure 9. TWN Total expression pathway enrichment. Enrichment for shared Reactome pathway annotations among gene pairs connected by edges between two total expression nodes. P-values shown are BH corrected.

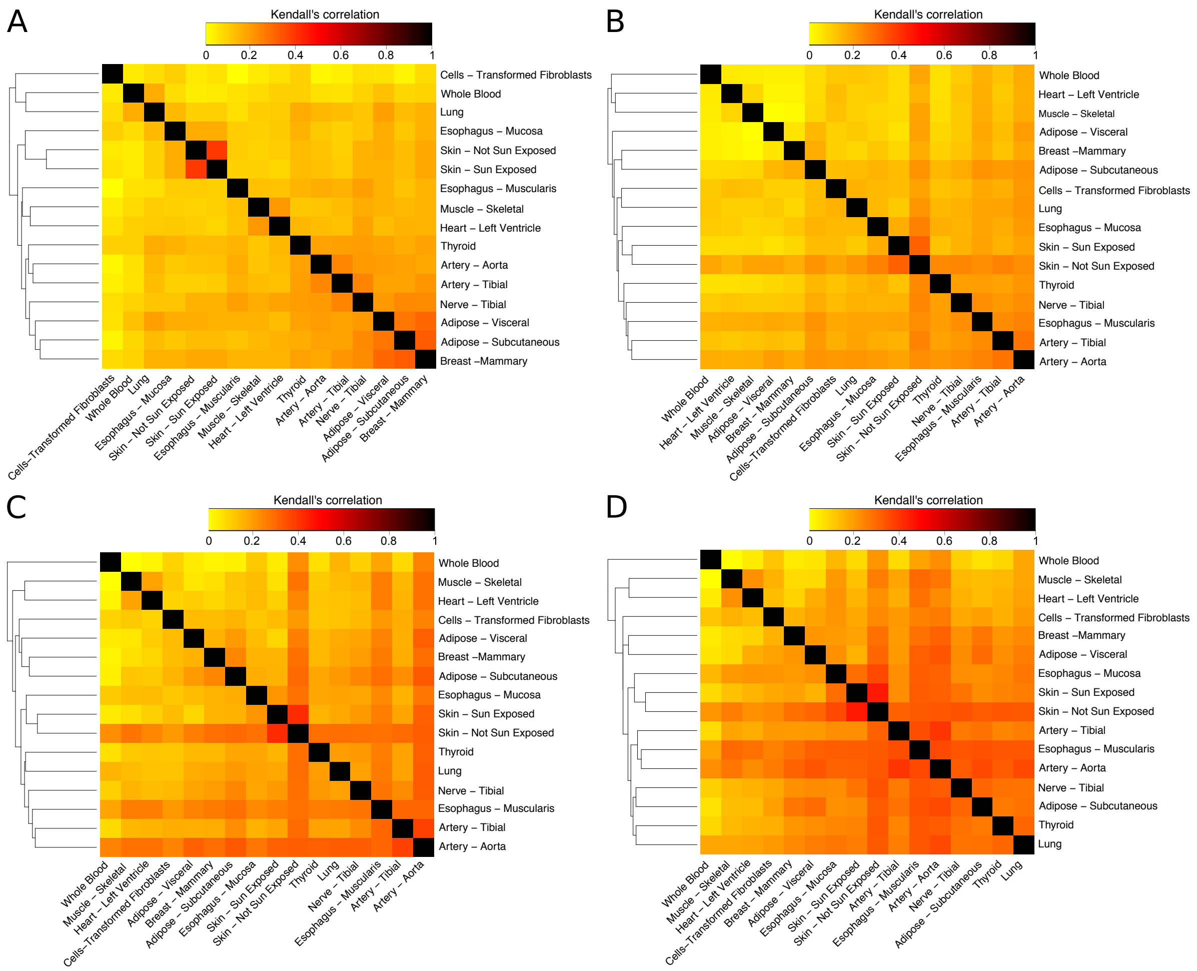

Figure 10. TWN Hub concordance. Heatmaps here show Kendall's correlation coefficients between tissue pairs using ranking of TE-TE (A), TE-IR (B), IR-TE (C), and IR-IR(D) hubs. Tissue clustering dendrograms are shown at the left side of heatmaps. Here, related tissues tend to cluster together. For example, both skin tissues cluster together in every heatmap. *Skeletal muscle* and *heart – left ventricle* tissues also cluster together.

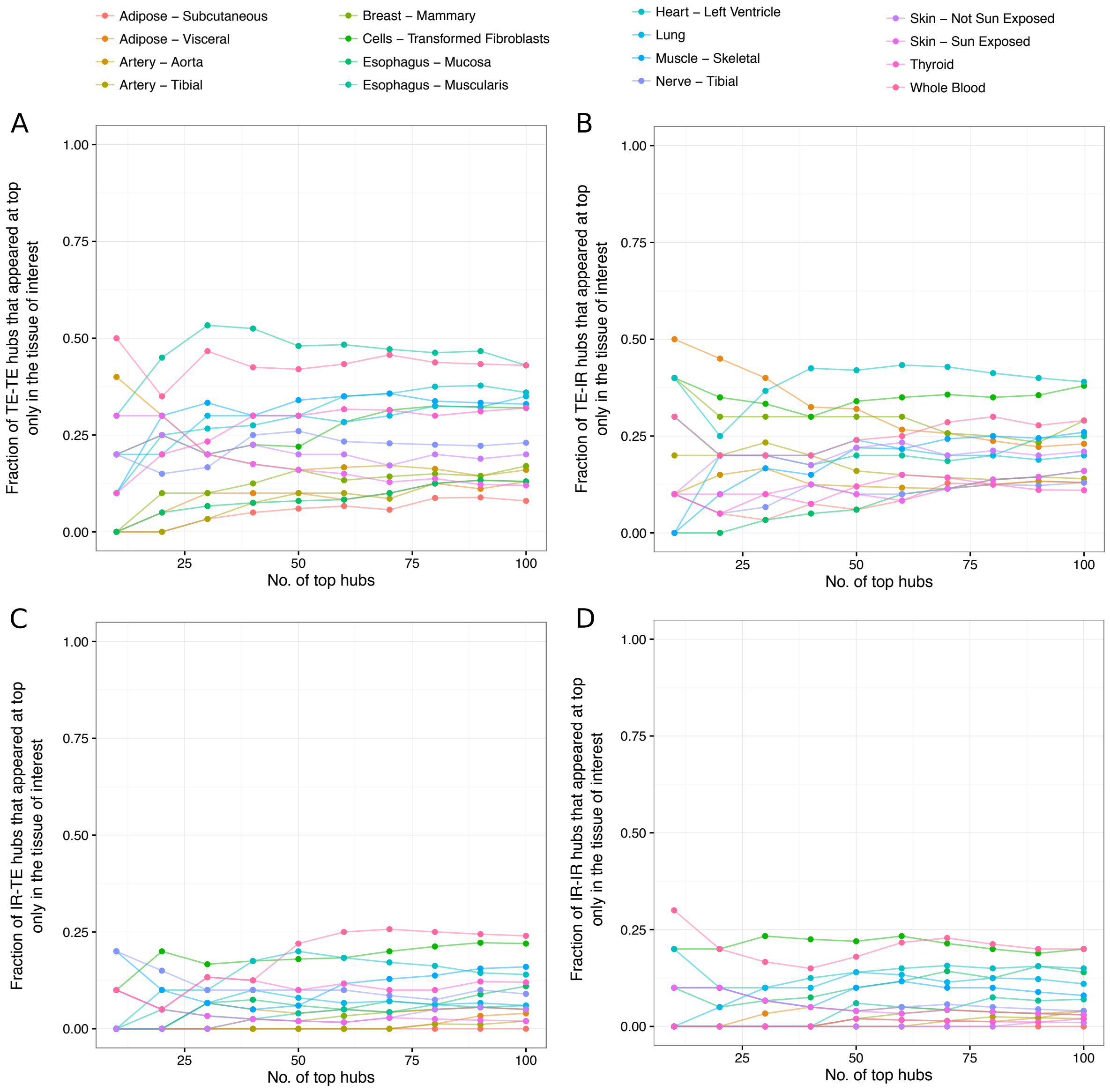

Figure 11. Proportion of tissue-specific TWN hubs. Here we consider a hub is tissue-specific if it is not present in top 500 hubs of any other tissues. This plot shows the fraction of tissue-specific TE-TE(A), TE-IR(B), IR-TE(C), and IR-IR(D) hubs in top hubs of each tissue.

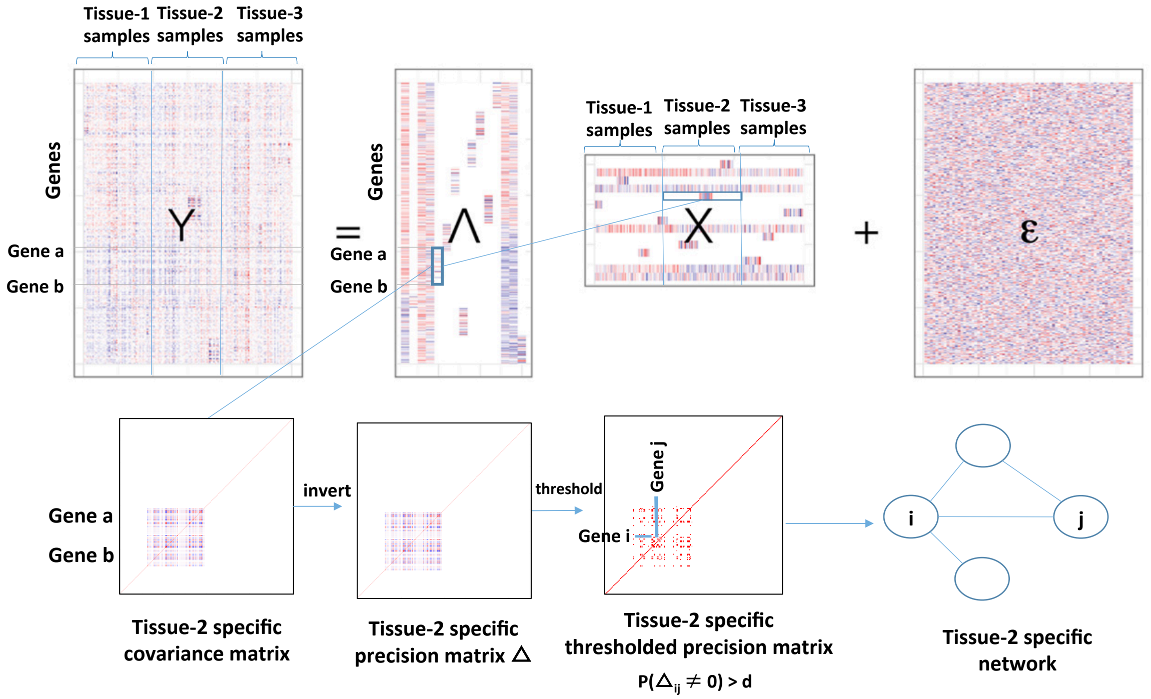

Figure 12. Tissue-specific network (TSN) conceptual framework. BicMix, a Bayesian sparse factor analysis based model, decomposes a gene-by-sample expression matrix (Y) into a gene-by-K loading matrix (Λ), a K-by-sample factor matrix (X), and a gene-by-sample residual matrix (ϵ), where K is the number of latent factors. BicMix induces sparsity in both Λ and X, and thus identifies clusters of co-expressed genes that are co-expressed in a subset of samples. Using the gene loadings corresponding only to factors with non-zero values in a single tissue, a precision submatrix (Δ) corresponding to the non-zero genes can be estimated; standardized, these values correspond to partial correlation. Thresholding these partial correlations using FDR, non-zero values correspond to an edge between a pair of genes in the tissue-specific gene co-expression network. By estimating the gene covariance matrix using only components with non-zero values among the tissue of interest in BicMix, we explicitly remove all covariation that is found outside of the tissue of interest. This shared covariation may also include covariation due to batch effects, population effects, cross-tissue expression QTLs, or cellular housekeeping pathways; while this shared variation is captured in the BicMix model, it is ignored when building the TSNs.

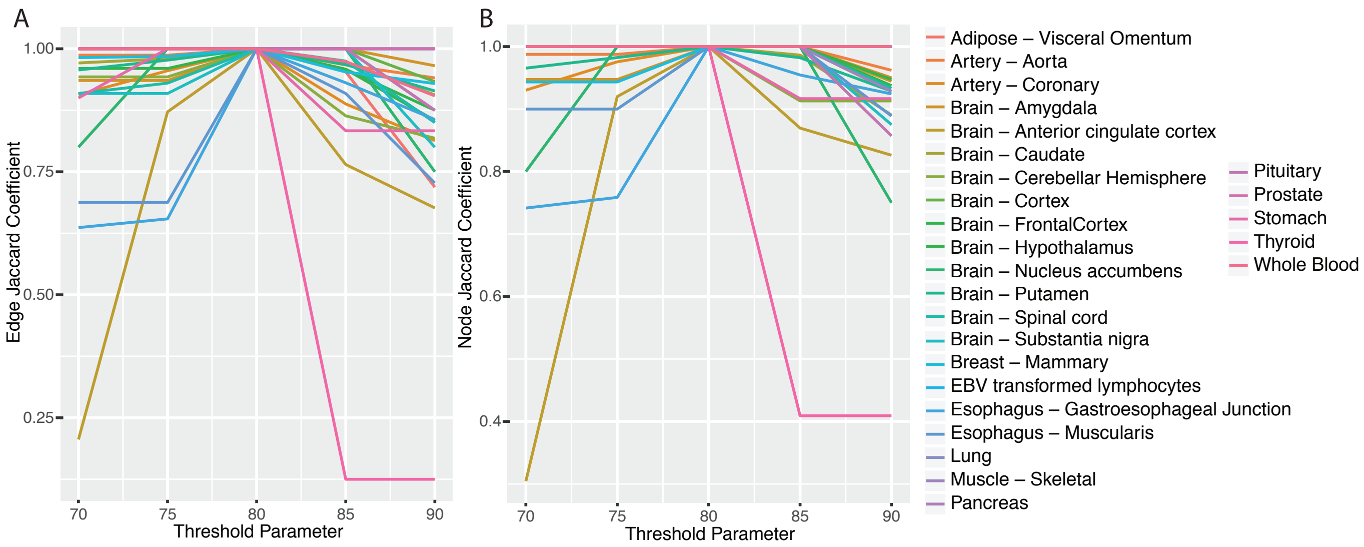

Figure 13. Majority of TSNs are robust to varying the GeneNet probability threshold for edge selection. The plots show the Jaccard coefficient (y-axis) for edges (A) and nodes (B) across all TSNs for various probability thresholds (x-axis) used to select significant edges from GeneNet.

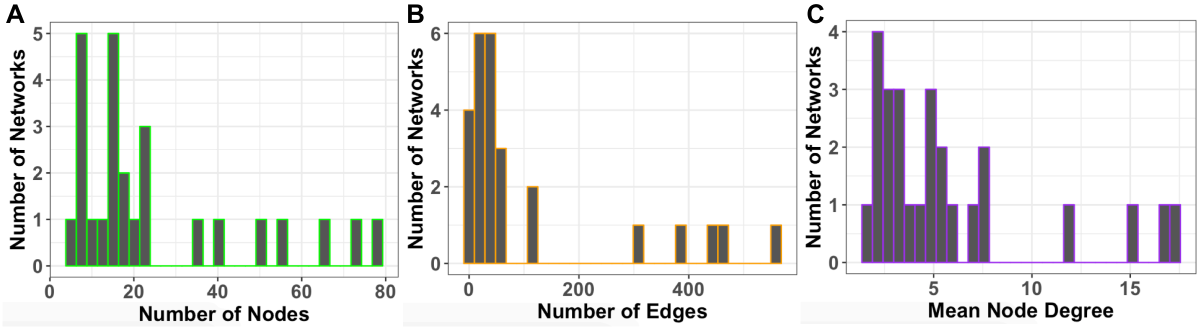

Figure 14. Summary statistics across the 26 TSNs. (A) Histogram of number of network nodes across the TSNs; (B) histogram of number of edges across the TSNs; (C) histogram of node degree across the TSNs.

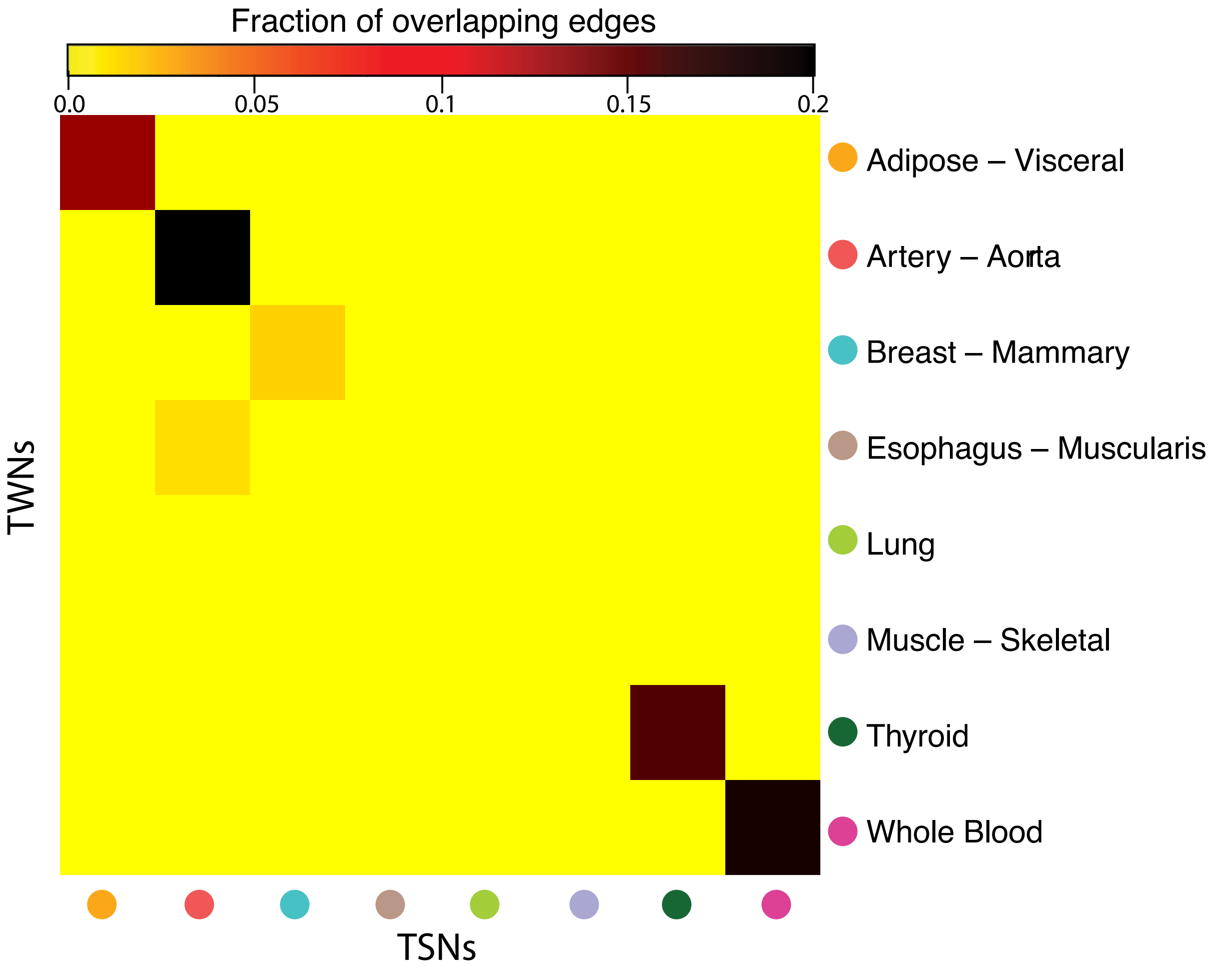

Figure 15. Replication of edges across TWNs and TSNs. Heatmap shows the fraction of edges that replicate across across TWNs and TSNs, where the discovery edges are from TWNs on the rows (i.e., lower triangular) and the discovery edges are from TSNs on the columns (i.e., upper triangular) for each of eight tissues where both were constructed.

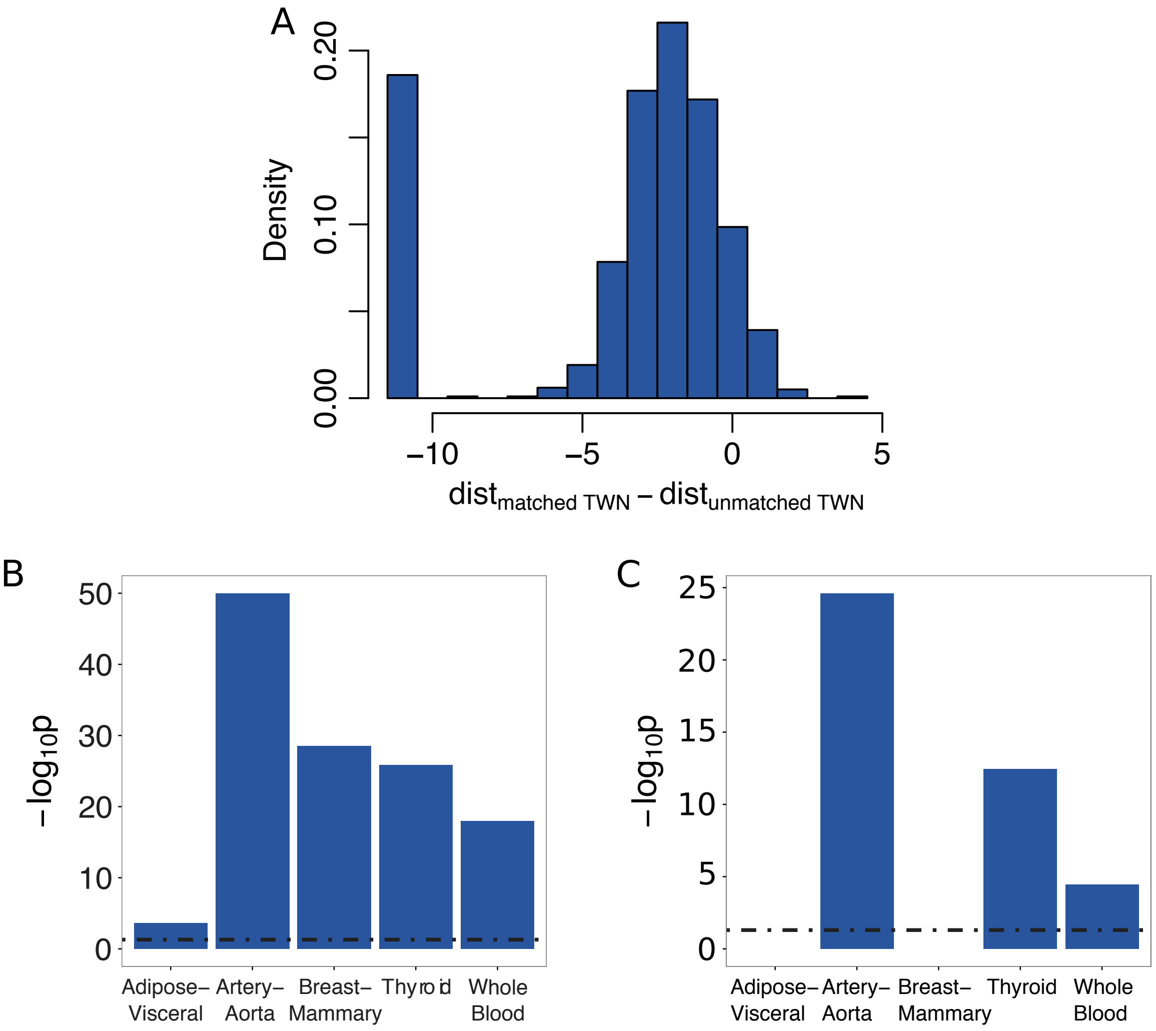

Figure 16. TSN edges were supported by TWNs and ARACNE networks. We selected edges from a TSN where both genes connected by an edge were jointly included in the matched TWN and at least another unmatched TWN. Then we tested if the path length between the nodes of a selected edge was significantly smaller in the matched TWN than in an unmatched TWN using one-sided Wilcoxon signed-rank test. A) Histogram of node distance differences for *artery-aorta*. As expected, most of the differences were negative, meaning that the nodes connected in *artery-aorta* TSN were closer to each other in *artery-aorta* TWN compared to other tissues' TWNs. B) BH corrected p-values for each tissue. Y-axis has been truncated to have a maximum value of 50. Note: a small number of edges (≤ 3) were selected for *lung*, *skeletal muscle*, and *esophagus–muscularis*, and p-values for these tissues were not reported. C) BH corrected p-values for the same test when ARACNE networks were used instead of TWNs.

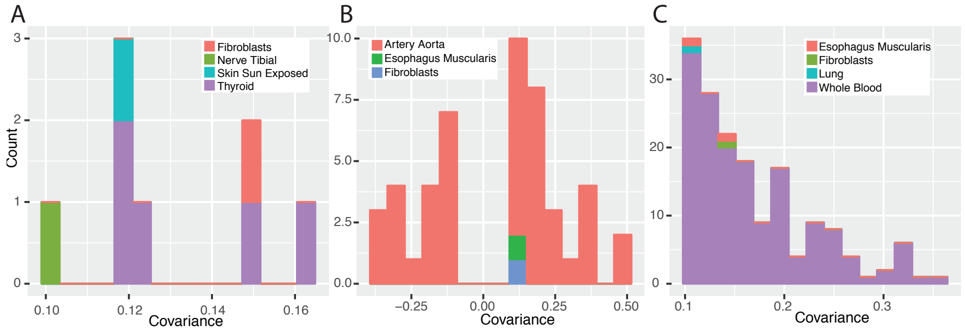

Figure 17. Shared tissue-specific structure between TSNs and TWNs. Histograms show the distribution of estimated covariance values larger than 0.1 (x-axis) in TWNs (legend) between genes connected by an edge in the thyroid (A), artery – aorta (B), and whole blood (C) TSNs. In each histogram, edges from the corresponding tissue's TWN are enriched.

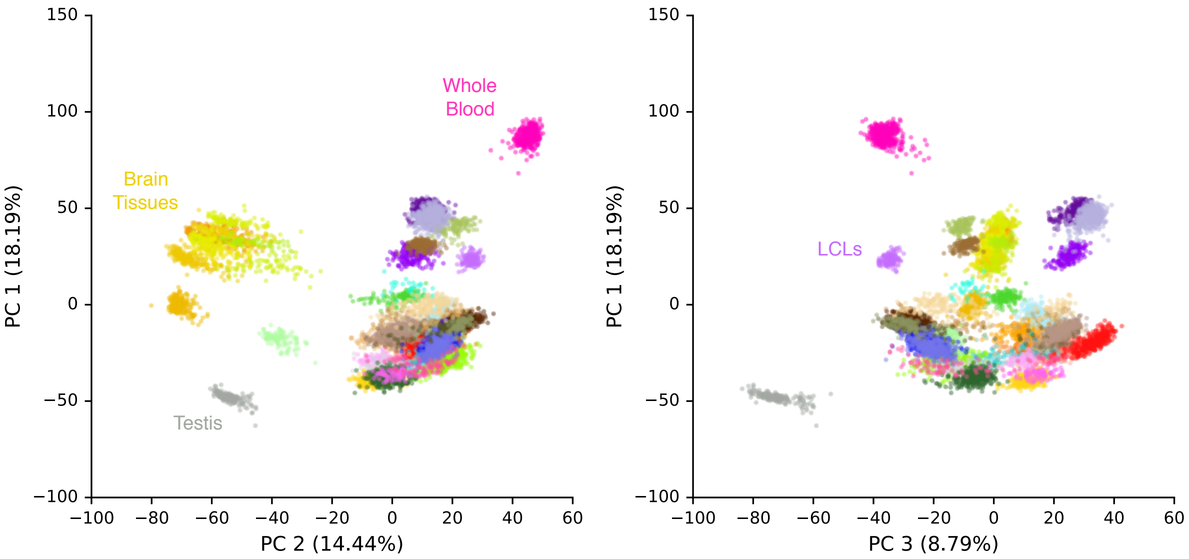

Figure 18. Principal components of gene expression levels across samples from the 44 GTEx v6 tissues. (A) Principal component 1 (PC1), explaining 18.1% of the variance in gene expression levels across tissues, versus PC2, explaining 14.4% of the variance in gene expression levels across tissues; (B) PC1 versus PC3, explaining 8.8% of the variance in gene expression levels across tissues.

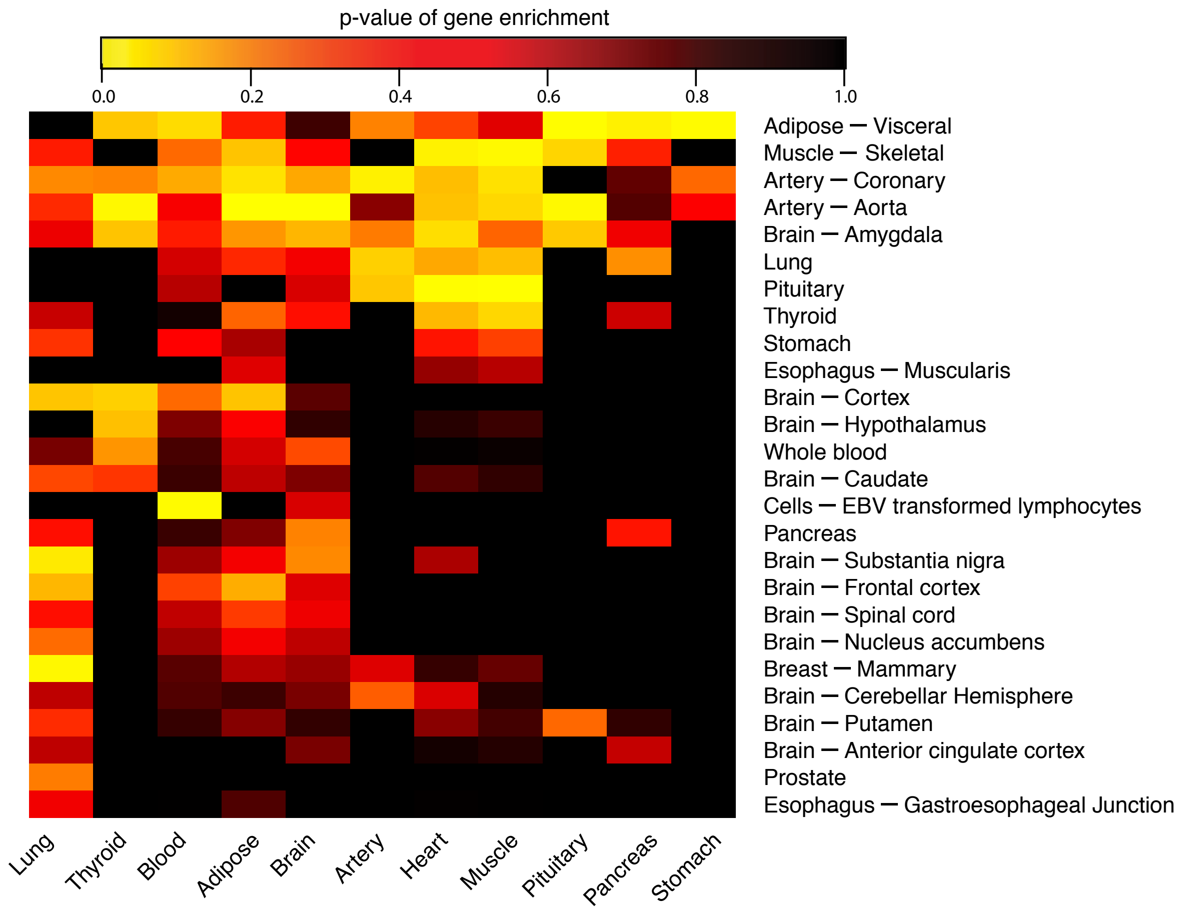

Figure 19. P-value enrichment from Fisher's exact test of genes with tissue-specific function in TSNs. Heatmap shows p-values for the tissue-specific gene function (x-axis) enrichment in the TSN networks (y-axis).

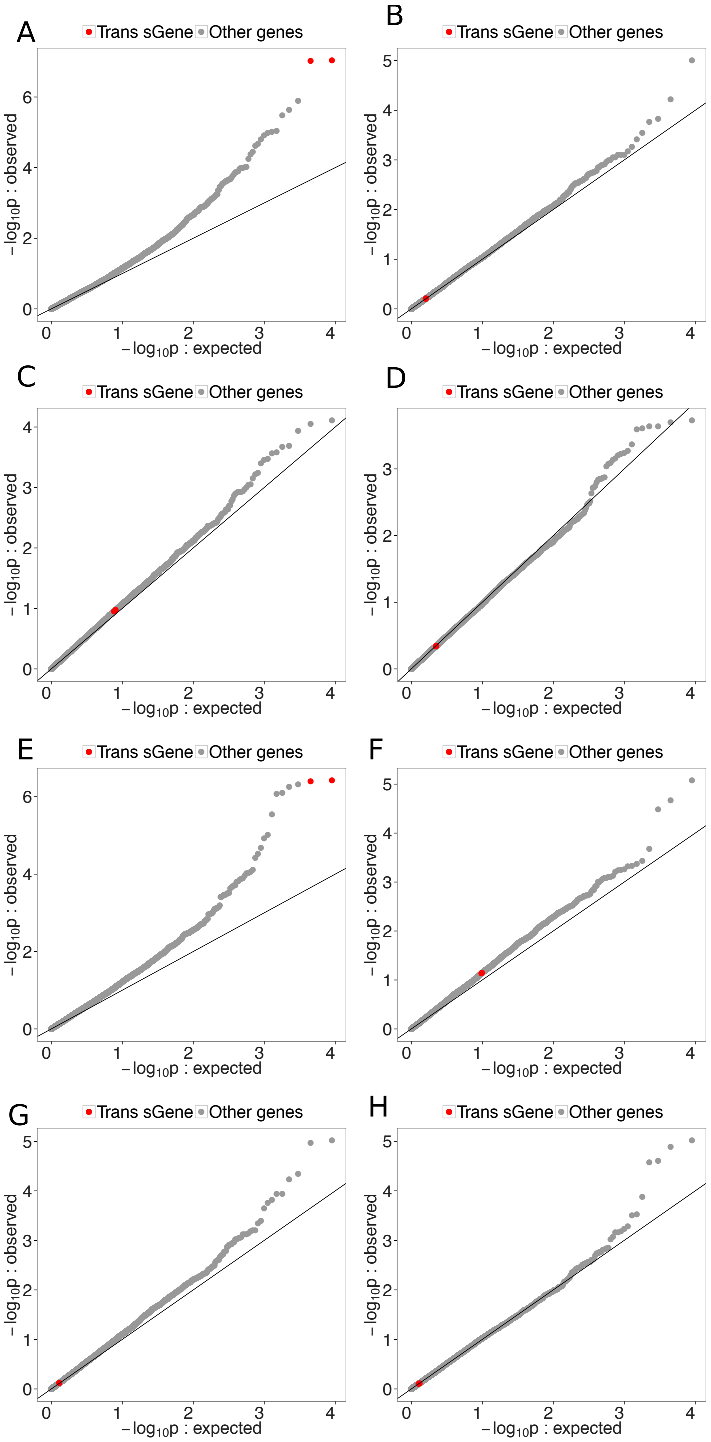

Figure 20. Association of rs113305055 and rs59153288 with distal isoform ratio across multiple tissues. We measured association for each variant with all isoform ratios genome-wide, and plotted observed p-values against uniformly distributed expected p-values. Top four plots shows enrichment of rs113305055 in *artery – tibial* (A), *whole blood* (B), *skeletal muscle* (C), and *thyroid* (D). Bottom four plots show enrichment of rs59153288 in *breast – mammary* (E), *artery – aorta* (F), *whole blood* (G), and *skin – not sun exposed* (H).

Supplemental Tables

**Tissue**	λ_{tt}	λ_{te}	λ_{ii}
Adipose – Subcutaneous	0.4	0.25	0.25
Adipose – Visceral	0.4	0.3	0.3
Artery – Aorta	0.4	0.25	0.25
Artery – Tibial	0.4	0.25	0.25
Breast – Mammary	0.4	0.3	0.3
Esophagus – Mucosa	0.4	0.25	0.25
Esophagus – Muscularis	0.4	0.25	0.25
Cells – Transformed Fibroblasts	0.4	0.25	0.25
Heart – Left Ventricle	0.4	0.3	0.3
Lung	0.4	0.25	0.25
Nerve – Tibial	0.35	0.25	0.25
Muscle – Skeletal	0.35	0.25	0.25
Skin – Not Sun Exposed	0.4	0.25	0.25
Skin – Sun Exposed	0.4	0.25	0.25
Thyroid	0.35	0.25	0.25
Whole Blood	0.4	0.25	0.25

Table 1. Selected penalty parameters for graphical lasso.

Tissue	Hub Type	Top five hub genes with number of neighboring genes.
Muscle – Skeletal	TE-TE	1:*C1orf198*(70) 2:*MAN1A1*(69) 3:*CD63*(69) 4:*MYH14*(68) 5:*MLLT1*(65)
Muscle – Skeletal	TE-IR	1:*NCOA5*(36) 2:*SAP18*(32) 3:*ZBTB40*(21) 4:*MYL3*(21) 5:*CLPB*(19)
Muscle – Skeletal	IR-TE	1:*MYBPC1*(247) 2:*UGP2*(111) 3:*FBXO32*(106) 4:*IQSEC2*(105) 5:*HHATL*(96)
Muscle – Skeletal	IR-IR	1:*ACADVL*(112) 2:*TSPYL2*(100) 3:*RBM24*(97) 4:*TMUB1*(95) 5:*PINK1*(93)
Whole Blood	TE-TE	1:*MAP3K2*(72) 2:*ENTPD7*(69) 3:*GALNT2*(69) 4:*SNX17*(69) 5:*TWF2*(62)
Whole Blood	TE-IR	1:*CRY1*(34) 2:*C11orf89*(32) 3:*ARRDC3*(32) 4:*RMI1*(32) 5:*C5orf58*(29)
Whole Blood	IR-TE	1:*CCND3*(292) 2:*MSL1*(228) 3:*SLC3A2*(166) 4:*DNM2*(130) 5:*WIPF1*(127)
Whole Blood	IR-IR	1:*WIPF1*(106) 2:*SLC6A6*(105) 3:*ZCCHC6*(102) 4:*CPD*(101) 5:*SMCHD1*(97)
Skin – Sun Exposed	TE-TE	1:*ACVR1B*(77) 2:*HES1*(73) 3:*E2F4*(66) 4:*ARMC6*(65) 5:*BHLHE40*(64)
Skin – Sun Exposed	TE-IR	1:*C16orf58*(34) 2:*C20orf24*(27) 3:*NDUFAF3*(26) 4:*RBM14*(24) 5:*DIDO1*(23)
Skin – Sun Exposed	IR-TE	1:*EDF1*(185) 2:*SYNGR1*(171) 3:*DDX39A*(141) 4:*SPIDR*(126) 5:*CTSF*(126)
Skin – Sun Exposed	IR-IR	1:*IGFBP3*(99) 2:*MRPS34*(96) 3:*DIAPH1*(94) 4:*TMUB1*(85) 5:*CASC3*(84)
Adipose – Subcutaneous	TE-TE	1:*CDH5*(62) 2:*CYYR1*(62) 3:*TSPAN7*(61) 4:*KCNH2*(60) 5:*DCN*(56)
Adipose – Subcutaneous	TE-IR	1:*TMEM160*(43) 2:*ARGLU1*(36) 3:*ZRANB2*(35) 4:*FOXRED1*(28) 5:*RBM14*(27)
Adipose – Subcutaneous	IR-TE	1:*FBLN2*(148) 2:*CENPV*(140) 3:*FXYD1*(127) 4:*HDAC9*(125) 5:*SPTAN1*(122)
Adipose – Subcutaneous	IR-IR	1:*IGFBP3*(136) 2:*MXRA8*(113) 3:*IQGAP1*(110) 4:*DUT*(103) 5:*PPM1G*(84)
Artery – Tibial	TE-TE	1:*RPRD2*(108) 2:*MLLT1*(72) 3:*CENPT*(66) 4:*SF3A1*(64) 5:*KCNH2*(62)
Artery – Tibial	TE-IR	1:*ZBTB21*(33) 2:*PSMA4*(32) 3:*ZNF692*(29) 4:*ZBTB10*(28) 5:*NDEL1*(26)
Artery – Tibial	IR-TE	1:*SRCAP*(186) 2:*NR4A2*(167) 3:*RARA*(159) 4:*SESN1*(152) 5:*SYNPO2*(142)
Artery – Tibial	IR-IR	1:*IGFBP3*(130) 2:*TSPYL2*(96) 3:*PALLD*(88) 4:*MXRA8*(83) 5:*HYI*(81)
Thyroid	TE-TE	1:*NOD1*(79) 2:*ZNF853*(77) 3:*SF1*(76) 4:*RAS-GRP2*(76) 5:*ZNF692*(75)
Thyroid	TE-IR	1:*PNN*(40) 2:*MGEA5*(30) 3:*ARGLU1*(29) 4:*SRSF11*(28) 5:*XPO1*(25)
Thyroid	IR-TE	1:*MAPKAPK3*(175) 2:*RAP1GAP*(101) 3:*SRSF1*(96) 4:*SLC3A2*(90) 5:*LARP6*(86)
Thyroid	IR-IR	1:*C7orf43*(100) 2:*MXRA8*(93) 3:*RILP*(92) 4:*ZNF580*(84) 5:*TMUB1*(83)

Table 2 – continued from previous page

Tissue	Hub Type	Top five hub genes with number of neighboring genes
Lung	TE-TE	1:*LAPTM5*(65) 2:*SLC19A2*(64) 3:*TSPAN13*(61) 4:*SMAP2*(59) 5:*ASF1B*(59)
Lung	TE-IR	1:*C5orf54*(36) 2:*PTMS*(35) 3:*AMPD2*(33) 4:*DAPK3*(32) 5:*RBM14*(32)
Lung	IR-TE	1:*SERPINA1*(140) 2:*TXNDC5*(123) 3:*CD47*(123) 4:*DENND1C*(108) 5:*CCND3*(106)
Lung	IR-IR	1:*CYTH1*(113) 2:*TSPYL2*(110) 3:*CLK1*(105) 4:*SEC61A1*(104) 5:*MXRA8*(98)
Nerve – Tibial	TE-TE	1:*SLC2A1*(87) 2:*ESAM*(82) 3:*PDZRN4*(78) 4:*MBD3*(77) 5:*CPXM2*(77)
Nerve – Tibial	TE-IR	1:*CMTR2*(46) 2:*MGEA5*(42) 3:*HEMK1*(34) 4:*XPO1*(34) 5:*PGAP2*(32)
Nerve – Tibial	IR-TE	1:*PCOLCE*(189) 2:*FXYD1*(175) 3:*ZNF536*(128) 4:*QTRT1*(119) 5:*SLC3A2*(117)
Nerve – Tibial	IR-IR	1:*MXRA8*(149) 2:*CAPZB*(115) 3:*LMNA*(105) 4:*ATOH8*(102) 5:*NUCB1*(102)
Esophagus – Mucosa	TE-TE	1:*CIRBP*(70) 2:*CACNA1H*(64) 3:*RBPMS2*(63) 4:*IGFBP4*(60) 5:*CLDN3*(59)
Esophagus – Mucosa	TE-IR	1:*ODC1*(41) 2:*SLC6A6*(33) 3:*UNC119B*(32) 4:*MTPN*(30) 5:*ZMAT1*(28)
Esophagus – Mucosa	IR-TE	1:*BUB3*(190) 2:*TIMP2*(138) 3:*SRCAP*(114) 4:*ATP2B4*(104) 5:*THRA*(101)
Esophagus – Mucosa	IR-IR	1:*CLINT1*(113) 2:*TMPRSS11D*(103) 3:*LMO7*(96) 4:*ITM2B*(96) 5:*APP*(96)
Cells – Transformed Fibroblasts	TE-TE	1:*GABBR1*(79) 2:*PNISR*(78) 3:*PDGFD*(74) 4:*SVEP1*(69) 5:*DMTF1*(68)
Cells – Transformed Fibroblasts	TE-IR	1:*ZBTB9*(40) 2:*IGIP*(39) 3:*LRIF1*(39) 4:*HNRN-PLL*(35) 5:*CHPF2*(32)
Cells – Transformed Fibroblasts	IR-TE	1:*ANTXR1*(309) 2:*AOX1*(191) 3:*HDAC9*(186) 4:*KIF22*(180) 5:*CDKN2C*(178)
Cells – Transformed Fibroblasts	IR-IR	1:*MMP2*(230) 2:*MXRA8*(160) 3:*ITGAV*(151) 4:*FSTL1*(151) 5:*FLNA*(144)
Skin – Not Sun Exposed	TE-TE	1:*APMAP*(72) 2:*KIF5B*(71) 3:*MAOA*(70) 4:*RORA*(64) 5:*SGK2*(62)
Skin – Not Sun Exposed	TE-IR	1:*YKT6*(53) 2:*RBM14*(51) 3:*RGP1*(47) 4:*CBR4*(44) 5:*MASP2*(44)
Skin – Not Sun Exposed	IR-TE	1:*PCOLCE*(171) 2:*ECM1*(133) 3:*PCDH7*(126) 4:*SYNGR1*(124) 5:*GSN*(118)
Skin – Not Sun Exposed	IR-IR	1:*SEC61A1*(128) 2:*DDRGK1*(127) 3:*SRSF7*(118) 4:*WDR33*(115) 5:*CREBZF*(115)
Esophagus – Muscularis	TE-TE	1:*C20orf27*(78) 2:*TCF7L1*(77) 3:*PALM*(64) 4:*ISOC2*(63) 5:*ODC1*(62)
Esophagus – Muscularis	TE-IR	1:*SRRM2*(44) 2:*PTAR1*(30) 3:*RHOT2*(29) 4:*ZNF692*(29) 5:*RBM5*(28)
Esophagus – Muscularis	IR-TE	1:*SMTN*(298) 2:*PLCD4*(214) 3:*GSN*(184) 4:*TSC22D4*(146) 5:*DMPK*(142)

Table 2 – continued from previous page

Tissue	Hub Type	Top five hub genes with number of neighboring genes
Esophagus – Muscularis	IR-IR	1:*TRIO*(138) 2:*ATP5B*(129) 3:*MXRA8*(123) 4:*ITM2B*(117) 5:*HDAC7*(112)
Adipose – Visceral	TE-TE	1:*STK40*(82) 2:*KLF15*(79) 3:*IGSF9*(75) 4:*CYYR1*(73) 5:*ZBTB16*(70)
Adipose – Visceral	TE-IR	1:*FASTK*(14) 2:*USP48*(13) 3:*DNAJC27*(13) 4:*NR4A3*(12) 5:*PTPN1*(12)
Adipose – Visceral	IR-TE	1:*DPYSL3*(111) 2:*THRA*(98) 3:*CLSTN3*(75) 4:*ARRDC2*(66) 5:*ACOT8*(52)
Adipose – Visceral	IR-IR	1:*ATP2B4*(117) 2:*EPAS1*(113) 3:*IGFBP3*(104) 4:*ALDOA*(87) 5:*ANXA6*(77)
Artery – Aorta	TE-TE	1:*MAOA*(81) 2:*NFIA*(69) 3:*ZNF395*(67) 4:*HOXA4*(62) 5:*HOXB7*(61)
Artery – Aorta	TE-IR	1:*PPP1R10*(36) 2:*DAK*(36) 3:*EP300*(33) 4:*PRPF38B*(32) 5:*NCOA5*(32)
Artery – Aorta	IR-TE	1:*HOXB6*(190) 2:*SRCAP*(184) 3:*ANKRD10*(125) 4:*MKNK2*(122) 5:*POSTN*(121)
Artery – Aorta	IR-IR	1:*PPM1A*(140) 2:*MXRA8*(109) 3:*CLK1*(100) 4:*SLC19A1*(100) 5:*IGFBP3*(97)
Heart – Left Ventricle	TE-TE	1:*PTPN23*(79) 2:*FKBP3*(72) 3:*SRCAP*(68) 4:*ANKRD52*(66) 5:*SH3BGRL*(63)
Heart – Left Ventricle	TE-IR	1:*ZMAT2*(36) 2:*TMEM160*(19) 3:*MYL3*(19) 4:*CACNA2D1*(19) 5:*C20orf24*(19)
Heart – Left Ventricle	IR-TE	1:*CALD1*(99) 2:*SMTN*(92) 3:*PPP1R16A*(88) 4:*ASB1*(87) 5:*THRA*(79)
Heart – Left Ventricle	IR-IR	1:*MYOM1*(125) 2:*MYOM2*(113) 3:*ECH1*(104) 4:*SORBS1*(98) 5:*RYR2*(90)
Breast – Mammary	TE-TE	1:*MMP14*(71) 2:*WDTC1*(69) 3:*CBX7*(65) 4:*SPARCL1*(64) 5:*TCERG1*(61)
Breast – Mammary	TE-IR	1:*KRT5*(25) 2:*ODC1*(22) 3:*C5orf54*(18) 4:*SCGB2A2*(17) 5:*ZMAT1*(17)
Breast – Mammary	IR-TE	1:*LSP1*(94) 2:*EFCAB4A*(81) 3:*PATZ1*(67) 4:*THRA*(60) 5:*COL1A1*(59)
Breast – Mammary	IR-IR	1:*IGFBP3*(110) 2:*PPM1G*(80) 3:*MXRA8*(79) 4:*TMUB1*(75) 5:*CST3*(65)

Table 2. Top five hubs of each category in each tissue

GO BP id	Biological process name	Number of tissues
GO:0016070	RNA metabolic process	13
GO:0010467	gene expression	12
GO:0090304	nucleic acid metabolic process	12
GO:0044260	cellular macromolecule metabolic process	11
GO:0006396	RNA processing	9
GO:0006725	cellular aromatic compound metabolic process	8
GO:0008380	RNA splicing	8
GO:0043170	macromolecule metabolic process	8
GO:0044237	cellular metabolic process	8
GO:0046483	heterocycle metabolic process	8
GO:0006139	nucleobase-containing compound metabolic process	7
GO:0006807	nitrogen compound metabolic process	7
GO:0034645	cellular macromolecule biosynthetic process	7
GO:0044238	primary metabolic process	7
GO:0016071	mRNA metabolic process	6
GO:0034641	cellular nitrogen compound metabolic process	6
GO:0071704	organic substance metabolic process	6
GO:1901360	organic cyclic compound metabolic process	6
GO:0006397	mRNA processing	5
GO:0032774	RNA biosynthetic process	5
GO:0051252	regulation of RNA metabolic process	5
GO:2000112	regulation of cellular macromolecule biosynthetic process	5

Table 3. Top GO Biological processes among TE-IR hubs. We tested for enrichment of all GO biological processes (BPs) in top 500 TE-IR hubs. We selected a BP term in our analysis if that had at least 20 genes in our data. This table summarizes the top BP terms based on the number of tissues they appear in top 20 strongest terms (lowest p) in individual tissues.

GO MF id	Molecular function name	Number of tissues
GO:0003723	RNA binding	15
GO:0044822	poly(A) RNA binding	15
GO:0003676	nucleic acid binding	14
GO:1901363	heterocyclic compound binding	14
GO:0003677	DNA binding	13
GO:0097159	organic cyclic compound binding	13
GO:0001071	nucleic acid binding transcription factor activity	9
GO:0003700	sequence-specific DNA binding transcription factor activity	9
GO:0000975	regulatory region DNA binding	7
GO:0000989	transcription factor binding transcription factor activity	7
GO:0001067	regulatory region nucleic acid binding	7
GO:0008168	methyltransferase activity	7
GO:0044212	transcription regulatory region DNA binding	7
GO:0000981	sequence-specific DNA binding RNA polymerase II transcription factor activity	6
GO:0000988	protein binding transcription factor activity	6
GO:0003712	transcription cofactor activity	6
GO:0005488	binding	6
GO:0043565	sequence-specific DNA binding	5
GO:0043566	structure-specific DNA binding	5
GO:0003713	transcription coactivator activity	4
GO:0003714	transcription corepressor activity	4
GO:0016741	transferase activity, transferring one-carbon groups	4

Table 4. Top GO Molecular functions among TE-IR hubs. We tested for enrichment of all GO molecular functions (MFs) in top 500 TE-IR hubs. We selected a MF term in our analysis if that had at least 20 genes in our data. This table summarizes the top MF terms based on the number of tissues they appear in top 20 strongest terms (lowest p) in individual tissues.

Tissue-group	Hub type	Top 5 group-specific hub genes
Skin – Sun Exposed and Skin – Not Sun Exposed	TE-TE	*LYG2*, *FAM26D*, *MOGAT2*, *AWAT2*, *DSG4*
	TE-IR	*OTUB2*, *CCL27*, *C2CD4D*, *HMGXB4*, *SPATA5L1*
	IR-TE	*EDF1*, *ECI1*, *KRT73*, *IGFL2*, *TP53AIP1*
	IR-IR	*DSG1*, *EDF1*, *GDPD2*, *BGN*, *GCSAM*
Adipose – Subcutaneous, Adipose – Visceral, and Breast – Mammary	TE-TE	*KIAA1239*, *TTC36*, *GLIS1*, *SLAMF9*, *THSD7A*
	TE-IR	*TTC36*, *DNAJC27*, *GLYAT*, *USP48*, *RHOXF1*
	IR-TE	*AMPH*, *CTD-3193O13.9*, *HRASLS5*, *MYRF*, *TNMD*
	IR-IR	*EPS8L1*, *GYG2*, *TNFRSF8*, *NAT8L*, *ZNF689*
Heart – Left Ventricle, and and Muscle – Skeletal	TE-TE	*CLCN4, ASB14, DHRS7C, ANKRD52, LRRC14B*
	TE-IR	*XPO4, LMOD2, C10orf71, RP11-766F14.2, NMRK2*
	IR-TE	*TTN, PPAP2A, FRMD3, PHC2, HFE2*
	IR-IR	*XIRP1, TBC1D4, UNC45B, MYOM3, NDUFS6*
Esophagus – Mucosa, and Esophagus – Muscularis	TE-TE	CHRNA3, *INA, ADRB3, CPLX2, SYNGR3*
	TE-IR	*CWH43, COMMD7, ZNF584, IQSEC2, CXCL11*
	IR-TE	*TMPRSS11D, TACR2, ARHGEF18, DUOXA1, PCSK5*
	IR-IR	*TMPRSS11D, ADH7, SIM2, C18orf25, PAX9*
Artery – Aorta Artery – Tibial	TE-TE	*NFIA, PKD2L1, CHD1L, FOXD1, SLC30A3*
	TE-IR	*FAF2, FAM26E, C18orf21, ZC3HC1, BDNF*
	IR-TE	*TRIM36, ATRNL1, PXDN, ETS2, SEMA4F*
	IR-IR	*TRIM36, H2AFJ, ATRNL1, PARD3B, SEMA4F*

Table 5. Top 5 tissue-specific hubs for each tissue-group

Tissue	TE–TE	TE–IR	IR-TE	IR-R
Adipose – Subcutaneous	62.50%	38.46%	NA	NA
Adipose – Visceral	53.85%	39.13%	100%	100%
Artery – Aorta	56.25%	38.46%	80%	100%
Artery – Tibial	53.85%	42.86%	100%	100%
Breast – Mammary	35.29%	55.17%	100%	100%
Cells – Transformed Fibroblasts	71.88%	55.26%	100%	100%
Esophagus – Mucosa	61.54%	62.50%	100%	100%
Esophagus – Muscularis	72.09%	20.00%	83.33%	85.71%
Heart – Left Ventricle	61.11%	79.49%	100%	100%
Lung	91.43%	30.00%	100%	100%
Muscle – Skeletal	69.70%	80.77%	100%	100%
Nerve – Tibial	69.57%	53.85%	100%	100%
Skin – Not Sun Exposed	70.00%	42.86%	100%	100%
Skin – Sun Exposed	41.67%	43.75%	100%	100%
Thyroid	75.00%	72.73%	100%	100%
Whole Blood	67.44%	79.31%	100%	100%

Table 6. Differential expression in tissue-specific hubs. Here, we consider a hub (rank ≤ 100) is tissue-specific if it is not present in top 500 hubs of any other tissues. This table shows the percentage of tissue-specific hubs, categorized by hub type, with at least a 1.5 fold expression level change between the tissue of interest and all other tissues. Here, NA means there was no tissue-specific hub for corresponding category.

Tissue	Unique Expression	Differential Expression	Other
Adipose – Subcutaneous	1.49%	66.58%	31.93%
Adipose – Visceral	2.99%	60.98%	36.04%
Artery – Aorta	4.22%	64.08%	31.71%
Artery – Tibial	1.98%	71.88%	26.14%
Breast – Mammary	4.24%	52.92%	42.85%
Cells – Transformed Fibroblasts	17.56%	70.97%	11.47%
Esophagus – Mucosa	6.65%	75.35%	18.01%
Esophagus – Muscularis	3.90%	61.98%	34.13%
Heart – Left Ventricle	14.58%	77.83%	7.59%
Lung	9.89%	61.62%	28.49%
Muscle – Skeletal	8.53%	83.08%	8.40%
Nerve – Tibial	4.13%	74.01%	21.86%
Skin – Not Sun Exposed	3.91%	70.86%	25.23%
Skin – Sun Exposed	2.45%	71.11%	26.44%
Thyroid	5.29%	77.29%	17.41%
Whole Blood	18.61%	77.41%	3.97%

Table 7. Sources of tissue-specificity of edges. *Unique Expression*: Both nodes connected by an edge were jointly included in TWN reconstruction of the tissue of interest only i.e., at least one of the nodes were excluded in every other tissue due to low expression or other filtering criteria. *Differential Expression*: Both nodes connected by an edge were jointly included in TWN reconstruction of multiple tissues and at least one of the nodes was differentially expressed (at least 1.5 fold change in raw TPM) between the tissue of interest and rest of the tissues. *Other*: Any other source.

Tissue	Nodes	Edges	Mean deg	# small hubs	# hubs	# large hubs	# tissues
Adipose – Visceral	17	64	7.53	12	8	0	1
Artery – Aorta	79	304	7.7	45	22	1	1
Artery – Coronary	40	107	5.35	19	7	0	1
Brain – Amygdala	18	29	3.22	4	1	0	1
Brain – Anterior	23	34	2.96	4	1	0	1
Brain – Caudate	73	438	12	38	32	1	1
Brain – Cerebellar Hemisphere	23	66	5.74	12	4	0	1
Brain – Cortex	14	30	4.29	5	2	0	1
Brain – Frontal Cortex	16	24	3	2	1	0	1
Brain – Hypothalamus	19	48	5.05	10	2	0	1
Brain – Nucleus Accumbens	8	8	2	1	0	0	1
Brain – Putamen	56	466	16.64	42	34	0	1
Brain – Spinal Cord	15	40	5.33	9	1	0	1
Brain – Substantia Nigra	8	10	2.5	0	0	0	1
Breast – Mammary	50	382	15.28	40	29	0	1
Cells – EBV-transformed lymphocytes	7	8	2.29	0	0	0	1
Esophagus – Gastroesophageal Junction	66	564	17.09	49	38	0	1
Esophagus – Muscularis	9	11	2.44	1	0	0	1
Lung	6	15	5	6	0	0	1
Muscle – Skeletal	14	16	2.29	0	0	0	1
Pancreas	15	29	3.87	4	0	0	1
Pituitary	7	8	2.29	0	0	0	1
Prostate	7	5	1.4	0	0	0	1
Stomach	12	18	3	0	0	0	1
Thyroid	22	56	5.1	10	3	0	1
Whole Blood	34	115	6.76	17	11	0	1
Adipose	13	51	7.8	12	5	0	2
Adipose and Mammary	46	144	6.26	27	12	0	3
Arteries	15	20	2.7	2	0	0	3
Artery and Heart	35	76	4.34	12	3	0	5
Brain	648	18854	58.19	532	467	207	10
Colon	25	38	3	6	1	0	2
Esophagus	87	855	19.7	59	52	9	3
Glands	22	65	5.9	12	4	0	6
Skins	11	36	6.5	9	1	0	2
Stomach and Colon	26	42	3.23	7	0	0	4

Table 8. Summary of tissue-specific networks (TSNs). Columns include tissues, total nodes, total edges, mean node degree, number of small hubs, number of hubs, number of large hubs, and the total number of tissues that make up the network.

TSN	Tissue-Specific GO Terms
Adipose – Visceral	Positive regulation of interleukin-6 production; Androgen receptor signaling pathway; Intracellular steroid hormone receptor signaling pathway; Adaptive immune response
Artery – Aorta	Aorta morphogenesis; Artery morphogenesis; Heart morphogenesis; Regulation of heart rate; Vascular smooth muscle contraction; Regulation of secondary heart field cardioblast proliferation; Cardiac cell fate determination; Cardiac muscle contraction; Epithelial to mesenchymal transition involved in endocardial cushion formation; Positive regulation of cardiac muscle tissue morphogenesis
Artery – Coronary	Blood Circulation
Brain – Amygdala	Bergmann glial cell differentiation; Membrane depolarization; Regulation of membrane repolarization during action potential; Regulation of neurotransmitter secretion; Axon guidance
Brain – Anterior Cingulate Cortex	Neuron migration; Negative regulation of synaptic transmission
Brain – Cerebellar Hemisphere<br>Brain – Cerebellar Hemisphere	Dopamine receptor signaling pathway; forebrain development; Cerebellar Purkinje cell layer development; Midbrain development; Pyramidal neuron development; Fear response; Neuroblast division in subventricular zone; Pons development; Positive regulation of dopamine uptake involved in synaptic transmission; Hypothalamus development; Behavioral response to ethanol; Inhibitory postsynaptic potential; Hippocampus development; Regulation of dopamine metabolic process; Rhalamus development; Behavioral response to cocaine; Synaptic transmission, dopaminergic; Adenylate cyclase-inhibiting dopamine receptor signaling pathway; Positive regulation of neurogenesis; Axonogenesis; Subthalamus development; Neuroblast proliferation; Dopamine metabolic process; Response to amphetamine
Brain – Cortex	Response to pain; Positive regulation of glial cell proliferation; Intracellular steroid hormone receptor signaling pathway; Glial cell migration; Response to drug; Response to testosterone; Response to axon injury; Behavioral response to pain
Brain – Frontal Cortex	Response to drug; Behavioral response to pain; Modulation of synaptic transmission; Nervous system development; Glial cell migration; Response to axon injury; Positive regulation of long-term synaptic potentiation; Positive regulation of glial cell proliferation
Brain – Hypothalamus	Regulation of cytosolic calcium ion concentration; Nerve development
Brain – Nucleus Accumbens	Response to progesterone; Response to testosterone; Positive regulation of glial cell proliferation; Behavioral response to pain; Glial cell migration; Response to axon injury; Response to drug
Brain – Spinal Cord	Segment specification; Skeletal system morphogenesis
	Regulation of axonogenesis; Neuron cell-cell adhesion; Neurotransmitter loading into synaptic vesicle; Serotonin transport; Subthalamus development; Sequestering of neurotransmitter;

Brain – Substantia Nigra	Aminergic neurotransmitter loading into synaptic vesicle; Neuron differentiation; Response to hormone; Neurotransmitter transport; Nervous system development; Pyramidal neuron development; Response to drug; Midbrain development, Pons development; Response to pain; Cerebellar Purkinje cell layer development
Breast – Mammary	Negative regulation of DNA damage response: signal transduction by p53 class mediator; Response to mechanical stimulus; Negative regulation of cytokine production involved in inflammatory response; Positive regulation of transforming growth factor beta receptor signaling pathway
Cells – EBV-transformed lymphocytes	T cell differentiation; Heterotypic cell-cell adhesion; T cell costimulation; Cell surface receptor signaling pathway; Positive thymic T cell selection; Immune system process; Defense response to virus; Adaptive immune response; T cell activation; Leukocyte migration; Immune response; Cytotoxic T cell differentiation; Natural killer cell activation
Esophagus – Muscularis	Smooth muscle contraction; Muscle contraction; Elastic fiber assembly
Lung	Viral entry into host cell; Transport; Mucus secretion
Muscle – Skeletal	Actin cytoskeleton organization; Oxidation-reduction process; Toxin transport; Response to nutrient;
Muscle – Skeletal	Cellular response to peptide hormone stimulus; Positive regulation of steroid hormone biosynthetic process
Pancreas	Regulation of insulin secretion involved in cellular Response to glucose stimulus; Response to food; Response to dietary excess
Pituitary	Postsynaptic membrane assembly; Neuron differentiation; Negative regulation of axonogenesis; Secretion; Cell surface receptor signaling pathway; Synapse organization; Transmembrane receptor protein tyrosine kinase signaling pathway
Stomach	Muscle contraction
Whole Blood	Blood coagulation; Immune system process; Transport; Leukocyte cell-cell adhesion

Table 9. Enriched tissue-relevant gene ontology biological process terms in nodes of individual TSNs (Fisher's exact test; all $p \leq 0.05$ BH corrected).

Tissue	Variant	trans-eGene	cis-eGene	p-value	FDR
Brain – Frontal Cortex	rs11065155	*COX5B*	*TRIAP1*	0.02	0.09
Brain – Anterior Cingulate Cortex	rs470411	*MAGOH*	*TRIM29*	3.36×10^{-3}	0.02
Brain – Cerebellar Hemisphere	rs66500423	*UQCRQ*	*NUMBL*	2.36×10^{-2}	0.15
Brain – Cerebellar Hemisphere	rs66500423	*UNC50*	*NUMBL*	3.23×10^{-2}	0.15
Brain – Putamen	rs9371531	*CHCHD1*	*RMND1*	6.99×10^{-4}	0.06
Thyroid	rs934937	*BRCA1*	*C15orf52*	0.00299	0.09

Table 10. Summary of tissue-specific trans-eQTLs from the cis-eQTL enrichment tests in the TSNs. Columns include tissues, the RSID of the associated genetic variant, the trans-eGene, the cis-eGene, the p-value of the trans-eQTL association, and the FDR of this association.

Tissue	Variant	trans-eGene	cis-eGene	p-value	FDR
Pituitary	rs36077494	*PTPRT*	*KIRREL*	4.66×10^{-3}	0.16
Pancreas	rs16913469	*RNF38*	*DDIT4*	4.61×10^{-4}	0.15
Muscle – Skeletal	rs11121453	*SLC7A8*	*NPHP4*	3.54×10^{-4}	0.15
Brain – Substantia Nigra	rs911110	*UQCRQ*	*PCNA*	1.07×10^{-3}	0.10
Brain – Hypothalamus	rs116850387	*LAMTOR2*	*TRIAP1*	1.30×10^{-3}	0.16
Brain – Hypothalamus	rs73221368	*DSCR3*	*TRIAP1*	1.72×10^{-3}	0.16
Brain – Hypothalamus	rs73221368	*LAMTOR2*	*TRIAP1*	1.76×10^{-3}	0.16
Brain – Hypothalamus	rs73216931	*ILK*	*RILPL2*	1.02×10^{-4}	0.16
Brain – Hypothalamus	rs9974252	*TRIAP1*	*DSCR3*	9.24×10^{-4}	0.16
Brain – Hypothalamus	rs28642307	*RILPL2*	*BOLA1*	1.42×10^{-3}	0.16
Brain – Hypothalamus	rs10742976	*LAMTOR2*	*ILK*	4.91×10^{-4}	0.16
Brain – Hypothalamus	rs960177	*RILPL2*	*CALB2*	9.55×10^{-4}	0.16
Brain – Frontal Cortex	rs2347443	*MAGOH*	*HSCB*	1.33×10^{-4}	0.13
Brain – Cortex	rs45567235	*TRIAP1*	*CCDC107*	5.73×10^{-5}	0.09

Table 11. Summary of tissue-specific trans-eQTLs from the 20 kb tests in the TSNs. Columns include tissues, the RSID of the associated genetic variant, the trans-eGene, the cis-eGene, the p-value of the trans-eQTL association, and the FDR of this association. Only the most significant trans-eVariant per cis-eGene and trans-eGene pair is included in the table.

**Tissue**	**Number of unique genes**
Adipose – Subcutaneous	4423
Adipose – Visceral	4416
Artery – Aorta	4345
Artery – Tibial	4378
Breast – Mammary	4495
Esophagus – Mucosa	4443
Esophagus – Muscularis	4381
Cells – Transformed Fibroblasts	4341
Heart – Left Ventricle	3849
Lung	4647
Nerve – Tibial	4526
Muscle – Skeletal	4048
Skin – Not Sun Exposed	4546
Skin – Sun Exposed	4542
Thyroid	4565
Whole Blood	3762

Table 12. Number of unique genes included in IR nodes of TWNs. We selected 9000 isoforms to reconstruct a TWN for each tissue. This table shows the number of unique genes to which the selected isoforms belong (per tissue).

References

Alexa A and Rahnenfuhrer J, 2016. topGO: Enrichment Analysis for Gene Ontology.

Andrews S, 2010. FastQC: a quality control tool for high throughput sequence data.

Bolger AM, Lohse M, and Usadel B, 2014. Trimmomatic: A flexible trimmer for Illumina sequence data. *Bioinformatics*, :btu170.

Derrien T, Estellé J, Sola SM, Knowles DG, Raineri E, Guigó R, and Ribeca P, 2012. Fast computation and applications of genome mappability. *PLoS ONE*, **7**(1).

Dobin A, Davis CA, Schlesinger F, Drenkow J, Zaleski C, Jha S, Batut P, Chaisson M, and Gingeras TR, 2013. STAR: Ultrafast universal RNA-seq aligner. *Bioinformatics*, **29**(1):15–21.

The GTEx Consortium , 2015. The Genotype-Tissue Expression (GTEx) pilot analysis: Multitissue gene regulation in humans. *Science*, **348**(6235):648–660.

Howie BN, Donnelly P, and Marchini J, 2009. A flexible and accurate genotype imputation method for the next generation of genome-wide association studies. *PLoS Genetics*, **5**(6).

Lachmann A, Xu H, Krishnan J, Berger SI, Mazloom AR, and Ma'ayan A, 2010. ChEA: transcription factor regulation inferred from integrating genome-wide ChIP-X experiments. *Bioinformatics*, **26**(19):2438–2444.

Li B and Dewey CN, 2011. RSEM: accurate transcript quantification from RNA-Seq data with or without a reference genome. *BMC Bioinformatics*, **12**(1):1.

McDowell IC, Pai AA, Guo C, Vockley CM, Brown CD, Reddy TE, and Engelhardt BE, 2016. Many long intergenic non-coding RNAs distally regulate mRNA gene expression levels. *bioRXiv*, doi:10.1101/044719.

Subramanian A, Tamayo P, Mootha VK, Mukherjee S, Ebert BL, Gillette MA, Paulovich A, Pomeroy SL, Golub TR, Lander ES, *et al.*, 2005. Gene set enrichment analysis: A knowledge-based approach for interpreting genome-wide expression profiles. *Proceedings of the National Academy of Sciences*, **102**(43):15545–15550.
